# Kaposi's Sarcoma Associated Herpes Virus (KSHV) Induced COX-2: A Key Factor in Latency, Inflammation, Angiogenesis, Cell Survival and Invasion

**DOI:** 10.1371/journal.ppat.1000777

**Published:** 2010-02-12

**Authors:** Neelam Sharma-Walia, Arun George Paul, Virginie Bottero, Sathish Sadagopan, Mohanan Valiya Veettil, Nagaraj Kerur, Bala Chandran

**Affiliations:** H. M. Bligh Cancer Research Laboratories, Department of Microbiology and Immunology, Chicago Medical School, Rosalind Franklin University of Medicine and Science, North Chicago, Illinois, United States of America; Oregon Health and Science University, United States of America

## Abstract

Kaposi's sarcoma (KS), an enigmatic endothelial cell vascular neoplasm, is characterized by the proliferation of spindle shaped endothelial cells, inflammatory cytokines (ICs), growth factors (GFs) and angiogenic factors. KSHV is etiologically linked to KS and expresses its latent genes in KS lesion endothelial cells. Primary infection of human micro vascular endothelial cells (HMVEC-d) results in the establishment of latent infection and reprogramming of host genes, and cyclooxygenase-2 (COX-2) is one of the highly up-regulated genes. Our previous study suggested a role for COX-2 in the establishment and maintenance of KSHV latency. Here, we examined the role of COX-2 in the induction of ICs, GFs, angiogenesis and invasive events occurring during KSHV de novo infection of endothelial cells. A significant amount of COX-2 was detected in KS tissue sections. Telomerase-immortalized human umbilical vein endothelial cells supporting KSHV stable latency (TIVE-LTC) expressed elevated levels of functional COX-2 and microsomal PGE2 synthase (m-PGES), and secreted the predominant eicosanoid inflammatory metabolite PGE2. Infected HMVEC-d and TIVE-LTC cells secreted a variety of ICs, GFs, angiogenic factors and matrix metalloproteinases (MMPs), which were significantly abrogated by COX-2 inhibition either by chemical inhibitors or by siRNA. The ability of these factors to induce tube formation of uninfected endothelial cells was also inhibited. PGE2, secreted early during KSHV infection, profoundly increased the adhesion of uninfected endothelial cells to fibronectin by activating the small G protein Rac1. COX-2 inhibition considerably reduced KSHV latent ORF73 gene expression and survival of TIVE-LTC cells. Collectively, these studies underscore the pivotal role of KSHV induced COX-2/PGE2 in creating KS lesion like microenvironment during de novo infection. Since COX-2 plays multiple roles in KSHV latent gene expression, which themselves are powerful mediators of cytokine induction, anti-apoptosis, cell survival and viral genome maintainence, effective inhibition of COX-2 via well-characterized clinically approved COX-2 inhibitors could potentially be used in treatment to control latent KSHV infection and ameliorate KS.

## Author Summary

Kaposi's sarcoma associated herpes virus (KSHV), with a 160 kb DNA genome, has evolved with two distinct life cycle phases, namely latency and lytic replication. KS, a complex angioproliferative disease, is regulated by a balance between pro-angiogenic and anti-angiogenic factors. In our previous study, we showed that KSHV modulates host factors COX-2/PGE2 for its own advantage to promote its latent (persistent) infection. The premise that COX-2 is involved in growth and progression of several types of solid cancers and inflammation associated diseases has been well documented but has never been studied in KS. Here, utilizing COX-2 inhibition strategies, including chemical inhibition and a gene silencing approach, we systematically identified the potential role of KSHV induced COX-2/PGE2 in viral pathogenesis related events such as secretion of inflammatory and angiogenic cytokines, MMPs and cell adhesion in de novo infected or latently infected endothelial cells. We report that COX-2/PGE2 inhibition down-regulates viral latent gene expression and survival of latently infected endothelial cells. The data emanating from our *in vitro* studies is valuable, informative and requires further examination using an *in vitro* angiogenic model and *in vivo* nude mice model to further validate COX-2 as a novel therapeutic to target latent infection and the associated diseases like KS.

## Introduction

KSHV, the most recently discovered human tumor virus, is etiologically associated with Kaposi sarcoma (KS), primary effusion lymphoma (PEL) and multicentric Castleman's disease (MCD) [1],[2]. KS, an AIDS defining condition, is a highly disseminated unusual angiogenic tumor of proliferative endothelial cells and displays a very strong resemblance to chronic inflammation [1],[2],[3],[4]. KS is responsible for significant morbidity and mortality in HIV-infected patients in the developing world [1],[2]. KS lesions are characterized by proliferating spindle shaped endothelial cells, neo-vascular structures, inflammatory cells, and an abundance of inflammatory cytokines (ICs), growth factors (GFs), angiogenic factors and invasive factors such as basic and acidic fibroblast growth factor (bFGF, aFGF), interleukin-1α and β (IL-1α and -1β), granulocyte-monocyte colony stimulating factor (GM-CSF), platelet derived growth factor β (PDGF-β), vascular endothelial growth factor (VEGF), interferon-γ (IFNγ), interlukin 6 (IL-6), tumor necrosis factor α (TNF-α) [2], angiopoietin-2 (Ang2) [5], angiogenin [6], heme oxygenase-1 (HO-1) [7], transforming growth factor β (TGF-β) [8], adhesion molecules like inter-cellular adhesion molecule 1 (ICAM-1) and vascular cell adhesion molecule-1(VCAM-1), and matrix metalloproteinases (MMPs) like MMP-1, -2, -3, -9, and -19. Cell cultures composed of characteristic spindle-shaped tumor cells have been established from KS lesion explants by the addition of cytokines like TNF-α, TNF-β, IFN-γ, IL-1, IL-6, GM-CSF and oncostatin M [1],[2],[9],[10] highlighting the role of these paracrine factors in KS lesion cell survival. A crucial step in KS progression is its striking neovascularization and angiogenesis, which is regulated by aberrant production of angiogenic and anti-angiogenic factors from the infected host cells, uninfected neighboring cells or both [11]. It is believed that KSHV tumorigenesis and disease progression are predominantly driven by both paracrine and autocrine mechanisms, where KSHV infection could induce an angiogenic, GFs-, and MMPs- rich microenvironment and a strong cytokine network. These events, via their synergistic actions and communications, could support continued proliferation and migration of KSHV latently infected cells [2],[12].

KSHV encodes ∼86 putative open reading frames (ORFs) of which at least 22 are potentially immuno-modulatory and anti-apoptotic [13],[14]. Among these are the genes “pirated” from the host or cellular homologues like viral G-protein-coupled receptor (vGPCR), vIL-6, viral interferon regulatory factors (vIRFs 1-4), viral chemokines (vCCLs 1-3), MHC class I down-regulating E3 ligases K3 and K5 (MIR1 and MIR2), and Kaposin B [13],[14],[15],[16],[17],[18],[19]. These proteins are capable of regulating cellular cytokine expression, antagonizing host IFN mediated anti-viral responses and immune evasion, thus suggesting the importance of ICs in KSHV-associated pathogenesis [20]. In KS lesion endothelial cells, KSHV is in a latent form with about 10–20 copies of the viral episome per cell and lytic replication is observed in a low percentage of infiltrating inflammatory monocytes. Low percentages of KSHV-infected cells in KS lesions are typical spindle cells which are thought to represent neoplastic cells in these lesions and these cells occassionaly express lytic gene products, undergo lytic reactivation and may support productive replication [21]. During latency, KSHV expresses a battery of genes such as ORF73 (LANA-1), ORF72 (vCyclin), ORF71 (K13/vFLIP), and ORFK12 (Kaposin A, B and C), as well as 12 distinct miRNAs. These gene products obviously must be facilitating the establishment of lifelong latency in its host and in survival against the host intrinsic, innate and adaptive immune surveillance mechanisms [22],[23],[24].

Cytokines have been shown to play important roles in viral immune evasion and lytic replication. ICs like IL-1β, IL-6, and TNF-α have been shown to inhibit KSHV lytic gene transcription in endothelial cells [20]. Host immune responses against KSHV control viral replication and viral spread and exert a selective pressure on the virus to establish a latent state which allows the virus to evade the subsequent wave of adaptive immune host responses following an effective innate immune response. Therefore, studying KSHV infection linked cytokines is relevant to understand viral multifactor patho-biology, its mechanisms to induce neoplasia, and for developing therapeutic interventions.

Apart from viral genes, this virus has also evolved strategies to regulate host gene expression to create a microenvironment that is conducive for viral persistence. One of the host genes that is highly induced upon de novo infection of human microvascular endothelial cells (HMVEC-d) and human foreskin fibroblast (HFF) cells is cyclooxygenase-2 (COX-2) [25],[26]. KSHV-encoded early lytic-cycle membrane protein vGPCR and cell–cell contact deregulator protein K15 have also been shown to trigger COX-2 induction [27],[28]. COX, the rate limiting enzyme of prostaglandin synthesis has three isoforms identified to date, namely COX-1, COX-2, and COX-3. COX-1 is constitutively expressed and displays characteristics of a housekeeping gene in most tissues. In contrast, COX-2 is a key enzyme for prostanoid biosynthesis [29],[30]. COX-2 possesses pro-angiogenic, anti-apoptotic properties and is up-regulated by mitogenic and inflammatory stimuli [29],[30]. COX-2 has also been implicated in the progression and angiogenesis of several cancers [30],[31], and is widely regarded as a potential pharmacological target for preventing and treating malignancies [31],[32],[33].

In our earlier studies, we demonstrated robust COX-2 gene expression and high levels of PGE2 secretion by KSHV during primary infection of HMVEC-d and HFF cells [26]. Inhibition of COX-2 by NS-398 and indomethacin (Indo) did not affect KSHV binding, internalization of virus, or it's trafficking to the infected cell nuclei [26]. Intriguingly, latent ORF73 promoter activity and gene expression were significantly reduced by COX-2 inhibitors, and this inhibition was relieved by exogenous supplementation with PGE2 [26]. In contrast, lytic ORF50 gene expression and ORF50 promoter activity were unaffected indicating that KSHV has evolved to utilize COX-2 mediated inflammatory responses induced during infection of endothelial cells for the maintenance of viral latent gene expression [26].

Since COX-2 is linked to inflammation and KS is a chronic inflammation associated malignancy, we hypothesized that COX-2 is one of the virus's triggered pathogenic factors with key roles in inflammation, neo-angiogenesis, cell proliferation, and invasion associated with the KS lesions. When we tested this hypothesis by using chemical inhibitors of COX-2 or by COX-2 silencing, we uncovered evidence for the role of COX-2/PGE2 in viral latent gene expression, in pro-inflammatory, angiogenic and invasive events occurring during KSHV de novo infection of endothelial cells as well as the survival of latently infected endothelial cells. Effective reduction in secretion of autocrine and paracrine factors involved in KSHV pathogenesis during early and later time points of infection, along with cell cycle arrest observed in latently infected endothelial cells, suggested that COX-2 inhibition based therapy might provide an effective way to treat the angio-proliferative KS lesions.

## Materials and Methods

### Cells

HMVEC-d (CC-2543; Lonza Walkersville, Maryland) were cultured in endothelial basal medium 2 (EBM-2) with growth factors (Lonza Walkersville). HEK 293T (human embryonic kidney cells stably expressing SV40 large T-antigen) cells were grown in Dulbecco's modified Eagle's medium (Gibco BRL, Grand Island, New York) supplemented with 10% heat-inactivated fetal bovine serum (HyClone, Logan, UT), 2 mM L-glutamine, and antibiotics [26],[34],[35]. HUVECs (Lonza Walkersville) were cultivated in EGM-2 (Lonza Walkersville). Cells were typically used between 5 to 7 passages. TIVE (telomerase-immortalized human umbilical vein endothelial) and TIVE-LTC (long-term-infected TIVE) cells (a gift from Dr. Rolf Renne, Department of Molecular Genetics and Microbiology, University of Florida) were cultured in EBM-2 with growth factors. All cells were cultured in LPS-free medium. All stock preparations of purified KSHV were monitored for endotoxin contamination by standard *Limulus* assay (*Limulus* amebocyte lysate endochrome; Charles River Endosafe, Charleston, S.C.) as recommended by the manufacturer [26]. The COX-1 and COX-2 inhibitor Indomethacin and the COX-2-specific inhibitor NS-398 [*N*-(2-cyclohexyloxy-4-nitrophenyl)-methanesulfonamide] were purchased from Calbiochem, La Jolla, Calif. Both inhibitors were reconstituted in dimethyl sulfoxide (DMSO) and DMSO was used as solvent control for all experiments involving treatments with inhibitors.

### Virus

Induction of the KSHV lytic cycle in BCBL-1 cells, supernatant collection, and virus purification procedures were described previously [26]. KSHV DNA was extracted from the virus, and the copy numbers were quantitated by real-time DNA PCR using primers amplifying the KSHV ORF 73 gene as described previously [26],[34],[35].

### Lentivirus production and transduction of HMVEC-d cells

Lentiviral constructs expressing shRNAs against human COX-2 and control laminA/C were generated as described [36]. These shRNA transcription products are known to be processed by the cell to produce the functional siRNA sequence. AACTGCTCAACACCGGAAT (si-COX-2-1) and CACCATCAATGCAAGTTCT (si-COX-2-2) sequences were used as COX-2 shRNAs. Testing for reduction by shRNA constructs was done by transfection of target plasmids and shRNA lentiviral construct plasmids into 293T cells followed by protein extraction and immunoblot analysis to select the best candidates. Third generation lentiviral vectors were produced using a four-plasmid transfection system as previously described [36]. Briefly, 293T cells were transfected with vector and packaging plasmids. Culture supernatant was harvested 2 and 3 days post-transfection. Cell debris from the supernatant was cleared by filtration through 0.22-µm filters, concentrated by ultracentrifugation, and lentiviral vector titers were estimated by flow cytometery (eGFP expression). HMVEC-d cells were transduced with either si-COX-2 or si-lamin (si-C) to produce si-COX-2-HMVEC-d or si-C-HMVEC-d.

### Gene expression profiling by quantitative real time-PCR

Total RNA was converted to cDNA, relative abundance of target gene mRNA was measured by qRT-PCR using the delta-delta method (ratio, 2^−[ΔCt sample–ΔCt control]^) as decribed previously [37]. Primer sequences are given in Table S2. PCR amplifications without cDNA were performed as negative controls.

### Immunofluorescence assay (IFA)

Confluent HMVEC-d cells in eight-well chamber slides (Nalge Nunc International, Naperville, Il.) were either uninfected or infected (30 DNA copies/ cell) for 24h. For COX-2 and VEGF-A immunostaining, cells were fixed with 4% paraformaldehyde (PFA), permeabilized with 0.4% Triton-X 100 and stained with anti-COX-2 goat polyclonal antibody (Cayman chemical, Ann Arbor, Mich.) and anti-VEGF-A monoclonal antibody (Santa Cruz Biotechnology, Inc., Santa Cruz, CA) overnight at 4°C. Cells were washed and incubated with 1∶200 dilution of Alexa 594-coupled anti-mouse antibody or Alexa 488-coupled anti-goat antibody (Molecular Probes, Eugene, OR) for 1 h at RT. Nuclei were visualized by using DAPI (Ex358/Em461; Molecular Probes) as counter stain. Stained cells were washed and viewed with appropriate filters under a fluorescence microscope with the Nikon metamorph digital imaging system. HMVEC-d cells were either uninfected or infected (30 DNA copies/ cell) for 2 h, 4 and 5 days and stained for KSHV latency protein ORF73 (5d) and lytic protein ORF59 (2 h, 4d and 5d) using antibodies generated in Prof. Bala Chandran's laboratory. TIVE and TIVE-LTC cells were also co-stained for ORF73 and COX-2 using the above mentioned procedures.

### Immunohistochemistry

Sections from lymph nodes and skin biopsy samples of healthy subjects and KS+ patients were obtained from the AIDS and Cancer Specimen Resource (ACSR). Sections were deparaffinized with Histochoice clearing reagent and hydrated with water before microwave treatment in 1 mmol/l EDTA (pH 8.0) for 15 min for antigen retrieval, and then blocked with blocking solution (2% donkey serum, and 0.3% Triton X-100 in PBS). Sections were incubated with the primary antibodies against COX-2 (Cell signaling technology Inc.) or ORF73 (generated in Prof. Bala Chandran's laboratory) overnight at 4°C. These sections were incubated with rat-polymer-HRP (Biocare medical) for 15 min, washed and developed using DAB reagent (DAKO). Counterstaining was done by hematoxylin. Similar procedure was followed for COX-2 staining of ACSR KS Screening tissue microarray (TMA) 09-1 (Table S1).

### Immunofluorescence staining of paraffin embedded tissue sections

Sections from lymph nodes and skin biopsy samples of KS+ patients and control samples were deparaffinized and hydrated with water before antigen retrieval using DAKO target retriever solution in steamer for 20 min. Slides were cooled, rinsed, blocked using 1% BSA in 0.025% Triton X-100-PBS for 30 min and used for double staining of COX-2 and monoclonal mouse anti-human CD31 (DAKO, Denmark). Sections were washed and incubated with 1∶200 dilution of Alexa 594-coupled anti-mouse antibody or Alexa 488-coupled anti-rabbit antibody (Molecular Probes) for 1h at RT. Nuclei were visualized using DAPI and stained cells were viewed under an Olympus Confocal laser scanning microscope (Fluoview FV10i).

### Cytokines and Matrix-metalloproteinase (MMP) analysis

Conditioned medium was obtained from serum-starved, untreated, Indo, NS-398-pretreated HMVEC-d, si-COX-2-HMVEC-d or si-C-HMVEC-d cells either uninfected or KSHV (30 DNA copies/ cell) infected for different time points. Conditioned media were spun at 1,000 rpm for 10′ at 4°C to remove the particulates and assayed immediately. Total soluble protein was quantified by bicinchoninic acid (BCA) protein assay (Pierce, Rockford, IL) prior to use ensuring equal protein concentration for studying the cytokine profile by human protein cytokine arrays 3.1 and 5.1 from Ray Biotech (Norcross, GA) and Ray Biotech human MMP antibody array-1 which detects 10 human MMPs in one experiment. Uninfected HMVEC-d/si-COX-2-HMVEC-d/si-C-HMVEC-d cells were used as controls for KSHV infected HMVEC-d/si-COX-2-HMVEC-d/si-C-HMVEC-d cells, respectively. The cytokine detection membranes were blocked with blocking buffer for 1 h at RT and then incubated with conditioned media at 4°C overnight. The membranes were washed, incubated with 1 ml of primary biotin-conjugated antibody at RT for 2h, washed, incubated with 2 ml of horseradish peroxidase-conjugated streptavidin at RT for 45′, and developed using enhanced-chemiluminescence (ECL). Signal intensities were quantitated using an Alpha Inotech image analysis system. Signal intensities from all the arrays were normalized to the same background levels with positive and negative controls using Ray Biotech human antibody array 3.1/5.1 and MMP antibody array-1 analysis software.

### Matrix-metalloproteinase activity assay

Conditioned media used for the MMP detection by MMP-antibody array were also used for determination of active/total MMP-2 and MMP-9 using MMP-2 and MMP-9-enzyme-linked immunosorbent assay (ELISA) kits from Anaspec (San Jose, CA) as per manufacturer's protocols. These kits were optimized to detect levels of total MMPs and their activities using a 5-FAM/QXL™520 FRET peptide as substrate with its fluorescence monitored at Ex/Em = 490 nm/520 nm upon proteolytic cleavage. These novel assays use FRET substrates that incorporate QXL™520 non-fluorescent dyes, the best quencher available for 5-FAM and are designed for the specific quantitation of the activity of a particular MMP in a mixed biological sample, which may contain multiple MMPs. A monoclonal anti-human-MMP antibody was used to pull down both the pro- and active- forms of an MMP from the mixture, and proteolytic activity quantitated using a 5-FAM/QXL™520 FRET peptide. Similarly, active/total MMP-2 and MMP-9 levels were detected in the conditioned media obtained from TIVE cells and COX inhibitor pretreated or untreated TIVE-LTC cells.

### Vascular endothelial growth factor -A and -C protein measurement

Conditioned medium, obtained as described was used for quantitating VEGF-A and -C levels using QuantiGlo ELISA kits (R and D Systems, Minneapolis, MN) as per procedures recommended by the manufacturer. Each sample was run in duplicate and the assay repeated a minimum of three times. Quantities of VEGF-A or VEGF-C released were normalized by protein content.

### ELISA for PGE2 detection

Levels of PGE2 in the supernatants of uninfected and KSHV infected HMVEC-d cells, and inhibitor treated or COX-2/lamin silenced and then uninfected or KSHV infected HMVEC-d cells, or TIVE and TIVE-LTC, COX inhibitor treated or untreated TIVE-LTC cells were measured by ELISA (Cayman Chemicals) according to the manufacturer's instructions [26]. Data are expressed as the amount of PGE2 produced (pg/ml) per 10^5^ cells.

### In vitro capillary tube formation assay

Conditioned media were collected from the variously treated cells for analysis on matrigel and the assay was performed as per manufacturer's instructions (BD Biosciences, Mountain View, CA). Briefly, 5×10^4^ HUVEC or HMVEC-d cells were plated on a Matrigel-coated 96-well plate with medium alone or medium obtained from cells treated with inhibitors alone or cells pretreated with inhibitors and then KSHV (30 DNA copies/ cell) infected or the cells silenced for COX-2 and then infected for 24 h. After 16 h in 5% CO_2_ at 37°C, the plate was examined for capillary tube formation under an inverted microscope and photographed. Each assay was done in duplicate and each experiment was repeated three times. Angiogenic index, a measure of tube formation, was calculated based on the number of branch points formed from each node per field at 10X original magnification. Differences between the numbers of tube formations in 3D-conditioned Matrigel assays were subject to student's *t*-test analysis. Similar assay was performed using the conditioned media obtained from 24 h serum starved TIVE cells and COX inhibitors or solvent pretreated or untreated TIVE-LTC cells.

### Western blot analysis

Cell extracts were quantitated by BCA protein assay, then equal amounts of protein (20 µg/lane) were separated on SDS-PAGE, electrotransferred to 0.45-µm nitrocellulose membranes. The membranes were blocked with 5% BSA, probed with anti-COX-2, active-Rac, total-Rac , β-actin and tubulin antibodies and visualized using an ECL detection system [26].

### Cell adhesion assay

Maxisorp II Nunc ELISA plates (Roskilde, Denmark) were coated with fibronectin (5 µg/ml), or poly-lysine (2.5 µg/cm^2^) overnight at 4°C and adhesion assays were performed. Briefly, HMVEC-d cells were resuspended in serum-free EBM-2 medium and plated at 3×10^4^ cells in 200 µl/well and incubated at 37°C in a 5% CO_2_ with 100% humidity. At given times, unattached cells were removed by rinsing the wells with warm (37°C) PBS. Attached cells were fixed in 4% PFA, stained with 0.5% crystal violet and quantified by reading OD at 595 nm.

### RhoA and Rac1-GTPase activity by G-LISA activation assay

This assay is based on the principle that a Rho and Rac-GTP-binding protein is linked to the 96-well plates (RhoA and Rac-1 GLISA from Cytoskeleton, Inc.). The active GTP-bound Rho or Rac-1 in the cell lysates binds to the wells, while the inactive GDP-bound Rho or Rac-1 is removed during the washing steps. The bound active RhoA or Rac-1 is detected with a RhoA or Rac-1 specific antibody and quantitated by absorbance. The degree of RhoA or Rac-1 activation is determined by comparing readings from lysates prepared from various treatments.

### Invasion assays

Invasion through the extracellular matrix (ECM), an important step in KSHV pathogenesis, was measured by two methods. **1)** *Innocyte cell invasion assay* was used to quantitate the invasive cells and is based on the principle that invasive cells would degrade the laminin layer and will migrate through the membrane and attach to the underside of the membrane. These invasive cells are dislodged from the underside of the cell culture insert and stained with a fluorescent dye in a single step and fluorescence is determined using a fluorimeter (Ex485/Em520 nm). Briefly, the upper chambers of Transwells (Corning Costar) precoated with ECMatrix was allowed to wet by incubating with serum free EBM-2. After an hour of hydration, 5×10^4^ cells (HMVEC-d, TIVE, TIVE-LTC or HMVEC-d cells treated with various conditioned media) were plated in the upper chambers. The lower chambers contained complete growth medium. The inserts were incubated for 24 h and the invading cells were quantitated by fluorimetry. **2)** *Chemicon cell invasion assay* was performed to further confirm the invasion of cells upon various treatments and the assay is based on staining the invasive cells on the lower surface of the membrane by dipping inserts into the staining solution, washing, drying the inserts and counting the cells by photographing the membrane through the microscope as described in the manufacturer's instructions. Both assays were used to assess the role of COX-2 in regulating the invasive potential of HMVEC-d, TIVE and TIVE-LTC cells. Human fibrosarcoma (HT-1080) cells with high invasive potential were used as positive control.

### Cell number and viability assays

The *in vitro* effects of COX-2 inhibition, serum withdrawl on TIVE and TIVE-LTC cell numbers, and viability were determined by traditional trypan blue staining (evaluation of cell membrane integrity) in quadruplicate. As trypan blue staining is not a sensitive method for quantitation, the number of viable cells with their metabolically active mitochondria (an index of cell proliferation) was also determined by the 3-(4,5-dimethylthiazol-2-yl)-2,5-diphenyl tetrazolium bromide (MTT)–based colorimetric assay (ATCC, Manassas, VA) as per the manufacturer's instructions. The MTT assay detects living but not dead cells and signal generated depends upon the degree of activation of these cells. Briefly, 0.5×10^5^ HMVEC-d, TIVE, and TIVE-LTC cells were allowed to grow in the presence of complete growth medium (EGM-2) or in basal medium without growth factors and serum (EBM-2) or EGM-2 containing the indicated amount of COX-inhibitor or solvent control for 24 h, 48 h, 72 h and 96 h. 10 µl of MTT Reagent was added to all the cells at the indicated time of treatment and further incubated for 4 h (development of insoluble purple precipitate), solubilized in detergent and then read at 570 nm. The amount of MTT (yellow tetrazolium salt), which is converted to insoluble purple formazan crystals represents the number of viable cells and the degree of conversion was assessed by measuring the absorbance at a wavelength of 570 nm.

### Cell cycle analysis by flow cytometry

TIVE or TIVE-LTC cells were either untreated or treated with drugs (Indo or NS-398) or solvent control as described for cell number and viability assays. Harvested cells were diluted to contain ∼10^6^ cells/ml and DNA distribution analysis was performed. Cells were fixed with 70% ethanol overnight and DNA was stained with propidium iodide at a final concentration of 50 µg/ml with RNaseA (100 U/ml) prior to flow cytometry analysis using a LSRII (BD Biosciences). Data were analyzed using ModFit Lt V3 software (Verity Software House).

## Results

### COX-2 is expressed in KS lesions

**Figure 1:**
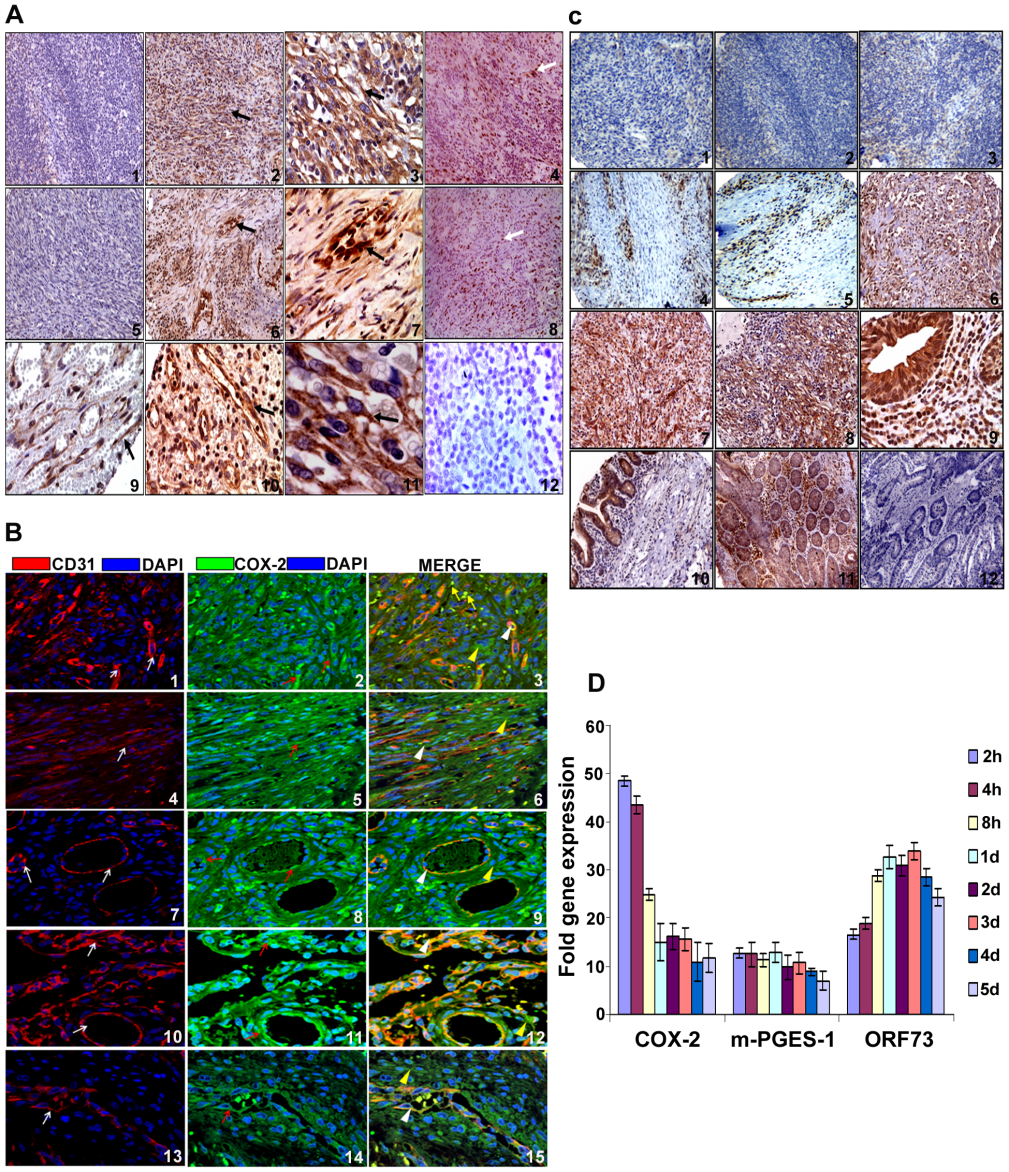
COX-2 expression. (**A**) **COX-2 expression in KS tissue.** Samples were analyzed by immunohistochemical staining for COX-2 (panels 1–3, 5–7, 9–11), anti-ORF73 (panels 4 and 8) and isotype control antibody alone (panel 12) and counterstained with hematoxylin. Arrow (black) in all the panels (2, 3, 6, 7, 9, 10 and 11) indicates COX-2 staining. Arrow (white) in panels 4 and 8 indicates ORF73 staining. Magnifications: panels 1, 2, 4–6 and 8: 20X; panels 3, 7, 9–12: 60X. Magnified views are given in **Figure S1**. (**B**) **COX-2 and CD31 double staining in KS skin and lymph node sections.** Samples were analyzed by immunofluoresence staining for COX-2 and CD31 staining in KS skin (panels 1–6) and lymph node sections (panels 7–15). Red arrows indicate COX-2 (green) staining (panels 2, 5, 8, 11 and 14). White arrows indicate CD31 (red) staining (panels 1, 4, 7, 10 and 13). White arrow heads indicate areas of COX-2 and CD31 co-staining in the same cells (panels 3, 6, 9, 12 and 15). Yellow arrow heads indicate cells staining for COX-2 but not CD31 (panels 3, 6, 9, 12 and 15). Yellow arrows depict COX-2 staining cells with no spindle phenotype or endothelial cell morphology (panel 3). (**C**) **COX-2 expression in various tissues of KS patients.** ACSR-KS Screening TMA 09-1 tissue sections had sections from skin (panels 1, 2, 4 and 5), mouth (panels 3 and 8), eye orbit (panel 6), tonsil (panel 7) small bowel (panels 9 and 10) as well as tongue, anus, nasopharynx, rectal mucosa, epiglottis, hypopharynx, soft tissue mass, lung and gastric mucosa (**Table S1**). Brown color indicates COX-2 staining (panels 1 to 10). Panels 11 and 12 are colon cancer tissue stained with COX-2 antibody or IgG control for COX-2 antibody, respectively. Magnifications: panels 1–8: 20X; panels 9–12: 60X. (**D**) **Induction of COX-2 and m-PGES-1 during de novo KSHV infection.** HMVEC-d cells grown to 80–90% confluence were serum starved for 8h and infected with 30 DNA copies/ cell of KSHV. Total RNA from infected and uninfected cells was DNase-1 treated and subjected to q-RT-PCR using ORF73, COX-2 and m-PGES-1 gene specific primers using the ΔΔCt method. Ct value obtained for ORF73 in uninfected cells (mostly close to non template control) was used for calculating fold inductions. Fold induction was calculated by considering induction in uninfected cells at respective times as 1-fold. Each bar represents the average ± SD of three independent experiments.

Despite clinical and epidemiological differences, the classic, epidemic (acquired immunodeficiency syndrome-associated KS), endemic and post-transplantation associated KS lesions show a similar histopathology characterized by spindle shaped endothelial cells with latent KSHV infection expressing endothelial markers (CD31, CD34, CD36, and EN4), extensive neo-angiogenesis and inflammatory infiltration [21],[38],[39],[40]. We analyzed the skin and lymph node tissue sections of healthy subjects and KS+ patients obtained from ACSR for the presence of COX-2 with anti-COX-2 antibody. Normal healthy control tissue sections (Figure 1A, panel 1) and normal healthy lymph node sections (Figure 1A, panel 5) showed negligible expression of COX-2. In contrast, abundant COX-2 expression was detected in KS skin tissue (Figure 1A, panel 2; Figure S1) and KS lymph node section (Figure 1A, panel 6; Figure S1). Intense, patchy COX-2 expression was detected in KS lymph node sections, especially surrounding neovascular structures (Figure 1A, panels 6 and 7; Figure S1). KS skin tissue and lymph node sections showed distinct nuclear staining for KSHV latency associated LANA-1 (ORF73) protein (Figure 1A, panels 4 and 8; Figure S1). Specificity of COX-2 staining was confirmed by the non-reactivity of isotype control for COX-2 antibody (Figure 1A, panel 12). Cytoplasmic COX-2 staining was observed in KS skin tissue sections, which were also observed in cells showing spindle phenotype (Figure 1A, panels 3 and 11). Strong COX-2 staining was also observed in the lining of neovascular structures in KS patient lymph nodes (Figure 1A, panels 6, 7, 9 and 10). We next assessed the phenotype of spindle cells for endothelial marker CD31 as well as COX-2. CD31 (red) was detected in spindle cells of KS lesions (Figure 1B, panels 1–6) and in KS patient lymph nodes (Figure 1B, panels 7–15). KS tissue sections (skin and lymph nodes) showed many strong CD31-COX-2 double positive cells (Figure 1B, panels 1–15). Many CD31 positive cells in KS skin tissue (Figure 1B, panels 1–6) and cells lining the neovascular structures in KS lymph node sections (Figure 1B, panels 7–15) displayed strong staining for COX-2. COX-2 (green) staining was not just limited to spindle cells present in KS tissues but also to other smaller cells whose morphological appearances suggested that they are most likely macrophages and/or lymphocytes (Figure 1B, panel 3). Strong COX-2 and CD31 co-staining was also observed in the lining of KS lymph node neovascular structures (Figure 1B, panels 7–15).

To define the prevalence of COX-2 up-regulation in KS, COX-2 staining was performed in KS-TMAs as described in Material and Methods. Varying level of COX-2 staining was observed in a variety of tissues from KS patients (Figure S2). Several sections from skin showed negligible staining (Figure 1C, panels 1 and 2) whereas the majority of them showed strong patches (Figure 1C, panels 4 and 5) of COX-2 staining. Very low COX-2 staining was observed in some sections from mouth (Figure 1C, panel 3). Strong COX-2 staining was observed in eye orbit (panel 6), tonsil (panel 7) and mouth (panel 8) and small bowel (panels 9 and 10) sections. Specificity of COX-2 staining was also confirmed by the non-reactivity of isotype control for COX-2 antibody even when tested on the colon cancer tissue (Figure 1C, panel 12) as compared to staining with COX-2 antibody (Figure 1C, panel 11). To further demonstrate that all TMA sections did not show strong or some nonspecific COX-2 staining, we provide some examples of sections with negligible, low and strong staining for COX-2 (Figure S2). A higher number of sections showed immunoreactivity for COX-2 but the levels of staining varied among the sections (Figure S2) suggesting a potential connection between COX-2 and KS. Magnified view of various KS sections described in Figures 1A and 1C are given in Figure S1 which clearly demonstrate COX-2 distribution in KS tumor cells with characteristic spindle phenotype. These results demonstrate that COX-2 is an abundant factor in the majority of KS lesions with a few exceptions (Figure 1C, panels 1–3, Table S1), and thereby suggesting that COX-2 might be playing a key role in KSHV pathogenesis.

### De novo KSHV infection induces COX-2 and m-PGES-1 in endothelial cells

Our previous study demonstrated that de novo infection of endothelial cells with 10 DNA copies/ cell of KSHV up-regulated COX-2 during early time points of infection which was maintained at 2–3 fold even at 72 h PI. Here, we extended this observation using higher KSHV DNA copies per cell (30) for infection of HMVEC-d cells and observed the cells until 5d PI. KSHV ORF73 gene expression as assessesd by qRT-PCR (Figure 1D) as well as by real-time RT-PCR with ORF73 gene specific primers and Taqman probes (data not shown) confirmed the successful infection of these cells. Compared to uninfected cells at all the respective time points, KSHV infection induced about 49, 44, 24, 15, 17, 15, 11, and 12- fold COX-2 expression at 2 h, 4 h, 8 h, 1d, 2d, 3d, 4d and 5d PI, respectively (Figure 1D). In addition, we also observed the concomitant induction of m-PGES-1, an enzyme converting PGH2 to PGE2, with about 13, 12.5, 11.3, 13, 10,11, 9, and 7-fold induction at 2 h, 4 h, 8 h, 1d, 2d, 3d, 4d and 5d PI, respectively (Figure 1D). To determine the percentage of cells expressing latent genes and undergoing spontaneous KSHV lytic replication, IFA was carried out using antibodies against ORF73 (latency marker) and ORF59 (processivity factor and a marker of lytic replication) proteins (Figure S3). Detection of a few lytic cycle positive cells at early time points (2h) of KSHV infection could be due to transient lytic burst in primary endothelial cells [41]. At 5 day PI, about 70–80% of cells stained positive for nuclear punctate pattern of ORF73 (Figure S3, panels 4 and 6) and about 9–12% stained positive for lytic ORF59 at early time (2h) (Figure S3, panels 10 and 12), whereas 15–17% cells displayed lytic cycle activation at later (5d) time point of infection (Figure S3, panels 16 and 18). We also detected a low level (8–10%) of lytic induction at 4d post KSHV infection (Figure S3, panels 13 and 15). The percentage of cells expressing ORF59 at 5d PI was significantly higher than at 4d PI and was reproducible. The spike of lytic burst at 5d PI in HMVEC-d cells could also be due to continued presence of pro-IC rich microenvirnment created by KSHV infection. These infected cells expressed high copy numbers for early lytic cycle switch protein ORF 50 at 2h post KSHV infection (data not shown). We emphasize that all analyses are based on IFA for lytic cycle ORF59 protein and hence, percentage of cells will not be identical for all endothelial cells (HUVEC cells) and might also depend on the number of KSHV DNA copies per cell used for infection.

### COX-2 is expressed in long term KSHV infected endothelial cells

**Figure 2:**
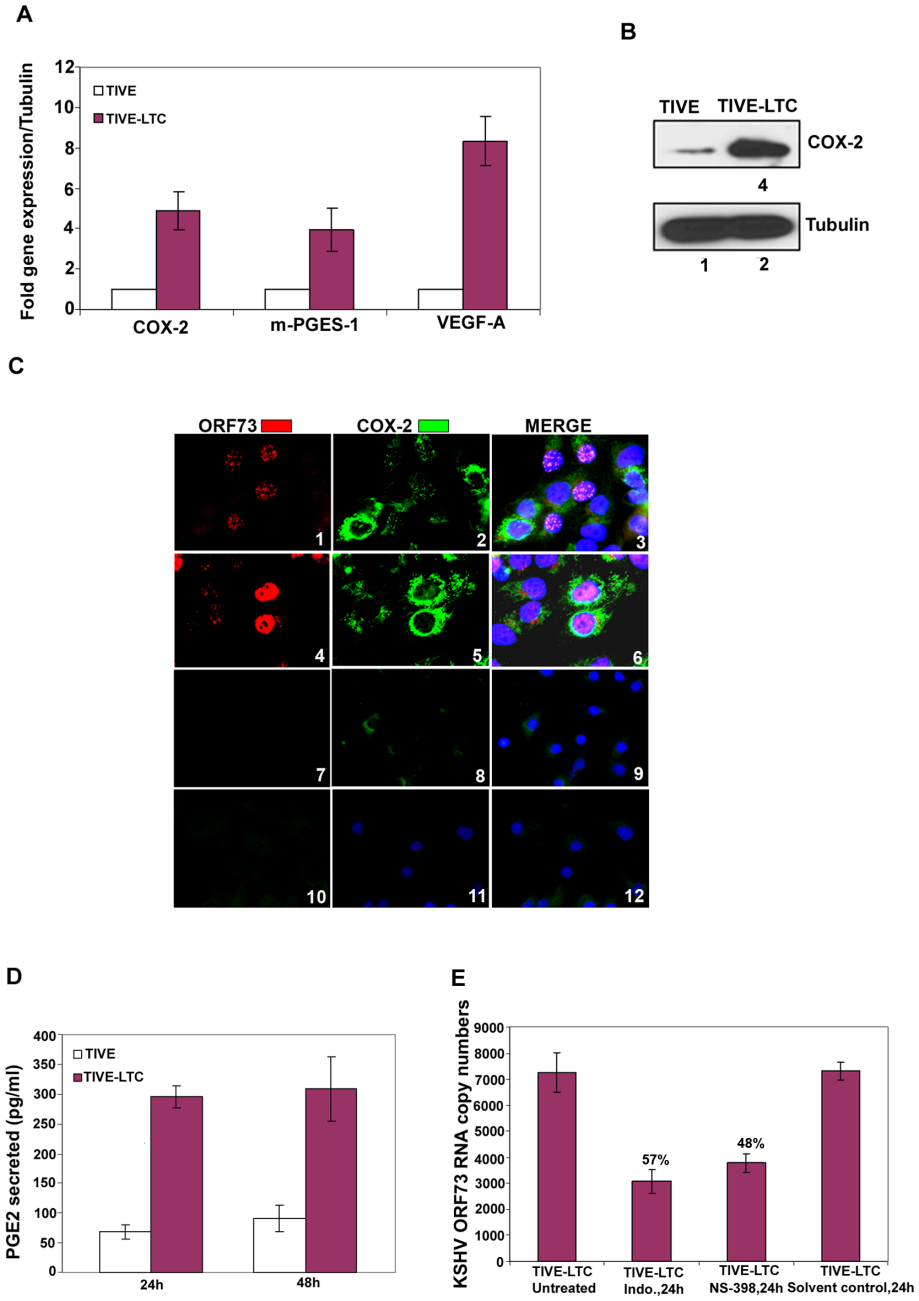
COX-2 in latently infected TIVE-LTC cells. (**A**) **Expression of COX-2, m-PGES-1 and VEGF-A.** TIVE and TIVE-LTC (KSHV) cells were used to prepare total RNA and the expression of host genes like COX-2, m-PGES-1 and VEGF-A (**A**) were analyzed using the ΔΔCt method. Fold induction was calculated by considering expression in TIVE cells as 1-fold. (**B) Protein levels of COX-2.** Lysates from 24h serum starved TIVE and TIVE-LTC cells were Western blotted for COX-2, stripped and immunoblotted for tubulin and a representative blot from three independent experiments is shown. (**C**) **Immunofluoresence analysis of ORF73 and COX-2.** TIVE and TIVE-LTC cells were grown to 80–90% confluence, serum starved for 24 h, fixed, permeabilised and examined with ORF73 (red) and COX-2 (green) specific antibodies. (**D**) **PGE2 secretion.** Conditioned media from 24 h or 48 h serum starved TIVE and TIVE-LTC cells were measured for secreted PGE2 levels using a PGE2 ELISA. (**E**) **Effect of inhibitors on ORF73 gene expression.** TIVE-LTC cells were untreated, solvent control treated or treated with 500 µM Indo or 75 µM NS-398 for 24 h. RNA was isolated and viral transcripts were quantitated. The % inhibition was calculated by considering KSHV-ORF73 gene expression in untreated TIVE-LTC cells as 100%. Each point represents the average ± SD from three independent experiments (**A, D, and E**).

TIVE-LTC cells are endothelial cells in culture with tightly latent KSHV gene expression supporting long-term episomal maintenance which is similar to viral-gene expression in the majority of KS lesion spindle cells [42]. KSHV-positive TIVE-LTC cells expressed very high levels of ORF73 gene expression. Compared to uninfected TIVE cells, TIVE-LTC cells showed increased expression of COX-2 (5-fold), m-PGES-1 (4-fold) and VEGF-A (8-fold) (Figure 2A). Compared to uninfected TIVE cells, KSHV-positive TIVE-LTC cells showed (4-fold) higher levels of COX-2 protein (Figure 2B). Punctate nuclear staining of ORF73 was observed in 50–60% of TIVE-LTC cells (Figure 2C; Panels 1 and 3). Distinct perinuclear COX-2 staining was observed in a majority of the TIVE-LTC cells (Figure 2C; Panels 2 and 5). Besides ORF73 positive cells, the majority of neighboring uninfected cells located in close proximity to the infected cells were also positive for COX-2 (Figure 2C; Panels 2, 3, 5 and 6). Overall, 70–80% of TIVE-LTC cells were positive for COX-2. Detection of COX-2 in uninfected cells could be due to paracrine COX-2 stimulation by the various cytokines and growth factors induced by KSHV. Similarly, COX-2 expressing uninfected cells were also seen in KSHV-infected HMVEC-d monolayer but were distinctly less in number and were in close proximity to the infected cells (data not shown). Similarly stained TIVE cells showed very faint cytoplasmic basal staining for COX-2 in a few cells (Figure 2C; panels 8 and 11) and no staining for viral latent protein ORF73 (Figure 2C; panels 7 and 10). COX-2 staining in TIVE cells (Figure 2C; panels 8, 9, 11 and 12) was not comparable to the strong perinuclear COX-2 staining seen in TIVE-LTC cells (Figure 2C; panels 2, 3, 5 and 6). Compared to uninfected TIVE cells, significantly higher levels of PGE2 (pg/ml) were detected in the supernatants of TIVE-LTC cells (Figure 2D).

### Inhibition of COX-2 reduces KSHV latent ORF73 gene expression in TIVE-LTC cells

Since COX-2 inhibition down-regulated ORF73 gene expression during de novo KSHV infection [26], we next determined the effect of NS-398 and Indo treatment on ORF73 gene expression in TIVE-LTC cells. First, we determined the concentrations of COX inhibitors affecting PGE2 secretion. TIVE-LTC cells pretreated with nontoxic doses of either Indo (500 µM or 250 µM) or NS-398 (50 µM or 75 µM) at 37°C for 1h did not completely inhibit PGE2 secretion (Figure S4). In contrast, by increasing the incubation period with these inhibitors to 8 h and 24 h, we observed a significant reduction (∼80%) in PGE2 secretion (Figure S4). This requirement for a higher dose of inhibitors to block COX-2 function and PGE2 secretion could be due to the continuous loop of COX activation leading to the maintenance of a constant level of PGE2 in latently infected cells.

NS-398 and Indo treatment of TIVE-LTC cells for 24 h down-regulated viral latent (ORF73) gene expression by 48% and 57%, respectively (Figure 2E). Significant detection of COX-2 in KS lesions (Figure 1A, B and C), long term KSHV infected endothelial cells (Figure 2) and in de novo infection of endothelial and fibroblast cells [26], as well as modulation of viral gene expression by COX inhibition (Figures 1 and 2), strongly indicated a role for COX-2/PGE2 in KSHV pathogenesis.

### COX-2 silencing reduces COX-2 gene expression and PGE2 secretion in HMVEC-d cells

Pre-treatment of endothelial cells with chemical nonsteroidal anti-inflammatory drugs (N SAID) like Indo or COX-2 selective inhibitor (COXIB) NS-398 prior to KSHV infection abrogated the secretion of PGE2 [26]. These conventional NSAIDs have been shown to cause serious and significant complications [43]. Though the selective COX-2 inhibitors cause only occasional deleterious effects, they have also been shown to exhibit some COX-2 independent effects such as up-regulation of death receptor 5 (DR5) expression, inhibition of survival signal pathways, and augmentation of apoptosis [43],[44]. To determine the specificity of COX-2 involvement in KSHV pathogenesis and to avoid COX independent effects of chemical inhibitors, we used a COX-2 silencing method.

**Figure 3:**
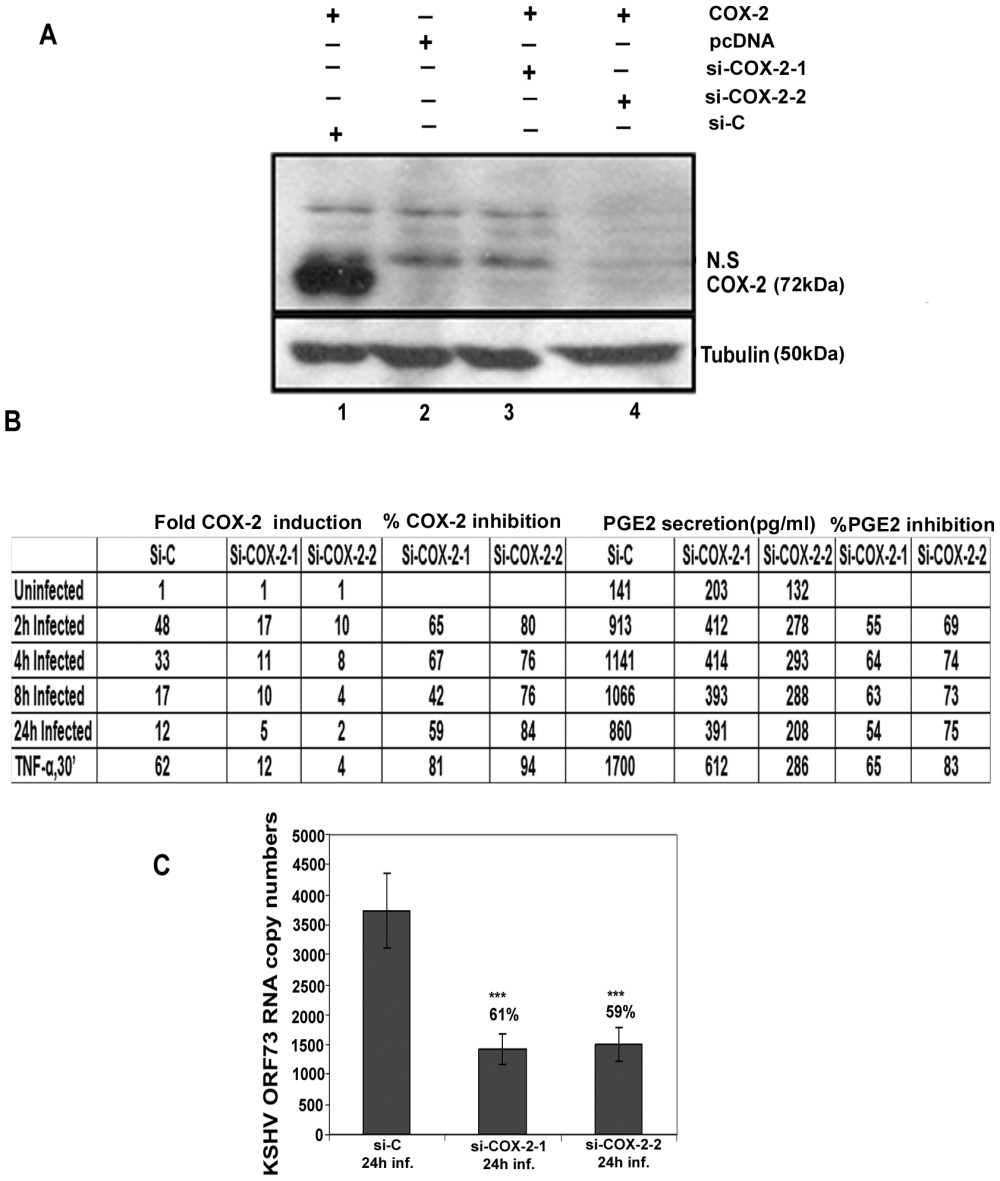
Evaluation of COX-2 siRNA for silencing COX-2. (**A**) Two constructs, si-COX-2 -1 and si-COX-2-2 expressing siRNAs for COX-2 were generated. Lysates from 293T cells co-transfected with COX-2 expression plasmid and si-COX-2-1 (lane 3) or si-COX-2-2 (lane 4) or si-C (lane 1) (36 h) were Western blotted for COX-2, stripped and immunoblotted for tubulin. Lysate prepared from 293T cells transfected with pcDNA alone was used as control (lane 2). N.S =  non-specific band. (**B**) HMVEC-d cells transduced for 48 h with si-C or si-COX-2-1 or si-COX-2-2 were serum starved for 8 h, and infected with KSHV for 2, 4, 8 and 24 h or stimulated with TNFα (20 ng/ml) for 30′. RNA from these cells was analyzed by q-RT-PCR for COX-2 expression and the supernatants were used to quantify PGE2 concentration by ELISA**.** Panel B represents the fold induction of COX-2 gene expression calculated by considering expression in uninfected cells as 1 fold. Similarly, PGE2 levels in quadruplicate samples were measured and values are presented in pg/ml. % inhibition was calculated by considering COX-2 expression or PGE2 release from the infected cells at the respective time of measurement as 100%. Data is from four independent experiments. (**C**) **Effect of COX-2 silencing on KSHV ORF73 gene expression.** HMVEC-d cells were transduced with si-C, si-COX-2-1 and si-COX-2-2 and after 48 h, serum starved for 8 h and infected with 30 DNA copies/ cell of KSHV for 24 h. RNA was isolated and treated with DNase I, and 250 ng of DNase-treated RNA was subjected to real-time RT-PCR with KSHV ORF73 gene-specific primers and TaqMan probes. The relative copy numbers of viral transcripts were calculated using a standard graph generated by using known concentrations of DNase-1 treated, in vitro-transcribed ORF73 transcripts in real-time RT-PCR and normalized with GAPDH. Each reaction was done in duplicate, and each point represents the average ± SD from three independent experiments. The % inhibition was calculated by considering KSHV-ORF73 gene expression in 24 h infected si-C HMVEC-d cells as 100%.

293T cells were co-transfected with COX-2 expression plasmid and si-COX-2-1, si-COX-2-2 and si-Control (si-C) plasmids. Transfection with pcDNA was used as a control (Figure 3A, lane 2). Western blots for COX-2 confirmed the silencing of COX-2 by si-COX-2 (Figure 3A, lanes 3 and 4) compared to si-C (Figure 3A, lane 1), and tubulin was utilized as a loading control. Co-transfection of 1 µg COX-2 expression plasmid and si-C showed 10-fold induction of COX-2 protein (Figure 3A, lane1). Transfection with 1 µg of either si-COX-2-1 or si-COX-2-2 along with COX-2 expression plasmid showed 85% and 90% reduction in COX-2 protein levels, respectively (Figure 3A, lanes 3 and 4). These results clearly implied that COX-2 silencing by these sequences was effective. We used both the plasmids to generate si-COX-2 lentiviruses which were used throughout this study. The lentivirus preparations were quantified for their titer and 30 DNA copies/ cell of all three lentiviruses [si-C, si-COX-2-1 and si-COX-2-2] were used for transduction in HMVEC-d cells. We observed very high transduction efficiency (>90% of HMVEC-d cells expressing GFP) by fluorescence microscopy.

### Silencing of COX-2 reduces KSHV infection induced COX-2 gene expression and PGE2 secretion in HMVEC-d cells

Effect of COX-2 silencing in HMVEC-d cells was determined by infecting serum starved (8 h) si-C, si-COX-2-1 or si-COX-2-2 transduced cells for 2 h, 4 h, 8 h, and 24 h. These cells were treated with TNF-α for 30′ to serve as a positive control for COX-2 induction. Compared to uninfected si-C cells, KSHV infected si-C-HMVEC-d cells showed high COX-2 gene expression (Figure 3B). In contrast, KSHV infected si-COX-2-1 or si-COX-2-2 -HMVEC-d cells showed significantly reduced COX-2 expression (Figure 3B). Overall, si-COX-2-1 or si-COX-2-2 -HMVEC-d cells showed 82% and 93% reduction in COX-2 expression, respectively (Figure 3B).

We next assessed the functional consequences of COX-2 silencing by quantifying the secreted PGE2 levels in the supernatant of KSHV infected lentivirus transduced HMVEC-d cells (Figure 3B). In si-C-HMVEC-d cells, PGE2 secretion levels dramatically increased upon KSHV infection. Though there was induction in PGE2 levels in si-COX-2 transduced cells upon KSHV infection, this induction was lower than in si-C-HMVEC-d cells (Figure 3B). Similarly, TNF-α induced PGE2 secretion was reduced drastically in si-COX-2- HMVEC-d cells (Figure 3B) suggesting that COX-2 silencing could effectively abrogate KSHV infection induced PGE2 secretion in endothelial cells. COX-2 silencing did not change COX-1 expression (data not shown) further validating the specificity of the knock-down procedure.

### Silencing of COX-2 reduces KSHV latent ORF73 gene expression in HMVEC-d cells

We also assessed the consequence of COX-2 silencing on KSHV latent gene expression (Figure 3C). After 24 h KSHV infection, we observed about 61% and 59% reduction in ORF73 gene expression in si-COX-2-1 and si-COX-2-2 -HMVEC-d cells, respectively (Figure 3C). These results supported our earlier findings in HFF cells with chemical inhibitors [26] and demonstrated that COX-2 silencing effectively reduced KSHV latent gene expression.

### De novo infection of HMVEC-d cells induces secretion of pro-inflammatory cytokines

**Figure 4:**
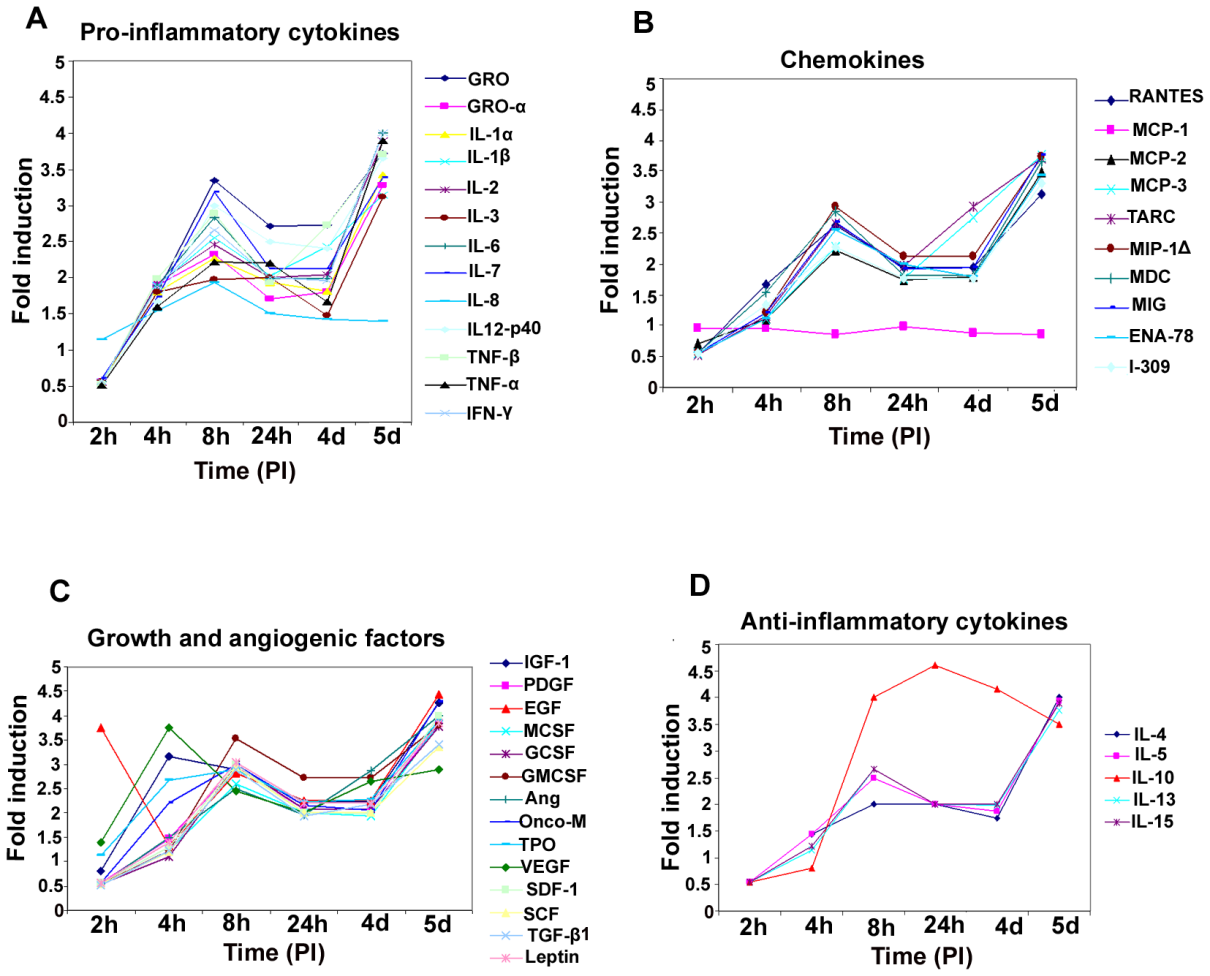
Induction of pro-inflammatory cytokines, chemokines, growth, angiogenic factors, and anti-inflammatory cytokines in HMVEC-d cells by KSHV infection. Densitometric analysis of cytokine array blots was carried out to determine the differences in the release of cytokines from serum-starved, uninfected HMVEC-d cells and cells infected with KSHV for 2 h, 4 h, 8 h, 24 h, 4 days and 5 days. The values were normalized to identical background levels using the Ray-Biotech Array 3.1 analysis tools. The increase in cytokine levels was calculated by dividing the respective values obtained from infected-cell supernatants with the values obtained from uninfected-cell supernatants and cytokines showing significant changes with respect to uninfected cells are represented in a line graph format. (**A**) Pro-inflammatory cytokines; (**B**) chemokines; (**C**) growth and angiogenic factors; (**D**) anti-inflammatory cytokines.

In our earlier studies of oligonucleotide array analysis of KSHV-infected HMVEC-d and HFF cells at 2 and 4 h PI, we observed the reprogramming of host transcriptional machinery regulating a variety of cellular processes, including apoptosis, cell cycle regulation, signaling, inflammatory response and angiogenesis [25]. Since COX-2 has also been shown to regulate the majority of these factors, we next analyzed the role of KSHV-induced COX-2 in the modulation of these factors. Conditioned media (no serum) collected from KSHV-infected HMVEC-d cells at 2 h, 4 h, 8 h, 24 h, 4 days and 5 days PI were used to study the cytokine profile (Figure 4). Induction of cytokines was compared to the released cytokine levels in the uninfected cell supernatant at respective time points. Compared to uninfected HMVEC-d cells, KSHV infection triggered an appreciable (1.5-2) fold induction in the secretion of pro-ICs, such as growth regulated oncogene (GRO), GROα, IL-1α, IL-1β, ILs- (2,3, 6, 7, and 12-p40), TNF-α, TNF-β and IFN-γ at 4 h PI. 1.5 to 2-fold induction in these cytokine levels further up-regulated to 3 –3.5 -fold by 8 h, decreased to 2–2.5-fold by 24 h, 1.5–2 -fold at the 4d, and enhanced dramatically to 4–4.5 -fold at 5d PI (Figure 4, Table S3). Since, we did not observe an increase in these cytokines released at 2h PI, we used 4, 8 and 24 h PI time points throughout this study. Among all these cytokines, IL-8 levels did not increase at 5d PI. The drastic increase in the cytokine levels observed at 5d PI might be due to the spontaneous induction of KSHV lytic cycle replication observed in about 15–17% of these infected cells (Figure S3).

### De novo infection of HMVEC-d cells induces secretion of chemokines

Compared to uninfected cells, KSHV infection induced the secretion of chemotactic cytokines (chemokines) that mediate leukocyte recruitment to sites of inflammation, fibrosis, and malignancy such as RANTES, macrophage chemoattractant protein-2 (MCP-2), MCP-3, thymus and activation-regulated chemokine (TARC), macrophage inflammatory protein (MIP-1Δ), macrophage derived chemokine (MDC), monokine induced by IFN-Gamma (MIG), epithelial neutrophil-activating peptide (ENA-78), and inflammatory cytokine 309 (I-309) (Figure 4B, Table S3). Among these chemokines, MCP-1 was the only one that was not up-regulated at all time points tested as the uninfected cells always showed some level of MCP-1 secretion in the culture supernatants.

### De novo infection of HMVEC-d cells by KSHV induces secretion of multiple growth and angiogenic factors

KSHV infection stimulated the secretion of growth factors and angiogenic factors such as insulin-like growth factor-1 (IGF-1), platelet derived growth factor-BB (PDGF-BB), macrophage colony stimulating factor (M-CSF), granulocyte colony-stimulating factor (G-CSF), GM-CSF, angiogenin (Ang), oncostatin-M (Onco-M), TPO (thrombopoietin), VEGF, stromal cell-derived factor-1 (SDF-1), SCF (stem cell factor), TGF-β1 and leptin (Figure 4C, Table S3). EGF (epidermal growth factor) was very highly up-regulated at the early time points of KSHV infection with about ∼4 fold induction at 2 h PI, which decreased to 1.5-fold by 4 h PI, increased at 8h to ∼3- fold before decreasing to 2- fold by 24h and 4 days, and finally increased at 5 days PI (Figure 4C, Table S3). Endogenous levels of EGF were high as the supernatants obtained from uninfected cells also showed higher levels of EGF secreted (data not shown).

### De novo infection of HMVEC-d cells induces secretion of anti-inflammatory cytokines

Compared to uninfected HMVEC-d cells, KSHV infection enhanced the secretion of anti-inflammatory cytokines, such as ILs (−4, −5,−10, −13 and −15) with 1.5 to 2.5-fold at 4 and 8h PI which decreased to 2-fold at 4d and was up-regulated at 5d PI (Figure 4D, Table S3). IL-10 levels were higher than all other anti-ICs with ∼4 fold from 8h PI to 5d PI (Figure 4D, Table S3).

### Specificity of KSHV induced cytokine secretion

To evaluate the specificity of KSHV infection induced cytokine secretion, virus was pre-incubated with 100 µg of heparin/ml which has been shown to block about 80% of virus binding and entry into the various target cells [26],[35]. Conditioned medium from serum starved HMVEC-d cells infected for 96h (Figure S5A, panel 1) showed a significant increase in the levels of various cytokines and inflammatory molecules, which were greatly reduced by pretreatment of the virus with heparin (Figure S5A, panel 2). Representative data from one time point of infection shows that there was complete inhibition of SDF-1, SCF, TGF-β and TARC with 60–70% inhibition of GM-CSF, GRO, GRO-α, ILs (−2,−3, −4,−5, and −10), MDC, MIG, MIP-1Δ, RANTES, IGF-1, angiogenin, oncostatin-M, and TPO and 30–40% inhibition of VEGF, PDGF-BB, IL-7, IL-1β, and IL-8. There was no detectable inhibition in secretion of MCP-1 and EGF (Figure S5A, panel 2) which might be due to their high endogenous levels of secretion even in the uninfected cell culture supernatant. These results demonstrated that the vast majority of the observed cytokine induction (Figure 4, A to D) was due to KSHV infection and not due to LPS or contaminating host cell factors in the virus preparations.

### KSHV induced COX-2 regulates the expression of a number of KSHV induced cytokines

**Figure 5:**
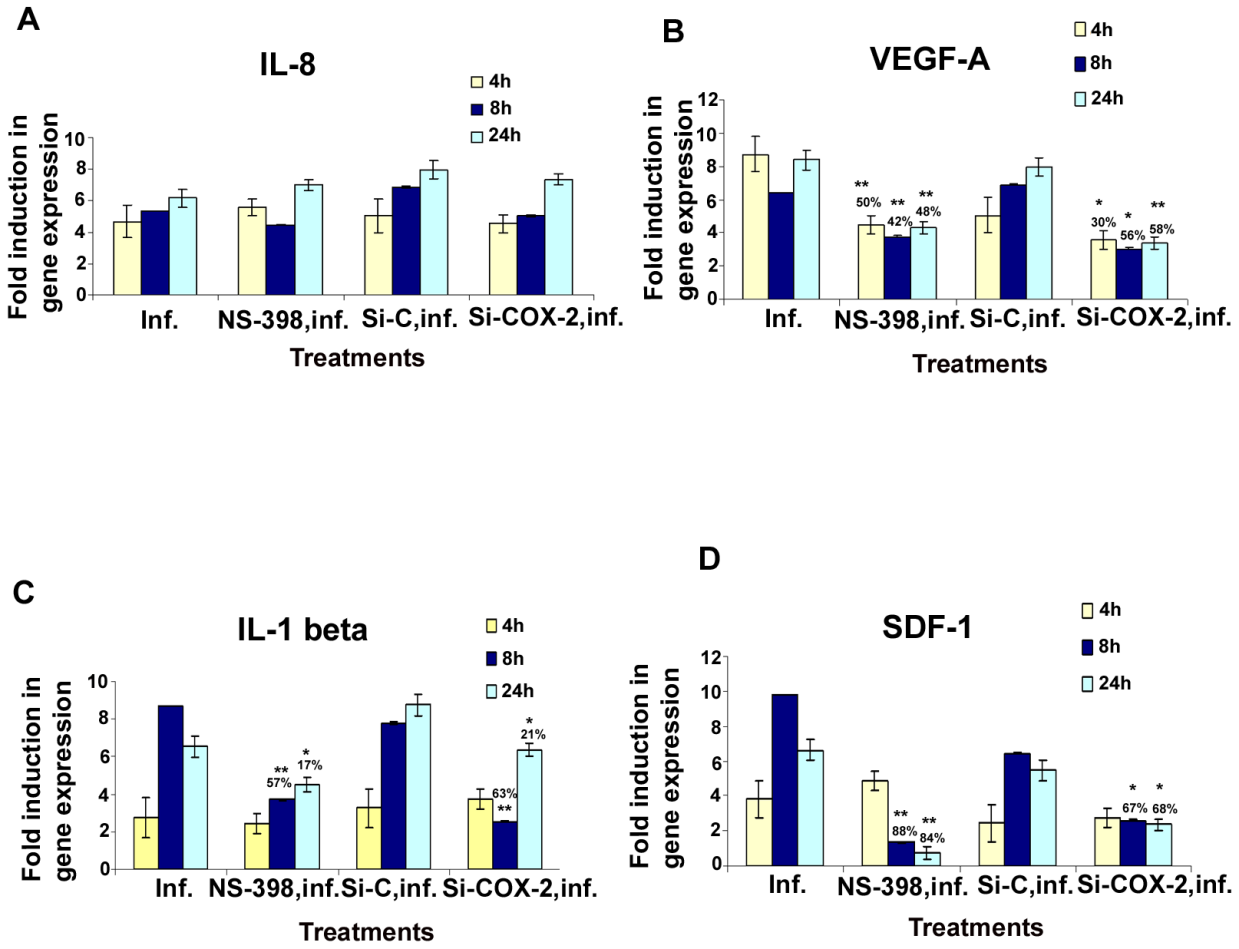
Effect of NS-398 or COX-2 silencing on KSHV infection induced cytokine gene expression. Histograms depict the fold induction in gene expression of KSHV infected, or NS-398 pretreated for 1 h and then infected with KSHV, or si-COX-2-2-HMVEC-d/si-C-HMVEC-d cells infected with 30 DNA copies/ cell of KSHV for 4 h, 8 h, and 24 h. IL-8 (**A**), VEGF-A (**B**), IL-1β (**C**), SDF-1 (**D**). The % inhibition was calculated by considering cytokine gene expression in the infected cells at the respective time of measurement as 100%. Each reaction was done in quadruplicate, and each bar represents the average ± SD of four independent experiments. *,**, ***-statistically significant at p<0.01, p<0.005 and p<0.001 respectively.

To understand the role of KSHV induced COX-2 in cytokine secretion detected in infected HMVEC-d cells, we used COX-2 inhibitors in conjunction with COX-2 silencing methods and examined cytokine gene expression. We prepared cDNA from serum starved (8h) cells either infected (4 h, 8 h, 24 h) or pretreated with either NS-398 (50 µM) or Indo (500 µM) (data not shown), and then infected with KSHV for 4 h, 8 h, and 24 h. Similarly, cDNA was prepared from serum starved (8 h) cells transduced with si-C, si-COX-2-1 or si-COX-2-2 and then uninfected or infected with KSHV for 4 h, 8 h and 24 h. cDNA was used for q-RT-PCR to quantitate the fold expression of specific cytokines, selected based upon our observations from Figure 4, such as IL-8, VEGF-A, GRO, VEGF-C, IL-1β, GM-CSF, RANTES, and SDF-1, normalized to the expression of endogenous HPRT and tubulin genes. Analyses of the results showed time dependent patterns of inhibition of gene expression by both methods of COX-2 inhibition (Figures 5 and S6).

In pattern one, KSHV infection induced nearly 5, 5.3, and 6.1-fold expression of IL-8 gene at 4 h, 8 h and 24 h PI, respectively compared to uninfected HMVEC-d cells, which were unaffected by pretreatment of cells with NS-398 for 1 h or by si-COX-2-2. These results suggested that IL-8 gene expression was not directly regulated by KSHV induced COX-2 (Figure 5A). In pattern two, in contrast, both COX-2 inhibitor treatment and COX-2 knockdown reduced KSHV induced VEGF-A and VEGF-C gene expression significantly at both early and late time points (Figures 5B and S6A), thus suggesting a role for KSHV induced COX-2 in the regulation of VEGF-A and -C at all time points of infection. In pattern three, a significant reduction of IL-1β and GM-CSF gene expression by COX-2 inhibitor treatment and COX-2 knockdown was observed at 4 and 8 h PI, and was moderately less at 24 h PI (Figures 5C and S6B). These results suggested a role for COX-2 in KSHV induced IL-1β gene expression at an early time point of infection. In pattern four, the expression of KSHV induced GRO, RANTES and SDF-1 genes were not significantly affected by NS-398 and si-COX-2 at 4 h PI, while significant reductions were observed at 8 and 24 h PI (Figures S6C, S6D, and 5D). This suggested that COX-2 plays a role in the regulation of these genes at later time points.

Overall, inhibition of cytokine gene expression by COX-2 inhibitor treatment and COX-2 knockdown were comparable. Both methods inhibited gene expression of VEGF-A, VEGF-C (angiogenic molecules), GRO (cytokine with inflammatory and growth-regulatory properties), RANTES (cytokine regulating T cell response) and SDF-1(a ligand for the chemokine receptor CXCR4) even at 24 h PI. Inhibition of IL-1β gene expression at early time points is not due to the inactivation of COX-2 inhibitor or less inactivation of COX-2 by the silencing method but demonstrates the specificity of the observed results. Absence of 100% reduction in the expression of the examined cytokine genes by both methods could be due to the inability to inactivate or deplete COX-2 completely, paracrine effects of the released additional factors and/or additional factors besides COX-2 in the regulation of these genes in KSHV infected cells.

To examine the role of COX-2 in the secretion of various cytokines during KSHV infection, we analyzed the cytokines from COX-2 inhibited cells. Data obtained from COX-2 inhibitor pretreatment followed by infection (4 h, 8 h, 24 h) or cells silenced for COX-2 and then infected for different time points (4 h, 8 h, 24 h) is presented as fold reduction compared to signals obtained from untreated KSHV infected cells, and KSHV infected si-C-HMVEC-d at respective time points (Table S4). Results shown in Table S4 can be divided into three groups. Group 1 includes cytokines inhibited by both kinds of COX-2 inhibition (chemical as well as silencing). Group 2 includes the cytokines inhibited by chemical inhibitor (NS-398) treatment alone but not reduced by COX-2 knock-down. Group 3 includes the cytokines up-regulated by COX-2 inhibition.

Group 1 cytokines are specifically dependent upon COX-2 which includes: pro-ICs like IL-1 (α and β), ILs (−2, −3, −p40 and −16) TNFα, IFNγ, LIGHT; chemokines including RANTES, MCP-2, MCP-3, TARC, MIP-1Δ, ENA-78, I-309, MIF, GCP-2, MIP-3-α, Eotaxin, Eotaxin-2, Eotaxin-3, IP-10, NAP-2, CK-β8-1; growth and angiogenic factors including PDGF-BB, MCSF, G-CSF, GMCSF, angiogenin, oncostatin M, thrombopoeitin, VEGF, SDF-1, SCF, TGF-β1, Leptin, FGFs (−4, −6, −7, −9), Flt3-ligand, Fractalkine, IGFBPs (−2, −3, −4), BDNF, PIGF, HGF (hepatocyte growth factor), Osteoprotegerin, NT-3, NT-4; and anti-inflammatory cytokines like IL-4, IL-13, and IL-15. In all these cytokines reported, although the fold reduction between the inhibitor treatment and COX-2 silencing were not identical, they were comparable. Overall, a profound reduction was observed in the levels of ILs (1β, −2, −3,−4, −13, −15 −16, −p40), IFNγ, MCPs (−2, −3), TARC, MIP-1Δ, ENA-78, I-309, GCP-2, Eotaxin, Eotaxin-3, PDGF-BB, G-CSF, angiogenin, Oncostatin M, TPO, VEGF, SDF-1, SCF, TGF-β1, Leptin, FGFs (−4, −6, −7), Flt3-ligand, Fractalkine, IGFBP-4, BDNF, PIGF, Osteoprotegerin, NTs (−3, −4) (Table S4). Group 2 cytokines including pro-ICs (GRO, GRO-α, IL-6, IL-7, IL-8, TNFβ), chemokines (MCP-1, MDC, MIG), growth and angiogenic factors (IGF-1 and IGFBP-1), as well as anti-inflammatory cytokines (IL-5) were only inhibited by chemical inhibitor (NS-398) treatment and not by COX-2 knock-down (Table S4). These results indicate some COX-2 independent effects of chemical inhibitors. Growth factor EGF, anti-inflammatory cytokine IL-10, regulators of MMP activity like TIMP-1 and TIMP-2 were up-regulated by COX-2 inhibition (group 3) (Table S4).

### KSHV induced COX-2 plays a role in KSHV induced VEGF-A and VEGF-C secretion

**Figure 6:**
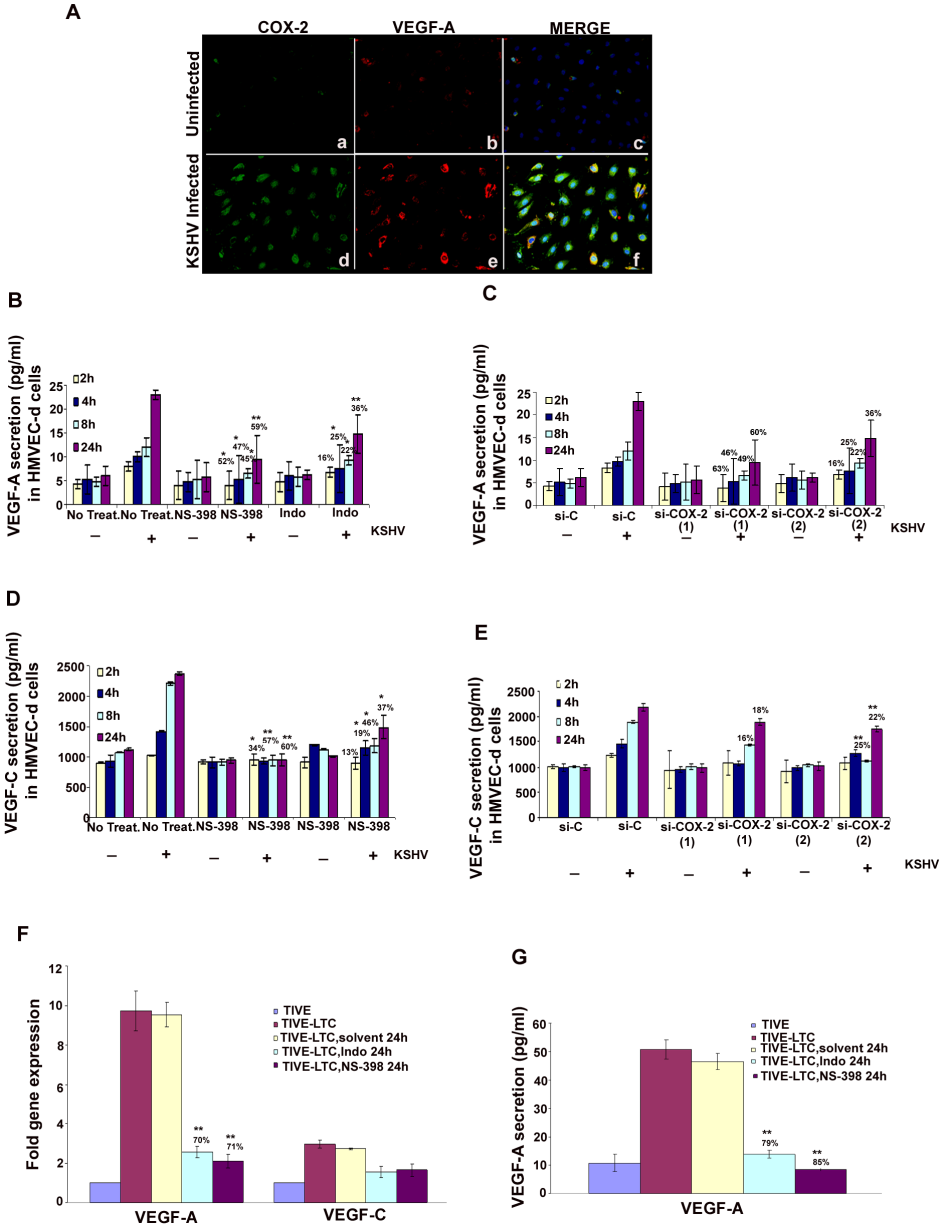
Role of COX-2 in KSHV-induced VEGF-A and C. (**A**) Immunofluorescence analysis of COX-2 and VEGF-A expression in uninfected HMVEC-d and KSHV infected cells (24 h PI) using COX-2 and VEGF-A specific antibodies. (**B-E**) Conditioned media from serum-starved uninfected HMVEC-d cells, KSHV infected cells, cells incubated with COX-2 specific inhibitor NS-398 or non-COX isotype selective inhibitor Indo and from cells silenced for COX-2 or lamin (control) and then infected were collected and analyzed by ELISA for the released VEGF-A (**B, C**) and VEGF-C (**D, E**) proteins. Each reaction was done in quadruplicate, and each point represents the average ± SD from four independent experiments. Data was normalized to 1 mg/ml total protein concentration in the supernatant. Proteins secreted from uninfected cells were considered 1-fold. % inhibition of VEGF-A and VEGF-C secretion was calculated by considering the release from the infected cells at the respective time of measurement as 100%. (**F**) VEGF-A and VEGF-C gene expression as measured by q-RT-PCR with cDNA prepared from serum starved (24 h) TIVE-LTC cells untreated or treated with either 500 µM Indo or 75 µM NS-398 for 24 h. Result shows the mean ± S.D of three independent experiments. % inhibition in gene expression upon inhibitor treatment was calculated using gene expression in untreated TIVE-LTC cells as 100%. (**G**) Conditioned media from serum-starved (24 h) TIVE-LTC cells untreated or treated with either 500 µM Indo or 75 µM NS-398 for 24 h were collected and analyzed by ELISA for the released VEGF-A. Each reaction was done in quadruplicate, and each point represents the average ± SD from four independent experiments. Data was normalized to 1 mg/ml total protein concentration in the supernatant. Proteins secreted from untreated TIVE-LTC cells were considered 100%.*, **, ***-statistically significant at p<0.01, p<0.005 and p<0.001 respectively.

Over-expression of COX-2 is known to correlate with the aggressive and invasive potential of tumor cells by several mechanisms [32]. One of the mechanisms modulated by COX-2 during carcinogenesis is angiogenesis, presumably through increased production of the most potent and extensively studied pro-angiogenic factor, VEGF [45]. Here, we examined the role of KSHV induced COX-2 in the secretion of two angiogenic factors, VEGF-A and VEGF-C. VEGF-A is a dimeric glycoprotein with structural homology to PDGF, and is known to have several variants. We used an anti-human VEGF-A mouse monoclonal antibody that detects all isoforms, particularly the most commonly expressed 189, 165 and 121 amino acid splice variants. When examined by IFA, uninfected HMVEC-d cells showed a very low level of expression of COX-2 and VEGF-A (Figure 6A, panels a and b). In contrast, at 24 h PI, several cells were positive for cytoplasmic staining of COX-2 and VEGF-A (Figure 6A, panels d and e). Co-expression of VEGF-A and COX-2 was observed in 40–50% of infected cells (Figure 6A, panel f), which demonstrated that KSHV infection of primary endothelial cells up-regulated expression of both VEGF-A and COX-2 simultaneously. Uninfected cells in close proximity to the infected cells also showed expression of COX-2 and VEGF-A (data not shown).

Basal levels of VEGF-A (Figure 6B and 6C) and VEGF-C (Figure 6D and 6E) secretion in uninfected non-transduced and si-C, si-COX-2-1 or si-COX-2-2 transduced HMVEC-d were similar. Pretreatment of HMVEC-d cells with either NS-398 or Indo did not have any non-specific inhibition on the basal levels of VEGF-C secretion (Figure 6D). KSHV infection of serum starved HMVEC-d cells induced 1029, 1420, 2211, and 2371 pg/ml of VEGF-C secretion at 2 h, 4 h, 8 h, and 24 h, respectively. (Figure 6D), while 1239, 1457, 1897, and 2200 pg/ml of VEGF-C secretion in serum starved si-C-HMVEC-d cells was observed at 2 h, 4 h, 8 h, and 24 h, respectively (Figure 6E). Treatment of cells with either NS-398 or Indo prior to KSHV infection reduced VEGF-A and VEGF-C secretion at all the time points tested (Figures 6B and 6D). Analyses showed that NS-398 pretreatment inhibited VEGF-A and VEGF-C secretion which was higher than inhibition by Indo treatment (Figures 6B and 6D). COX-2 silencing also inhibited VEGF-A and VEGF-C secretion but to a lesser extent than chemical inhibitor treatment (Figures 6B-6E). The incomplete inhibition of VEGF secretion by COX-2 chemical inhibitors suggested that besides COX-2, other factors induced by KSHV infection might also be playing a role in VEGF release from infected cells.

### COX-2 regulates VEGF-A gene expression and secretion in latently infected endothelial cells

To evaluate the role of COX-2 in regulating angiogenesis in latently infected cells, we checked the expression of VEGF-A and VEGF-C (Figure 6F) in TIVE-LTC cells untreated or treated with either 500 µM Indo or 75 µM NS-398 for 24 h. Compared to TIVE cells, TIVE-LTC cells showed 9.8- and 2.4 fold higher VEGF-A and -C gene expression, respectively (Figure 6F). Pretreatment of TIVE-LTC cells with either Indo. or NS-398 significantly inhibited the expression of VEGF-A, clearly demonstrating the role of COX-2 in regulating VEGF-A gene expression in latently infected cells (Figure 6F). Similar to VEGF-A gene expression, TIVE-LTC cells secreted appreciably high levels of VEGF-A (54 pg/ml) as compared to TIVE cells and this secretion was effectively reduced upon treatment with COX inhibitors (Figure 6G). VEGF-C secretion from TIVE (1800 pg/ml) and TIVE-LTC (2200 pg/ml) cells was comparable.

### KSHV induced COX-2 plays a role in KSHV infection induced capillary tube formation of normal endothelial cells

One important biological effect of PGE2, VEGF, and bFGF secretion is the induction of endothelial cell tube formation. Many chemokines are also recognized as important mediators of endothelial cell migration and tubular organization. For a comprehensive understanding of the role of KSHV induced COX-2 in VEGF-related angiogenesis of infected endothelial cells, conditioned media obtained either from serum starved (8 h) HMVEC-d cells infected with KSHV (24 h), or cells pretreated with COX inhibitors (Indo or NS-398) and then uninfected or infected with KSHV (24h) were tested for their ability to induce tube formation. Representative pictures are shown in Figure S7A. HMVEC-d cells spontaneously organized into a primitive vascular network even in the presence of EBM-2 alone (no serum) (Figure S7A, panel a). This network was still observed with supernatants from cells treated for 24h with COX-2 inhibitors which suggested that inhibitor treatment did not have any adverse effect on the secreted factors in the uninfected cells (Figure S7A, panels b and c). In contrast, highly organized enhanced capillary tube formations with strong branching networks were observed in endothelial cells incubated with conditioned medium from cells infected with KSHV for 24h (Figure S7A, panel d). When supernatants from cells pretreated with COX inhibitors and then infected with KSHV (24h) were used, we observed significant inhibition of tube formation (Figure S7A, panels e and f). Higher inhibition was observed with NS-398 pretreated infected cell supernatant with complete impairment and disintegration of tube formation (Figure S7A, panel f) which is in contrast to the strong, well communicating tubes observed with media after 24h KSHV infection alone (Figure S7A, panel d). These results suggested a direct role for KSHV infection induced COX-2 in the induction of factors mediating endothelial cell capillary tube formation.

When suramin, which possesses anti-angiogenic properties, was used as a negative control, cells failed to adhere to each other and remained either as single cells or clumps of cells with no tube formation (Figure S7A, panel g). In contrast, conditioned medium from cells cultured in the presence of medium with growth factors (EGM-2) showed intact and organized endothelial lattice formation comparable to the network formed in the KSHV infected culture supernatant (Figure S7A, panels h and d). This suggested a role for GFs in tube formation. Supernatant obtained from solvent treated and then KSHV infected cells showed a strong intact tube network that was comparable to the one observed in the presence of cells infected with KSHV for 24h (Figure S7A, panels d and i), thus ruling out the possibility of non-specific inhibition by solvent control on tube formation of endothelial cells. Similar results for tube formation were observed with HUVEC cells (data not shown).

**Figure 7:**
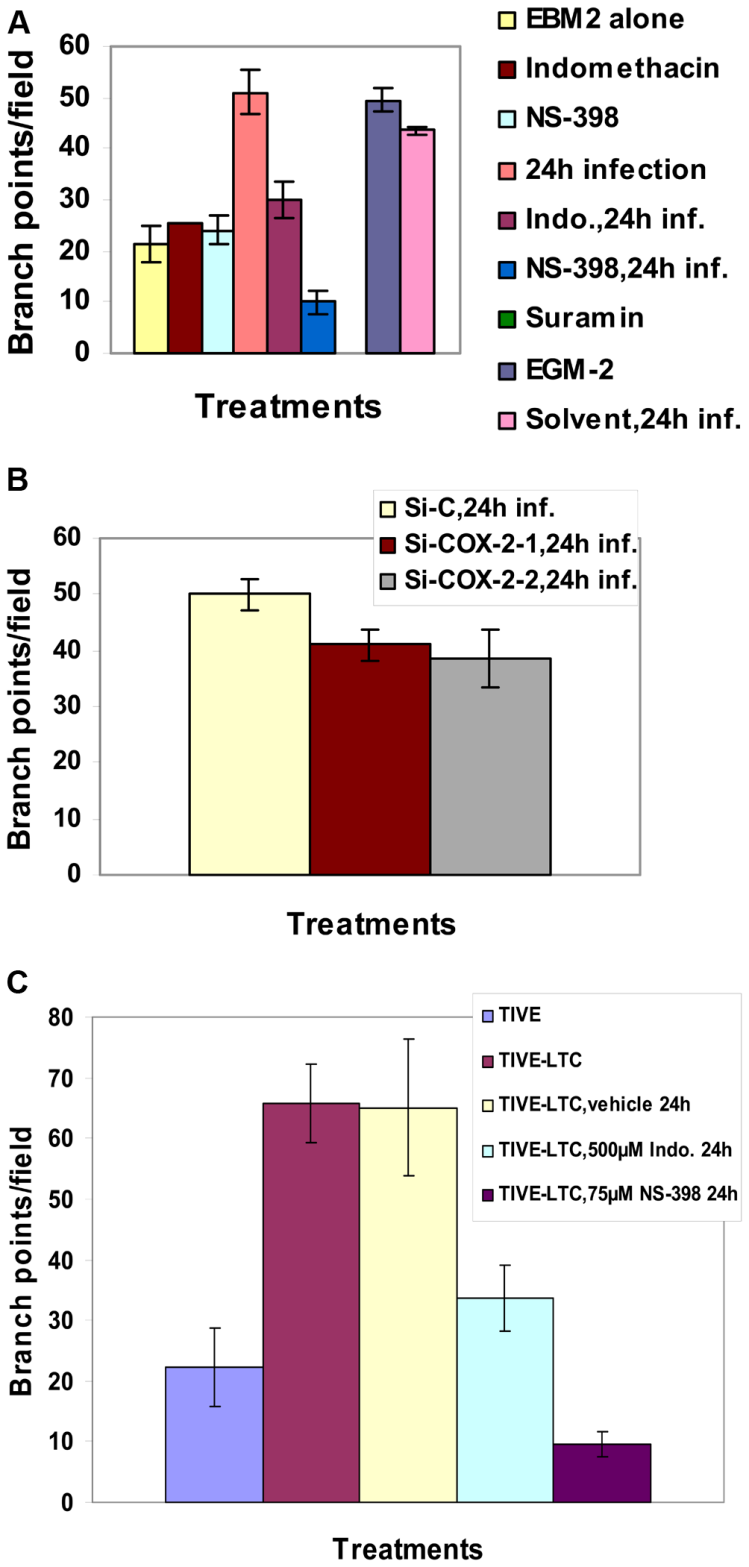
Effect of COX-2 inhibition on KSHV infection-induced capillary tube formation in normal HMVEC-d cells. (**A and B**) Quantitative representation for branch points/field in the presence of supernatants obtained from HMVEC-d cells treated with COX-2 inhibitors or solvent control, or cells pretreated with COX-2 inhibitors and then infected (24 h) or cells silenced for COX-2 and then infected for 24h. Percent inhibition was calculated by considering the branch points/field observed in the presence of KSHV infected supernatant or the supernatant obtained from infected si-C-HMVEC-d as 100%.***-statistically significant at p<0.001. (**C**) Quantitative representation for branch points/field in the presence of conditioned medium obtained from 24 h serum starved TIVE cells and COX-2 inhibitors or solvent pretreated or untreated TIVE-LTC cells. Percent inhibition was calculated by considering the branch points observed in the absence of COX inhibitor treatment in TIVE-LTC cells as 100%. Fold increase in the branch points/field was calculated considering branch points/field in presence of TIVE cell supernatant as 1 fold.

The angiogenic index can be measured either by taking the sum of all the nodes (connection between various tubes on the matrigel) between the tubes formed on the matrigel or the length and width of tube formation between nodes. Results represented in Figure 7A show branch points/field, a measure of connections among cells. Supernatants from KSHV infected cells induced the number of branch points by about 2.5-fold as compared to EBM-2 only. Diffused nodes with incomplete branches were not counted. Branch points per field in the supernatants obtained from KSHV infected cells versus cells in the presence of EGM-2 were similar. Compared to supernatants from KSHV infected cells, supernatants from Indo or NS-398 pretreated and infected cells inhibited node formation by 45% and 80%, respectively (Figure 7A). Supernatants from cells pretreated with the solvent control before KSHV infection for 24h did not show any inhibition in node formation and was comparable to the branch points formed in the presence of EGM-2 or KSHV infection (Figure 7A).

A similar experiment was done for si-C, si-COX-2-1 and si-COX-2-2 transduced HMVEC-d (Figure S7B) and HUVEC cells. Transduction with si-C, si-COX-2-1 or si-COX-2-2 did not have any non-specific effects on the secretion of factors involved in angiogenesis (Figure S7B, panels a, b, and c). Tube formation in medium obtained from si-C transduced cells (Figure S7B, panel a) was comparable to the results from non-transduced uninfected cells (Figure S7A, panel a). Tube formation in the presence of medium obtained from infected si-COX-2-1 (Figure S7B, panels e and f) or si-COX-2-2 transduced cells (Figure S7B, panels h and i) was diminished, and nodes were diffuse compared to supernatants from infected si-C-HMVEC-d cells (Figure S7B, panels d and g). Consistent with the COX-2 inhibitor data, silencing by either si-COX-2-1 or si-COX-2-2 in endothelial cells prior to KSHV infection also reduced the ability of supernatants to induce node formation by approximately 22% and 24%, respectively, compared to si-C-HMVEC-d cells infected for 24h (Figure 7B). This inhibition was less pronounced when compared to the huge reduction (80%) observed with the supernatants in the presence of NS-398. Similar results for tube formation were observed with HUVEC cells (data not shown) implying that COX-2 plays an important role in regulating the angiogenic phenotype of KSHV infected endothelial cells.

### COX-2 regulates angiogenesis and capillary tube formation in latently infected endothelial cells

As TIVE-LTC cells showed high VEGF-A gene expression and secretion, to further assess the biological role of secreted angiogenic factors, an endothelial cell tube formation assay was performed (Figure S8). HMVEC-d cells were seeded on a Matrigel-coated 96-well plate with conditioned medium obtained from 24h serum starved TIVE (Figure S8, panels 1–4), or TIVE-LTC cells (Figure S8, panels 5–8). After 16h of incubation with conditioned media, plates were examined for capillary-like tubular structures as described before. HMVEC-d cells spontaneously organized into a primitive vascular network even in the presence of medium obtained from TIVE cells (Figure S8, panels 1–4). Highly organized and intricate capillary tube formations with strong branching networks were observed in cells incubated with conditioned medium obtained from TIVE-LTC cells (Figure S8, panels 5–8). This network was also observed in the presence of solvent treated TIVE-LTC cells (Figure S8, panels 9–12), ruling out the possibility of non-specific inhibition by solvent on tube formation. In contrast, with supernatants from TIVE-LTC cells pretreated with COX inhibitors, we observed significant inhibition of tube formation (Figure S8, panels 13–20). Higher inhibition was observed with NS-398 treated TIVE-LTC cell supernatant which had complete impairment of tube formation (Figure S8, panels 17–20).

Quantitatively, supernatant obtained from TIVE-LTC cells induced roughly 3-fold more branch points/field than TIVE cells (Figure 7C). Pretreatment of TIVE-LTC cells with either Indo or NS-398 inhibited the secretion of angiogenic factors and thereby reduced node formation by 49% and 85%, respectively (Figure 7C). Together, these results suggest that KSHV induced COX-2 not only mediates the expression of growth and angiogenic factors from latently infected endothelial cells but also their functional properties, such as angiogenesis related tube formation.

### KSHV infection induces the secretion of various MMPs and TIMPs

MMPs belong to a family of secreted or membrane-associated zinc endopeptidases capable of digesting connective tissue ECM proteins as well as basement membrane constituents [46], and have been shown to play a critical role in orchestrating cell signaling, homeostasis of the extracellular environment via proteolysing their specific substrates [47], cell-cell and cell-matrix interactions, maintaining tight junctions, and thereby contributing to the malignant phenotypes of cancers, including cell invasion, metastasis, angiogenesis and inflammatory infiltration. So far, 23 MMPs have been identified in humans, and based largely on their substrate specificity, these are divided into collagenase like MMPs (−1, −8, −13), gelatinase like MMPs (−2, −9), stromelysin or proteoglycanase like MMPs (−3, −7, −10, −11), elastase (-12), membrane type-MMPs (1–4), and unclassified MMPs. Among them, MMP-2 and MMP-9 are known to be strongly correlated with the metastatic potential of cancer cells and in particular are prognostic factors in many solid tumors [46],[48]. MMP (−1,−2,−7,−9,−13) and MT-MMP-14 expression has been shown by immunohistochemistry in AIDS-related and classic cutaneous KS lesions at various histologic stages [49] implicating them in KS tumorigenesis and invasion. Here, we assessed the role of KSHV induced COX-2 on MMPs in uninfected and KSHV infected HMVEC-d cells.

**Figure 8:**
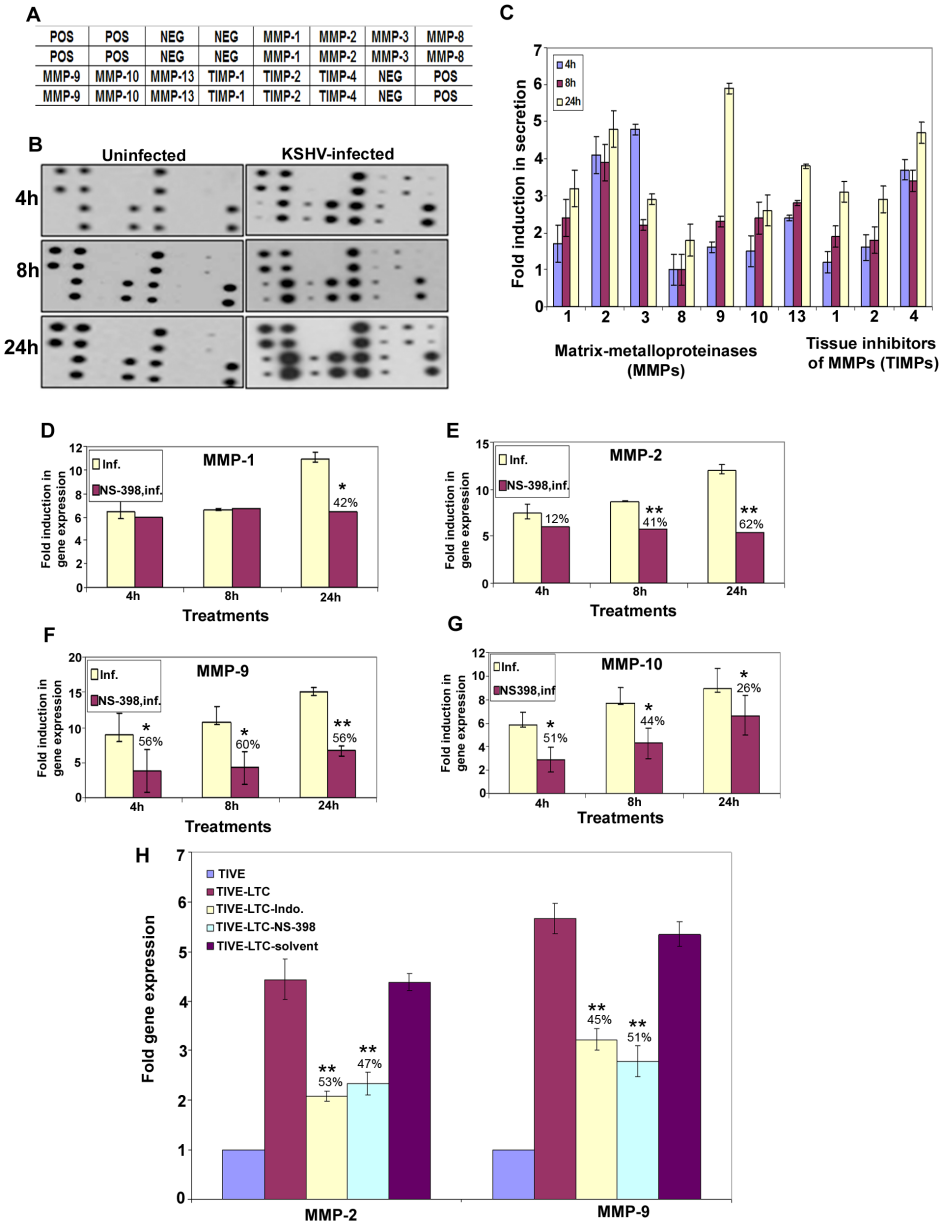
KSHV induced COX-2 regulates MMP gene expression in HMVEC-d as well as latently infected endothelial cells. (**A**) Scheme of MMPs on MMP array-1. (**B**) Representative MMP arrays showing the signals for various MMPs in the conditioned medium obtained from serum starved uninfected HMVEC-d cells or cells infected with 30 DNA copies/ cell of KSHV for 4 h, 8 h, and 24 h. (**C**) Densitometric analysis of MMP array blots measuring the release of human MMPs. The values were normalized to identical background levels using the Ray Biotech Human MMP antibody array 1 analysis tool. The fold induction in MMP secretion was calculated by dividing the respective values obtained from infected-cell supernatants with the values obtained from uninfected-cell supernatants. Each point represents the average ± SD from three independent experiments. (**D, E, F and G**) MMP gene expression. MMP-1 (**D**), MMP-2 (**E**), MMP-9 (**F**), and MMP-10 (**G**) gene expression was measured by q-RT-PCR with cDNA prepared from serum starved (8h) HMVEC-d cells infected or NS-398 treated (1h), and then infected with KSHV for 4h, 8h, and 24h. Result shows the mean ± S.D of three independent experiments. % inhibition in gene expression upon NS-398 treatment was calculated using gene expression in the presence of KSHV infected HMVEC-d cells at different time points as 100%. (**H**) MMP-2 and MMP-9 gene expression in TIVE-LTC cells upon COX inhibitor treatment. Gene expression was measured by q-RT-PCR with cDNA prepared from serum starved (24h) TIVE-LTC cells untreated or treated with either 500 µM Indo or 75 µM NS-398 for 24h. Result shows the mean ± S.D of three independent experiments. % inhibition in gene expression upon inhibitor treatment was calculated using gene expression in untreated TIVE-LTC cells as 100%. *, **, ***-statistically significant at p<0.01, p<0.005 and p<0.001 respectively.

Conditioned media collected from serum starved (8h) uninfected or KSHV infected HMVEC-d cells were used to probe for the presence of various MMPs and TIMPs using MMP antibody arrays (Figure 8A). Conditioned media from the uninfected cells had appreciable amounts of MMP-1 and -10 (Figure 8B). KSHV infection up-regulated the secretion of MMPs and TIMPs (Figures 8B and 8C) at all of the time points tested. Except for MMP-3, secretion of MMPs (-1, -2, -8, -9, -10 and -13) was enhanced in a time dependent manner with higher levels of secretion for MMP-9 and -2 (Figure 8C).

TIMPs are endogenous inhibitors of MMPs and the TIMP family consists of four distinct members, TIMP-1, -2, -3, and -4. Among these, TIMP-2 expression is constitutive and widely expressed throughout the body but TIMP-1, -3, -4 expression is inducible and often exhibits tissue specificity [46]. The balance between MMPs/TIMPs regulates ECM turnover, regulates tumor invasion and metastasis, wound healing and tissue remodeling during normal development and pathogenesis. The conditioned media from uninfected cells showed appreciable amounts of TIMPs 1 and 2 (Figures 8B and 8C). TIMP (−1, −2 and −4) secretion increased with the time post- KSHV infection (Figure 8C).

### KSHV induced COX-2 regulates the MMPs in infected endothelial cells

Effect of COX-2 inhibition was tested for a few select MMPs. MMP (−1, −2, −9 and −10) gene expression was induced during KSHV infection of HMVEC-d cells (Figures 8D-8G). NS-398 pretreatment reduced the expression of all MMPs tested (Figures 8D-8G), with the most significant inhibition of MMP-2 and MMP-9 (Figures 8E and 8F), suggesting that KSHV induced COX-2 plays a decisive role in controlling expression of KSHV infection induced proteases. Pretreatment of HMVEC-d cells with NS-398 slightly induced the expression of TIMP-1 and TIMP-2 by 1.4- and 1.3- fold, respectively as compared to 24h PI (data not shown). This data also supported the cytokine antibody array data (Table S4), where pretreatment of cells by NS-398 up-regulated the release of TIMP-2 by 2.1 fold at 24 h PI.

### COX-2 regulates MMPs in latently infected endothelial cells

To evaluate the role of COX-2 in regulating angiogenesis and invasion, we checked the expression of MMP-9 and MMP-2 (Figure 8H) in TIVE-LTC cells treated with either 500 µM Indo or 75 µM NS-398 for 24 h. Compared to TIVE cells, TIVE-LTC cells showed nearly 4.5 and 5.7-fold higher MMP-2 and MMP-9 gene expression, respectively (Figure 8H). Pretreatment of TIVE-LTC cells with either inhibitor for 24h significantly inhibited the expression of MMP-9 and MMP-2, clearly demonstrating the role of COX-2 in gene expression during KSHV latency (Figure 8H).

### KSHV induced COX-2 regulates the activity of MMPs in de-novo infected and latently infected endothelial cells

**Figure 9:**
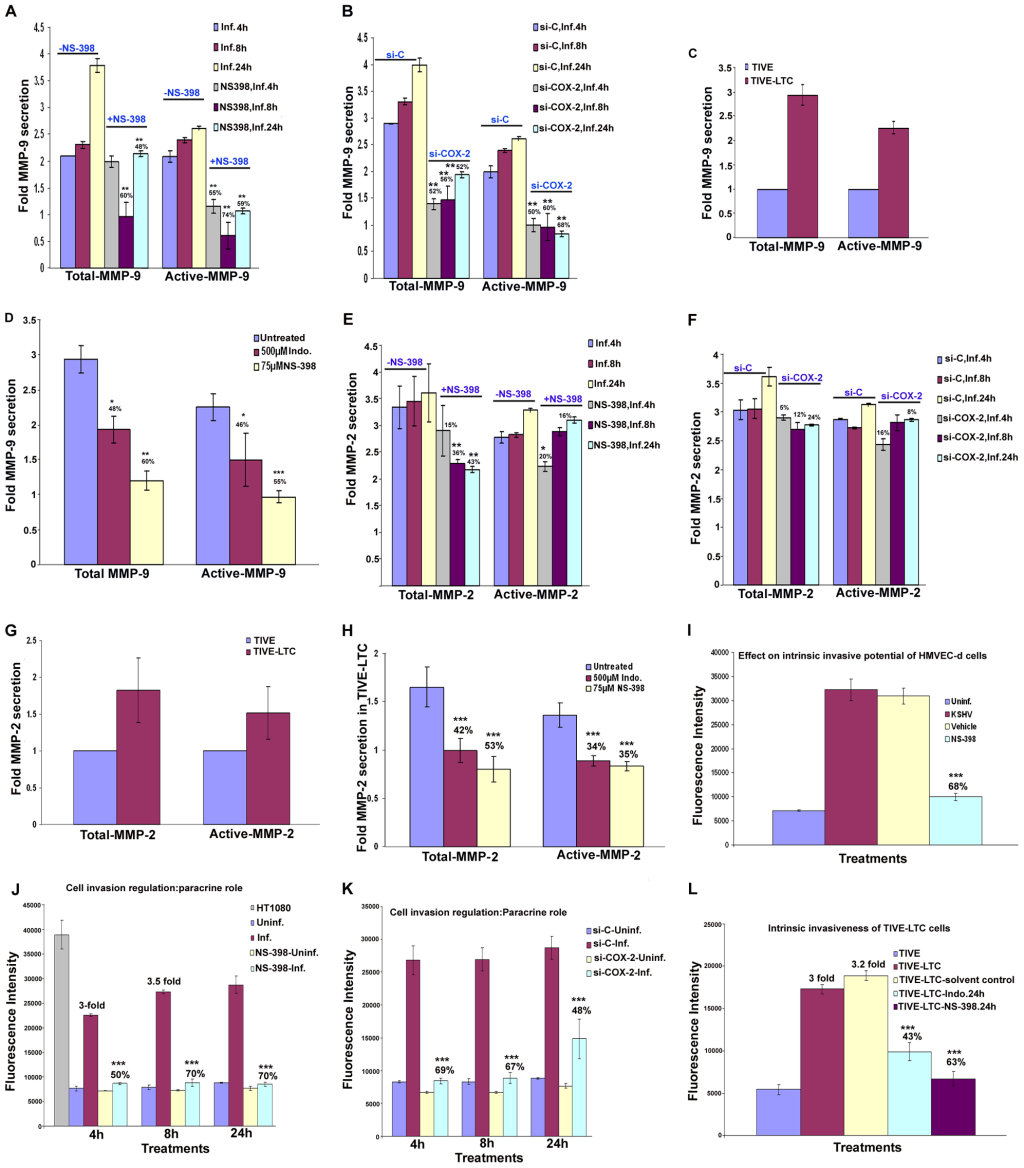
Effect of COX-2 inhibition on activity of MMPs and invasion of de novo infected HMVEC-d and latently infected TIVE-LTC cells. (**A**) Activation of MMP-9 measured by the MMP-9 assay kit in uninfected HMVEC-d cells, untreated KSHV infected, uninfected NS-398 treated or NS-398 pretreated and then infected with KSHV for 4h, 8h, and 24h. Total MMP-9 reflects the pool of pro-form and active form of MMP-9 whereas active MMP-9 exclusively represents the active protease. % inhibition of MMP-9 levels (total or active) upon NS-398 treatment was calculated considering MMP-9 levels (total or active) in the presence of KSHV infected HMVEC-d at different time points as 100%. (**B**) Activation of MMP-9 in the uninfected (4h, 8h, and 24h), KSHV infected (4h, 8h, and 24h) si-C-HMVEC-d and si-COX-2-HMVEC-d cells. % inhibition of MMP-9 levels (total or active) in si-COX-2-HMVEC-d were calculated considering MMP-9 levels (total or active) in KSHV infected si-C-HMVEC-d at different time points as 100%. (**C**) Similarly, activation of MMP-9 in conditioned medium obtained from 24h serum starved TIVE and TIVE-LTC cells was performed. The levels of total and active MMP-9 in TIVE cells were considered 1 fold for comparison. (**D**) Activation of MMP-9 in the TIVE-LTC untreated or treated with 500 µM Indo or 75 µM NS-398 for 24h. % inhibition in these protease levels (total or active) upon COX inhibition was calculated considering MMP-9 levels (total or active) in untreated TIVE-LTC cells as 100%. (**E-H**) Activation of MMP-2 was measured in the conditioned media described in **A-D** using an MMP-2 assay kit. Method of estimation and analysis was similar to **A-D**. **COX-2 regulates KSHV infected HMVEC-d cell invasion via autocrine and paracrine mechanisms.** (**I**) Effect of KSHV infection and COX-2 regulation upon the invasive potential of endothelial cells was measured by a fluorescence based invasion assay as described in Materials and Methods. Fluoresence intensity is presented and the values shown are the mean± S.D of three independent experiments. % Inhibition in invasion by NS-398 treatment was calculated considering invasion in the presence of KSHV infection as 100%. Fold induction in invasion upon KSHV infection was calculated by considering invasion of the uninfected cells as 1 fold. (**J**) **Invasion of HMVEC-d cells in the presence of supernatant from treated HMVEC-d cells.** Histogram represents the fluorescence intensity (mean± S.D of three independent experiments) of HMVEC-d cells invaded in the presence of conditioned media obtained from uninfected, KSHV infected, uninfected NS-398 treated, NS-398 pretreated and then KSHV infected for 4 h, 8 h and 24 h. HT1080 cells were used as positive control and were allowed to invade for 24 h. % Inhibition in invasion upon NS-398 treatment was calculated considering invasion in the presence of KSHV infection at the indicated time point as 100%. Fold increase in invasion upon KSHV infection was calculated by considering invasion in the presence of medium from uninfected cells at the same time as 1 fold. (**K**) Histogram represents the fluorescence intensity of HMVEC-d cells invaded in the presence of conditioned media obtained from uninfected, KSHV infected si-C-HMVEC-d and si-COX-2-HMVEC-d cells at 4 h, 8 h and 24h. % Inhibition of invasion in si-COX-2-HMVEC-d cells was calculated considering invasion in the presence of si-C-HMVEC-d cells at indicated time points as 100%. Fold increase in invasion upon KSHV infection in si-C-HMVEC-d cells was calculated by considering invasion in the presence of medium from uninfected si-COX-2-HMVEC-d cells at the same time as 1 fold. (**L**) **Effect of COX-2 inhibition on invasion of TIVE, TIVE-LTC cells in the presence of COX inhibitors.** The invasive cells were dislodged from the underside of the cell culture insert and stained with a fluorescent dye in a single step and fluorescence was determined using a fluorimeter as described before. Fluorescence associated with the invaded cells is shown and the values correspond to the mean± S.D of three independent experiments. Fold increase in the invasion of TIVE-LTC cells was calculated using the invasive potential of TIVE cells as 1 fold. *, **, ***-statistically significant at p<0.01, p<0.005 and p<0.001 respectively.

Since the antibody array data presented in 8A-8C measured the total MMP pool (sum of inactive and active) secreted, we next used an MMP-9 ELISA to differentiate between the levels of the active form of the enzyme from the total released MMP-9. Compared to uninfected cells, about 2, 2.3, and 3.8-fold induction in the release of total MMP-9 was observed at 4h, 8h, and 24h PI (Figure 9A) which was consistent with the antibody array data (Figures 8B and 8C). NS-398 treatment prior to infection inhibited total as well as active-MMP-9 secretion implicating the role of KSHV induced COX-2 in regulating MMP-9 (Figure 9A). Similar to chemical blocking, compared to si-C HMVEC-d cells, COX-2 depletion by si-COX-2 (2) significantly decreased, total as well as active, MMP-9 secretion (Figure 9B). Compared to TIVE cells, about 2.8-fold and 2.1-fold induction in the release of total MMP-9 and active-MMP-9 was observed in TIVE-LTC cells (Figure 9C). COX inhibitor treatment of TIVE-LTC inhibited total as well as active-MMP-9 secretion (Figure 9D), indicating the importance of COX-2 in regulating MMP-9.

A similar analysis was performed for total and active-MMP-2. In agreement with MMP-antibody array data, we observed a 3.3, 3.3, and 3.5-fold induction in total MMP-2 release at 4h, 8h, and 24h PI (Figures 9E and 9F). NS-398 pretreatrment decreased the secretion of total MMP-2 but not that of active MMP-2 (Figure 9E). COX-2 depletion did not effectively inhibit either total or active MMP-2 induced by KSHV (Figure 9F). Latently infected TIVE-LTC cells demonstrated roughly about 1.8 and 1.5-fold induction in the release of total and active-MMP-2 as compared to TIVE cells (Figure 9G). Similar to total MMP-2 secretion, COX inhibition also regulated the secretion of active-MMP-2 and this activity was reduced in the cells treated with NS-398 (35%) or Indo (34%) for 24h (Figure 9H).

### KSHV infection induced COX-2 augments endothelial cell invasion and functions through both autocrine and paracrine mechanisms

To assess the functionality of active MMP secretion upon KSHV infection, we performed invasion assays as described in the methods section. Figures 9I-9L represent the data obtained using *Innocyte cell invasion assay* while Figures S9 and S10 represent the invasive potential of the supernatants (used in Figures 9I-9L) as analyzed by *Chemicon cell invasion assay.* Similar results were obtained from both the methods used.

To evaluate the effect of KSHV infection on cell invasion, we infected HMVEC-d cells with KSHV at 30 DNA copies/ cell. At 24h PI, we assayed the ability of the cells to invade the ECMatrix barriers. Without chemoattractant gradients, the intrinsic invasiveness of normal HMVEC-d cells through an ECMatrix barrier was undetectable (data not shown). However, in the presence of complete growth medium as chemoattractant, some normal HMVEC-d cells succeeded in invading the ECMatrix barrier (Figure S9B; panel 1). KSHV-infected cells displayed increased invasiveness that was 4.5-fold higher than uninfected (169 cells/field versus 43 cells/field) (Figure S9B; panels 2 and 1). In contrast, NS-398 pretreated and then KSHV-infected cells showed reduced (68%) invasiveness (52 cells/field versus 169 cells/field) (Figure S9B; panels 4 and 2). This reduction in invasiveness was due to COX-2 inhibitor pretreatment rather than a non-specific effect of the NS-398 solvent as invasiveness in the solvent treated and infected cells was similar to that of cells infected with KSHV alone (164 cells/field versus 169 cells/field) (Figure S9B; panels 3 and 2).

To demonstrate whether KSHV infection could promote cell invasion in a paracrine fashion, we assessed the invasiveness of normal HMVEC-d cells in the presence of supernatants from uninfected- or KSHV-infected HMVEC-d cells. KSHV infection increased HMVEC-d cell invasiveness by 3-fold (47 versus 112 cells/field), 3.5-fold (52 versus 158 cells/field) and 3.5-fold (54 versus 180 cells/field) at 4h, 8h, and 24 h, respectively (Figures 9J and S9C; panels 1–3 versus 4–6). NS-398 pretreatment reduced KSHV promotion of cell invasion by 50% (77 versus 112 cells/field), 70% (43 versus 158 cells/field), and 70% (48 versus 180 cells/field) after 4, 8, and 24h, respectively. This indicated that KSHV induced COX-2 plays a critical role in regulation of MMPs and associated invasion (Figures 9J and S9C; panels 4–6 versus 7–9). Supernatant obtained from solvent pretreated infected cells was similar to KSHV infected cell culture supernatant (data not shown). Similar to NS-398 pretreatment, COX-2 silencing also reduced KSHV promotion of cell invasion (Figure 9K). This further validated the role of KSHV induced COX-2 in cell invasion, and suggests that KSHV infection could promote COX-2-dependent cell invasion through both autocrine and paracrine mechanisms. HT1080 cells showed maximum invasion (Figure S9C, panel 10).

### KSHV infection induced COX-2 regulates invasion of TIVE-LTC cells

Latently infected TIVE-LTC cells (169 cells/field) (Figures 9L and S10; panels 3 and 4) showed 3-fold increased invasiveness compared to TIVE cells (57 cells/field) (Figures 9L and S10; panels 1 and 2). This suggested that KSHV infection mediated secretion of proteases must be contributing to the invasive phenotype of TIVE-LTC cells. NS-398 pretreatment for 24h reduced TIVE-LTC cell invasion by 63% (62 cells/field versus 169 cells/field) (Figures 9L and S10; panels 7 and 8 versus 3 and 4) whereas Indo pretreatment reduced invasiveness by 43% (96 cells/field versus 169 cells/field) (Figures 9L and S10; panels 5 and 6 versus panels 3 and 4). Solvent treatment did not have any effect on TIVE-LTC cell invasion (176 cells/field versus 169 cells/field) (Figures 9L and S10; panels 9 and 10 versus panels 3 and 4) further validating the specific regulation of invasion by COX-2.

### KSHV infection induced PGE2 secretion regulates endothelial cell adhesion

COX-2 expression and PGE2 secretion has been shown to accelerate integrin dependent cell adhesion, migration and cell-spreading [50]. Progression of KS from early stage to an invasive and metastatic phenotype is accompanied by a series of changes associated with cytoskeleton rearrangements as well as alterations in cell-cell and cell-matrix adhesion that allows cells to invade surrounding tissues and metastasize. To understand the role of KSHV induced COX-2 in the adhesion of endothelial cells, an adhesion assay was done using untreated maxisorp plates or plates coated with polylysine or fibronectin. Adhesion in the presence of polylysine was interpreted as the result of interaction between the polyanionic cell surfaces and the polycationic layer of adsorbed polylysine and reflective of charge based interactions rather than a response to secreted factors or surface expression of various integrins. Adhesion in the presence of fibronectin was interpreted as the result of interaction with integrins as it is an extracellular matrix glycoprotein that binds to integrins. Since we observed maximum PGE2 secretion during primary infection at 2h PI [26], we collected supernatants at 2h PI to demonstrate the paracrine role of PGE2 in the presence or absence of drug and used to test their ability to induce adhesion of uninfected cells. Conditioned medium collected during later time points of infection representing latency were not used for these assays.

**Figure 10:**
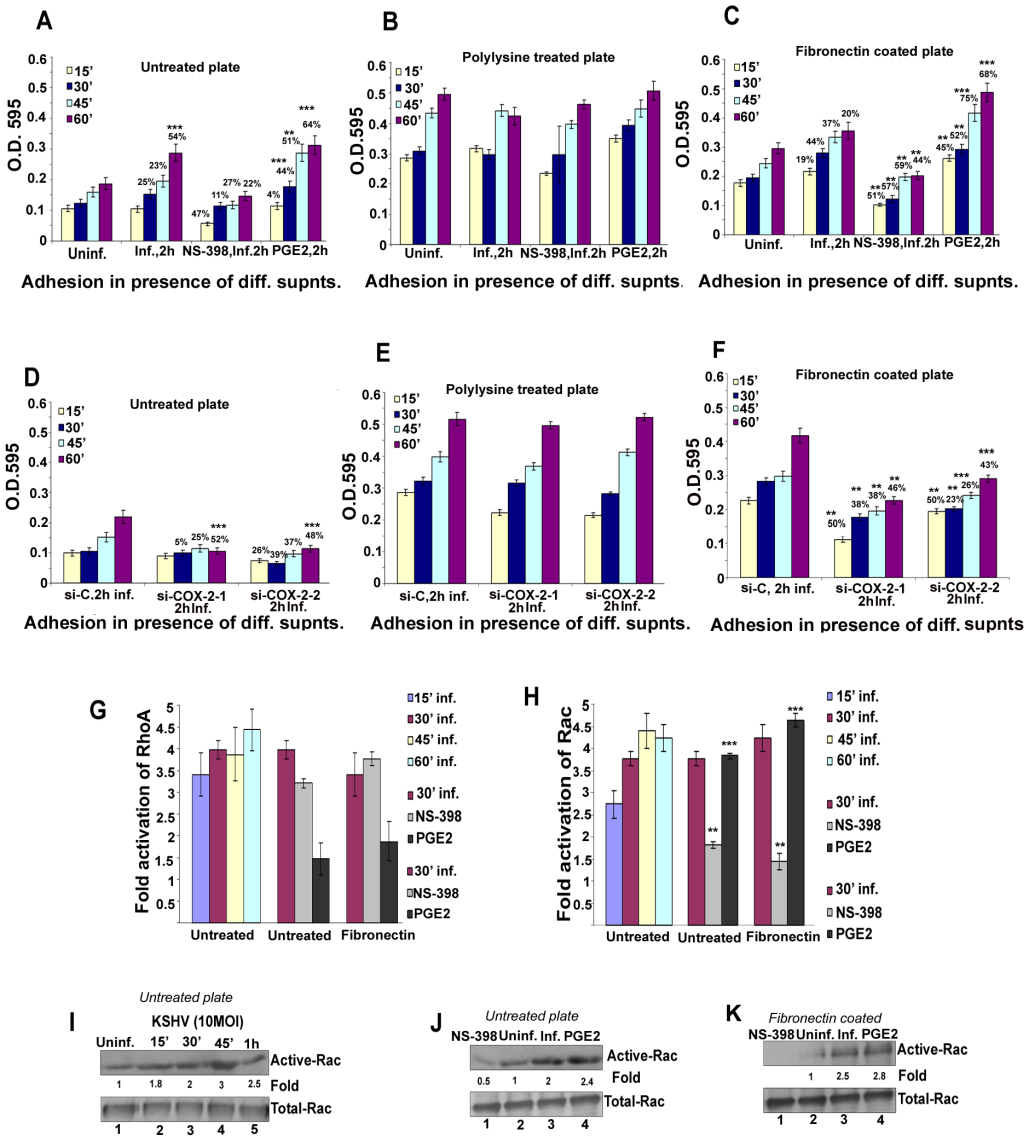
Measurement of endothelial cell adhesion. HMVEC-d cells were allowed to adhere on untreated (**A, D**), polylysine (**B, E**), or fibronectin (**C, F**) coated plates in the absence or presence of culture supernatants obtained from uninfected HMVEC-d cells (2 h), KSHV infected (2 h), NS-398 treated and then KSHV infected cells, or in the presence of serum free medium containing PGE2 (**A, B and C**) and adhesion kinetics were done at the indicated time points. A similar experiment was done using the supernatants obtained from uninfected or infected (2 h) si-C-HMVEC-d, si-COX-2 (1)-HMVEC-d, or si-COX-2 (2)-HMVEC-d cells (**D, E and F**). Adhered cells were photographed (pictures not shown). Unattached cells were removed by washing and attached cells were fixed using 4% paraformaldehyde at the indicated times and visualized by crystal violet staining. Results are provided as optical density (O.D.) and represent the mean of triplicate determinations ± S.D. (**G and H**) Histograms representing RhoA-GTPase (**G**) or Rac1-GTPase (**H**) activation in HMVEC-d cells in the presence of various supernatants (as mentioned in results) for the indicated time points on either untreated or fibronectin coated plates and measured by GLISA. Fold activation of RhoA and Rac1 is calculated by considering RhoA or Rac1-GTPase activity in the presence of uninfected cell culture supernatant as 1 fold. Each reaction was done in triplicate, and each bar represents the mean ± S.D. for three experiments. (**I, J and K**) Representative gels depicting Rac1-GTPase activation in HMVEC-d cells plated in the presence of various supernatants (as mentioned in results) for indicated time points on either untreated or fibronectin coated plates and measured using a PAK pull-down assay. Fold change of Rac1 activation is calculated by determining the band intensities and are expressed as fold increase over the cells plated in the presence of uninfected culture supernatant taken as 1- fold. Each blot is representative of a minimum of three separate experiments. **, ***-statistically significant at p<0.005 and p<0.001 respectively.

Endothelial cells were allowed to adhere in the presence of the culture supernatant obtained from uninfected endothelial cells (2h) or the cells infected with KSHV for 2h, or cells pretreated with NS-398 for 1h and then infected with KSHV for 2h. Previously, we have shown that pretreatment of HMVEC-d cells with NS-398 inhibited the secretion of PGE2, suggesting that these supernatants would be depleted of PGE2. When plated on untreated plates, compared to the adhesion of cells in the presence of supernatant from uninfected cells, adhesion of uninfected HMVEC-d cells increased in the presence of culture supernatant from KSHV infected endothelial cells (Figure 10A). Treatment of the plate surface with polylysine increased the kinetics of binding irrespective of the presence of various culture supernatants (Figure 10B). When plated on fibronectin coated plates, cell adhesion kinetics were faster in the presence of infected cell culture supernatant suggesting a role for paracrine factors in the expression of integrins and its interaction with the integrin ligand, fibronectin (Figure 10C).

To address the role of PGE2 in HMVEC-d cell adhesion, we plated the cells in the presence of serum free medium containing 1 µM PGE2 which markedly enhanced HMVEC-d adhesion to the untreated as well as fibronectin coated plates (Figures 10A and 10C). The adhesion kinetics on the fibronectin plates was faster than adhesion to the untreated plates. Compared to the polylysine coated plates, PGE2 increased cell adhesion to the untreated and fibronectin coated plates suggesting its role in regulating the expression of surface molecules, possibly integrins or adhesion molecules, on the endothelial cells to facilitate rapid adhesion.

To understand the role of COX-2 inhibition and abrogated secretion of PGE2 in endothelial cell adhesion, we used the supernatant obtained from the cells pretreated with NS-398 and then infected with KSHV. Adherence of cells in the presence of NS-398 treated KSHV infected culture supernatant was comparatively less on all plates (untreated, fibronectin coated and polylysine coated) (Figures 10A-C) but decreased appreciably in the fibronectin coated plates (Figures 10A and 10C). This further confirmed the critical role of KSHV induced COX-2/PGE2 in endothelial cell adhesion. The role of PGE2 secretion in endothelial cell adhesion was further confirmed by using culture supernatants from KSHV infected si-C-, si-COX-2-1 or si-COX-2-2 -HMVEC-d cells (Figures 10D-F). Adhesion on untreated plates or fibronectin coated plates was reduced significantly in the presence of supernatants from si-COX-2-1 and -2 and KSHV infected HMVEC-d cells when compared to si-C-infected HMVEC-d cells (Figures 10D-F). This confirmed the involvement of KSHV infection induced COX-2/PGE2 in cell adhesion. Effects were more pronounced on fibronectin coated plates suggesting that COX-2/PGE2 mediates endothelial cell integrin expression or modulation of cell adhesion molecules. These results clearly demonstrated that COX-2/PGE2 play pivotal roles in cell adhesion to the matrix, an important event in KSHV pathogenesis that has seen little exploration.

### KSHV-PGE2 regulated endothelial cell adhesion involves Rac1 activation

Rearrangement of the actin cytoskeleton is primarily controlled by members of the Rho-GTPase family such as RhoA, Rac1, and Cdc42 [51]. Our earlier studies have demonstrated the activation of these GTPases by KSHV infection and stimulation by interaction of the KSHV envelope glycoprotein gB with adherent endothelial or fibroblast cell integrins [25],[35],[52]. RhoA-GTPases are implicated in regulating morphology and adhesion because interactions between the actin cytoskeleton and adherens junctions determine cell shape and motility [51]. RhoA and Rac have been shown to be critical regulators of cell adhesion and cell spreading while COX-2/prostaglandin production has been reported to be essential for integrin-dependent Rac activation in HUVEC cells [53]. Hence, we assessed the role of KSHV infection induced PGE2 in regulating these signaling molecules.

First, we asked the question whether secreted factors participate in the activation of RhoA- or Rac-GTPases. We quantified the RhoA-GTPase activity using a RhoA-GLISA kit on the lysates prepared from cells grown on untreated plates for different time points in the presence of culture supernatants from serum starved (8 h) uninfected HMVEC-d (2 h) and KSHV infected (2 h) cells. We observed 3.4, 3.9, 3.8 and 4.5-fold RhoA-GTPase activation upon plating cells in the presence of infected cell culture supernatant for 15′, 30′, 45′ and 60′, respectively (Figure 10G). This data suggested that the factors released during KSHV infection up-regulated RhoA-GTPase in the adhering cells.

Next, to understand the role of PGE2 in the regulation of RhoA-GTPase activity of infected cells, we tested the lysates from cells plated on untreated plates for 30′ in the presence of supernatant from cells uninfected or infected for 2h, and cells pretreated with 50 µM NS-398 for 1h and then infected with KSHV for 2h. Supernatant from the cells pretreated with NS-398 moderately inhibited activation of RhoA-GTPase (Figure 10G) suggesting that PGE2 secretion might not be involved in the stimulation of RhoA in HMVEC-d cell adherence. Activation of RhoA-GTPase was also measured in the lysate from cells grown in the presence of PGE2 for 30′. Induction of RhoA was ∼50% lower when compared to RhoA-GTPase activity in the presence of infected cell culture supernatant. This suggested that PGE2 alone is not enough to induce RhoA-GTPase in adherence of endothelial cells. Similar results were obtained from lysates prepared from cells plated on fibronectin coated plates thus ruling out the possibility of PGE2 participation in the interaction of integrins modulating RhoA-GTPase activity (Figure 10G).

As Rac-GTPases also play an important role in cell spreading and cell adhesion, we analyzed the activation kinetics of Rac1 by Rac1-GLISA. We observed 2.7, 3.8, 4.4 and 4.2- fold Rac1-GTPase activation upon plating cells in the presence of infected cell culture supernatant for 15′, 30′, 45′ and 60′, respectively. This data suggested that the factors released during KSHV infection up-regulated Rac1-GTPase in adherent endothelial cells. Supernatant prepared from cells pretreated with NS-398 drastically (65%) inhibited Rac1-GTPase activation (Figure 10H) suggesting that PGE2 secretion is involved in the stimulation of Rac1 in endothelial cells. To further confirm the role of PGE2, activation of Rac1-GTPase was measured in the lysate prepared from cells grown in the presence of PGE2 for 30′. Induction of Rac1-GTPase was 4.8- fold when compared to Rac1-GTPase activity in the presence of uninfected cell culture supernatant suggesting that PGE2 is enough to induce Rac1-GTPase in the adhering endothelial cells. Similar results were obtained from lysates prepared from cells plated on fibronectin coated plates thus demonstrating the possibility that PGE2 participates in the interaction of integrins modulating Rac1-GTPase activity (Figure 10H).

Rac1 activity was further confirmed using a PAK pull-down assay and fold activation was calculated by considering the Rac1-GTPase activity in the presence of uninfected supernatant at 60′ as one fold. About 1.8, 2, 3 and 2.5- fold activation of Rac1-GTPase was observed at 15′, 30′, 45′, and 60′, respectively (Figure 10I). Supernatant from the cells pretreated with NS-398 and then infected inhibited Rac1 by 75% (Figure 10J, lanes 3 and 1). This suggested that PGE2 secretion plays an important role in Rac1 stimulation, which was further supported by the 2.4-fold activity of Rac1 in the lysates prepared from cells plated in the presence of PGE2 alone for 30′ (Figure 10J, lane 4) which was comparable to the activity observed in the presence of infected cell culture supernatant (Figure 10J, lane 4). HMVEC-d cells plated on fibronectin coated plates cultured in the presence of the supernatant obtained from the 2h infected cell showed 2.5-fold activation of Rac1 which was completely abrogated (100% inhibition) in the presence of supernatant prepared from NS-398 pretreated and then infected endothelial cells (Figure 10K, lanes 1–3). Stimulation of Rac1 activity in PGE2 stimulated HMVEC-d cells grown on fibronectin plates was comparable to the activity observed in the presence of infected cell culture supernatant (Figure 10K, lanes 3 and 4). Collectively, these results clearly demonstrated that KSHV induced COX-2/PGE2 in the infected cell microenvironment plays an important role in endothelial cell adhesion by modulating the activity of Rac1-GTPases.

### KSHV infection induced COX-2 regulates growth of TIVE-LTC cells

**Figure 11:**
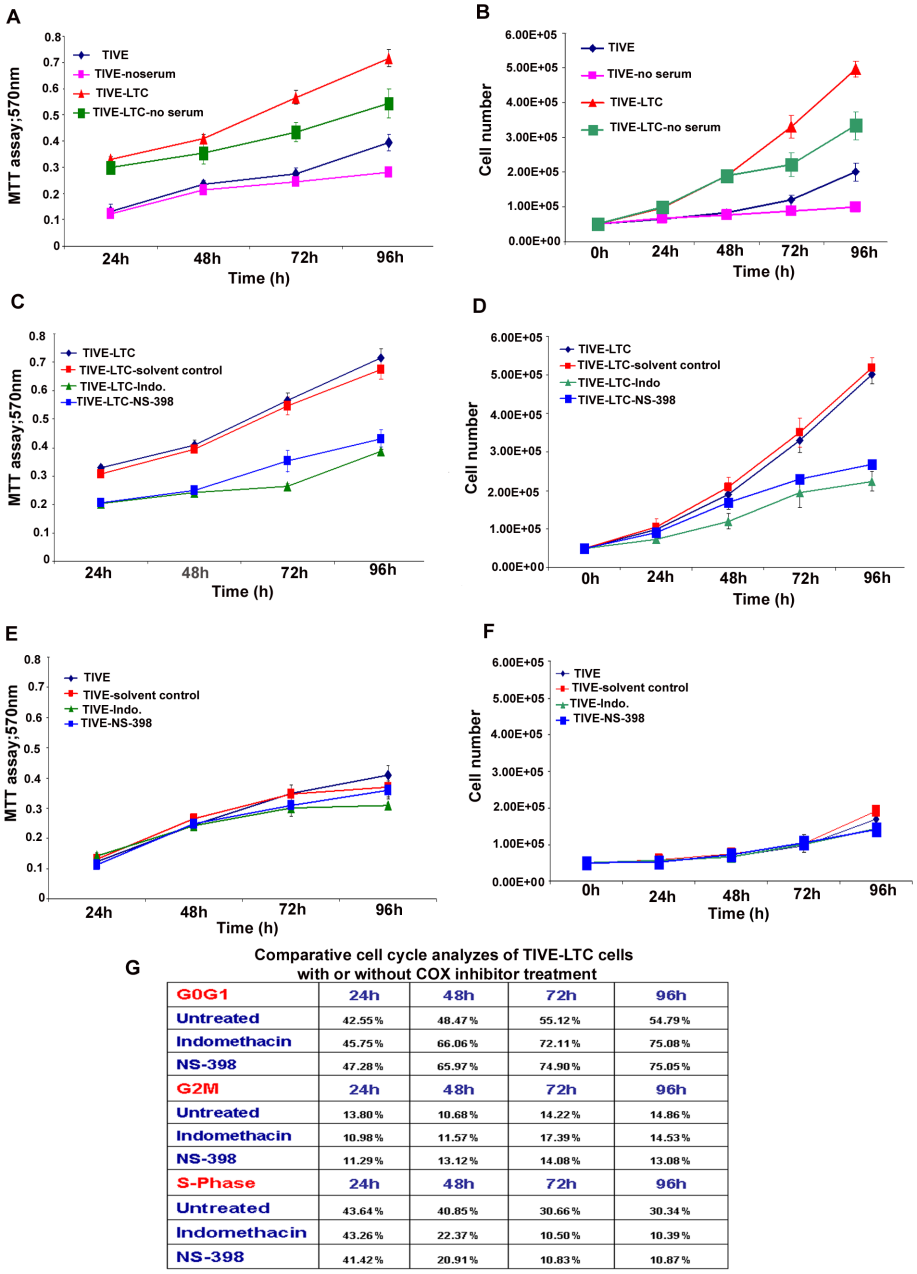
Effect of COX-2 inhibition on cell survival and cell cycle profile of latently infected TIVE-LTC cells. (**A, C and E**) **COX inhibition reduces TIVE-LTC cell proliferation.** MTT assay results shown in each panel represent the absorption at 570 nm with TIVE or TIVE-LTC cells. Cells were incubated in the presence or absence of serum or presence of 500 µM Indo, 75 µM NS-398, or solvent control for 24 h-96 h. (**B, D and F**) **COX regulates cell viability in TIVE-LTC cells.** Panels show the number of viable cells using a trypan blue exclusion assay. TIVE or TIVE-LTC cells were incubated in the presence or absence of serum in the presence of 500 µM Indo or 75 µM NS-398, or solvent control for 24 h-96 h. (**G) Comparative effects of indomethacin and NS-398 on the proliferative profile of TIVE-LTC cells.** Cell sorter analysis was performed using TIVE-LTC cells cultured in the presence and absence of indicated drugs. Propidium iodide staining of untreated, 500 µM Indo or 75 µM NS-398 treated TIVE-LTC cells was done for 24 h-96 h. The percentages of cells at specific cell-cycle phases are indicated and the numbers represent mean values of six independent experiments.

NSAIDs and derivatives of COXIBs are well documented for their anti-neoplastic activities such as inhibition of cancer cell line growth as well as initiation and promotion of apoptosis in various cancers [54]. Since KSHV induced COX-2 has an important role in regulation of viral latent gene expression that is linked to prolonged host cell survival, we assessed the effect of long term incubation of COX inhibitor (up to 96h) in latently infected endothelial cells (TIVE-LTC). To obtain the normal growth curve, cells were cultured in growth medium for 24 h, 48 h, 72 h, and 96 h before an MTT assay was performed. Results shown in Figure 11A depict growth kinetics of both cell types under normal conditions which clearly indicate that at any given time TIVE-LTC cells grow much faster than TIVE cells (Figure 11A). We next determined whether the longer duration of COX inhibitor treatment should be given in the presence of complete growth medium conditioned with serum or under serum starvation. Under serum deprivation, both cell types show reduced proliferation and the control TIVE cells appeared to be particularly dependent on serum growth factors for viability. This suggested that KSHV in TIVE-LTC cells must be inducing the secretion of growth factors that help target cell survival (Figure 11A). Even though the MTT assay measures mitochondrial activity in viable and in growth-arrested cells, its dynamic range is limited and can only be taken as an indicator for initial changes in cell survival. Therefore, we used traditional viable cell counting in similar experiments (Figure 11A). TIVE LTC cells displayed faster growth kinetics than TIVE cells thus validating the data obtained with the MTT assay (Figure 11A). Although, TIVE-LTC cells did not show profound death upon serum deprivation, TIVE cell viability was reduced by 8%, 32% and 66% at 48 h, 72 h and 96 h, respectively (Figure 11B). This further demonstrated the role of KSHV in secretion of various growth factors required for cell survival.

Next, we examined the effect of COX inhibitors on metabolism and growth index in latently infected cells. Neither fresh growth medium nor additional drug was added during the observation period for MTT and trypan blue exclusion assays. In MTT assays, we observed significant inhibition of TIVE-LTC cell metabolic activity with both drugs at all time points tested and the inhibition was marginally more with Indo for 72 h and 96 h (Figure 11C). Treatment for the same duration with solvent alone did not inhibit the metabolic activity of these cells (Figure 11C) thus validating the specific effect of COX inhibition. Similar to the MTT assay, we observed that treatment of TIVE-LTC cells with Indo reduced cell viability by 6%, 37%, 41% and 55% at 24 h, 48 h, 72 h, and 96 h (Figure 11D) while NS-398 treatment reduced cell viability by 4%, 11%, 31%, and 42% at 24 h, 48 h, 72 h, and 96 h, respectively (Figure 11D). It should be noted that TIVE and TIVE-LTC cells have hTERT which has been shown to be regulated by COX inhibitors [55]. To rule out the possibility that reduced cell viability and decreased cell metabolic activity observed in TIVE-LTC cells is not because of hTERT modulation of KSHV gene expression and related events in pathogenesis, we assessed the role of drug treatment for longer duration on the control TIVE cells (Figures 11E and 11F). NS-398 treatment did not reduce the metabolic activity of TIVE cells (Figure 11E), whereas Indo treatment affected TIVE cell metabolic activity only marginally, by about 9% and 15% at 48h and 72h of incubation, respectively (Figure 11E). Similarly, Indo treatment reduced TIVE cell viability only by 2% and 11% at 48h and 72h, respectively (Figure 11F).

Since longer incubation (48h, 72 and 96h) with the COX inhibitors reduced cell viability as observed by MTT assay, we assessed the effect of the COX inhibitor treatments on cell cycle profiling of TIVE and TIVE-LTC cells (Figure 11G, S11). To determine whether growth inhibition by COX inhibitors was attributable to cell cycle arrest, TIVE-LTC cells were treated with and without COX inhibitors for 24–96 h. According to the DNA profile, a significantly higher proportion of untreated TIVE-LTC cells were in S-phase compared to either Indo or NS-398 treated cells over longer incubation periods (48–96 h). We observed a clear anti-proliferative shift in the profile of the cell cycle parameters towards a reduced percentage of cells at the S and G2/M phases, together with an increased percentage of cells at the G1 phase. Approximately 70% reduction in S phase was observed in cells treated with COX inhibitors for 96 h (Figure 11G). There was not much change in the G2/M phase but in the drug treated cells, there was subsequent cell accumulation in the G0/G1 phase suggesting that COX inhibitors inhibit latently infected cells from crossing the G1/S boundary. Similar results were obtained with NS-398 treatment but solvent treatment did not affect the cell cycle profile of TIVE-LTC cells (data not shown) further validating the specific effect of the drug used for treatment. As 24 h treatment of TIVE-LTC cells with COX inhibitors could reduce ORF73 gene expression, we also assessed the long term effect of these drug treatments on KSHV latent gene expression. We observed 75–80% reduction in ORF73 gene expression in these cells after 96 h incubation with drugs (data not shown). Compared to TIVE–LTC, untreated TIVE cells showed shorter S phase (14%) and these cells were not affected by COX inhibitor treatment even after longer incubations (data not shown). These observations clearly suggest that COX-2 inhibitor treatment for longer durations slows proliferation of virally infected cells accompanied by reduced viral latent gene expression and thereby subsequently reducing the secretion of growth factors required for infected cell survival.

## Discussion

**Figure 12:**
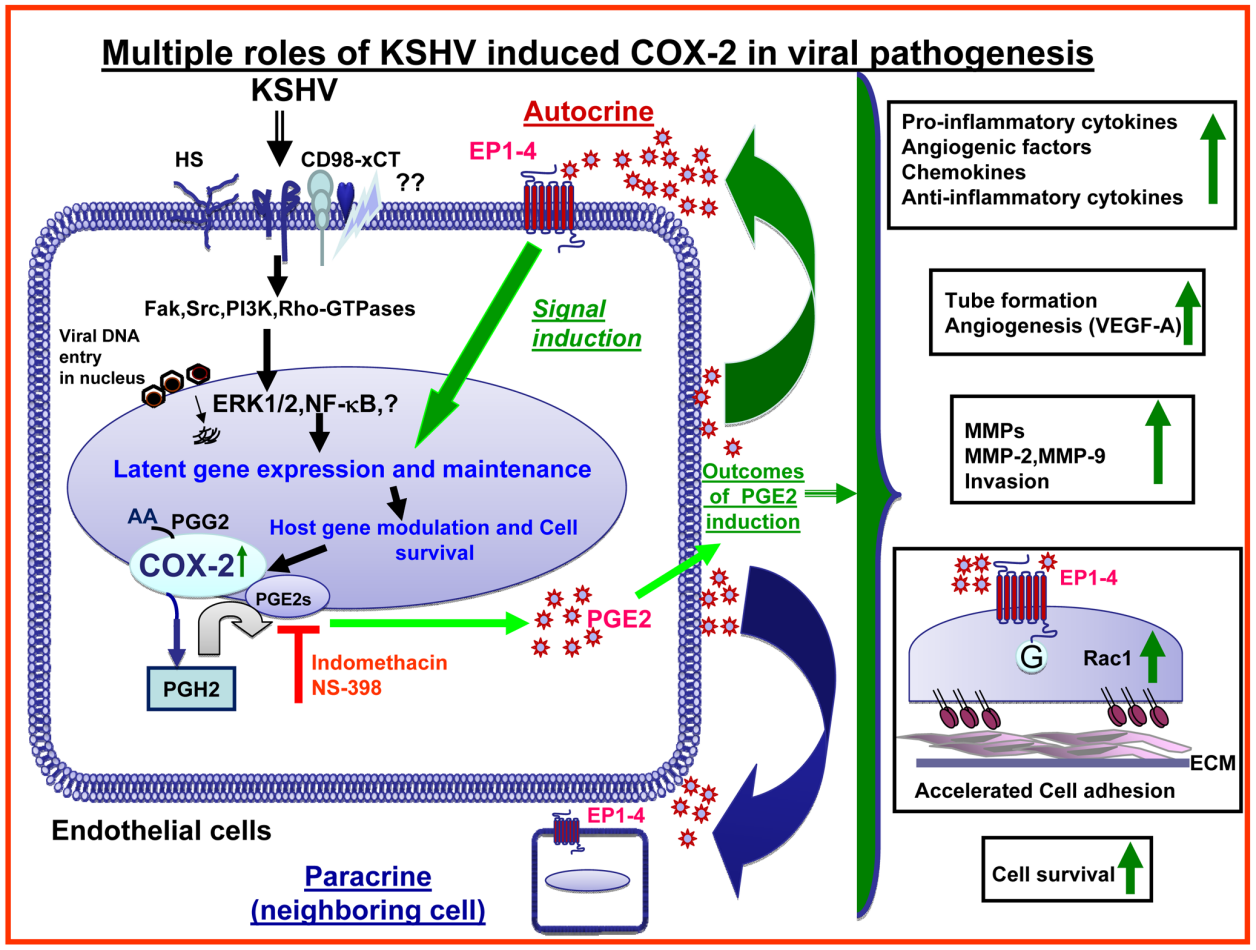
Schematic diagram depicting the multiple outcomes of KSHV induced COX-2 in endothelial cells, consequences and role in pathogenesis. In vitro KSHV infection of HMVEC-d cells involves binding of the virus to the cell surface heparan sulfate (HS) molecules via its envelope glycoproteins gpK8.1A and gB [26],[34],[35],[52], followed by interaction with integrins and xCT molecules. Virus interaction with target cell triggers pre-existing signal cascades facilitating virus entry, delivery of viral genome into the target cell nucleus and reprograms host gene expression required for various growth, angiogenic and invasive factors and one which being COX-2 [26]. Data presented here show appreciable COX-2 gene expression at 5d PI of endothelial cells, in latently infected TIVE-LTC cells and in KS lesions. COX-2 catalyzes the synthesis of PGH2 from arachidonic acid (AA) through an unstable intermediate PGG2 [26]. PGH2 is converted by mPGES to PGE2, which is released to the infected cell supernatant where it can mediate its downstream effects through either an autocrine or paracrine mechanism on a neighboring infected or uninfected cell via their interactions through the family of seven transmembrane G-protein-coupled rhodopsin-type EP (1–4) receptors. KSHV induced COX-2 regulates multiple events involved in KS pathogenesis such as secretion of pro-inflammatory cytokines, growth and angiogenic factors, anti-inflammatory cytokines, and MMPs and TIMPs. In addition, COX-2 induction also regulated infection related cell adhesion to the ECM, invasion through the matrix and angiogenesis related capillary tube formation. We demonstrated that KSHV infection induced COX-2/PGE2 also stimulates the induction of Rac1-GTPases in adhering endothelial cells. Interestingly, our study also demonstrates that KSHV infection induced COX-2 potentially modulates the survival and proliferation of latently infected endothelial cells. In summary, together with the down-regulation of viral latent gene expression upon COX-2 inhibition, our study suggests that KSHV hijacks host cellular machinery and manipulates cellular inducible angiogenic stress response gene COX-2 to its advantage to aggravate pathogenesis, cell survival and its persistence in the target cell.

KS, a chronic inflammation associated tumor, is the most common aggressive malignancy among untreated HIV-infected patients. Progression of KS lesions, with its spindle shaped cells of endothelial origin, neovascular structures and inflammatory cells, is believed to be profoundly driven by the autocrine and paracrine loops of ICs, GFs and angiogenic factors present in the lesion microenvironment [2]. Hence, to effectively control and eliminate KS lesions, it is very important to understand the driving forces behind the initiation and maintenance of these secreted factors. KSHV latent gene expression observed in KS endothelial cells and the lytic gene expression observed in a limited percentage of KS inflammatory cells probably contributes to the up-regulation of cellular host factors and a synergy between host and viral factors could be contributing to the sustained induction of inflammatory molecules and progression of lesions. Our study unravels the multifactorial complexity of KSHV-host interactions governing KS progression/pathogenesis in conjunction with host factor COX-2 and its inflammatory metabolite PGE2 (Figure 12). The exhaustive studies presented here demonstrate that COX-2/PGE2 induction upon KSHV infection is an excellent example of synergy between host and viral factors, where COX-2 operates like a central player and performs dual functions of controlling downstream consequences of KSHV infection as well as latent viral gene expression. COX-2 not only helps maintain an angiogenic and inflammatory cytokine rich KSHV permissive microenvironment but also sustains viral latent gene expression critical for infected cell survival. Our findings also suggest the potential for COX-2 inhibition based therapies in treating angio-proliferative KS lesions as COX-2 silencing, as well as treatment with COX-2 chemical inhibitors, could effectively reduce inflammation, angiogenesis, cell adhesion, cell invasion and cell signaling events during KSHV de novo and latent infection.

Our study illustrates a continuous loop of events where COX-2 behaves like an intermediate in controlling KSHV pathogenesis and latency. However, the mechanism behind sustained COX-2 protein levels and tight regulation of its activity is still missing in this loop. Attractive answer to this question would be the interplay of viral proteins or, PGE2 per se or sustained levels of secreted factors or signal molecules like NF-κB [56]. Studies are underway to understand the transcriptional and post-transcriptional regulation of COX-2 during KSHV infection.

### Role of KSHV induced COX-2/PGE2 in viral pathogenesis

Expression of COX-2 in KS lesions, the detection of higher levels of PGE2 in KS tissue compared to surrounding normal tissue [57], significant over-expression of COX-2 in the early inflammatory/angiogenic stage as well as in the late nodular stage of classic and epidemic forms of KS lesions [58], together with our studies demonstrating COX-2 in KS lesions (skin tissue and lymph node), endothelial cells infected for 5 days and latently infected endothelial cells (TIVE-LTC) (Figures 1 and 2) emphasizes that prostaglandin cascade components are actively involved in KS pathogenesis and strengthens the role of COX/PGE2 in KSHV biology. Fold inductions for COX-2 and m-PGES-1 in HMVEC-d (5d inf.) cells with about 50–60% infection and TIVE-LTC cells expressing LANA in 50–60% of the cells were comparable. Our data demonstrate that 50–60% of TIVE-LTC cells were ORF73 positive and these cells were also positive for COX-2. In addition, COX-2 staining was also seen in the cells in close proximity to the uninfected cells which could be due to paracrine COX-2 stimulation by growth factors induced upon KSHV infection.

This study for the first time systematically evaluated the downstream consequences of KSHV induced COX-2/PGE2 by using COX-2 inhibition strategies involving parallel chemical inhibition and gene silencing approachs. This study also illustrates that cytokine secretion upon KSHV infection is not a random event but follows tightly regulated kinetics (Figure 4 and Table S3). It also indicates that inhibition of cytokines via inhibitor treatment is highly selective (Figure 5 and Table S4). For example, levels of IFN-γ, MCP-2, MCP-3, TARC, GCP-2, MIP-3α, Eotaxins, CK-β8-1, PDGF-BB, MCSF, G-CSF, GMCSF, angiogenin, VEGF, SDF-1, SCF, TGF-β1, leptin, and ILs (-3, -4 and -15) could be inhibited either by treatment with COX-2 inhibitor or NF-κB inhibitor [56]. Cytokines, like ILs (−5, −6 and −10) and GRO-α, were strongly inhibited by NF-κB inhibition, but not by COX-2 inhibition. In contrast, IL-1α, IL-1β, IL12-p40, TNF-α, IP-10, NAP-2, Oncostatin M, thrombopoeitin, FGFs (−4, −6, −7 and −9), Flt3-ligand, Fractalkine, IGFBPs and Osteoprotegerin were strongly inhibited by COX-2 inhibitor pretreatment demonstrating the specificity of downstream pathways regulated via COX-2 and NF-kB. Interestingly, KSHV infection induces sustained levels of NF-κB [56]. This, together with the fact that PGE2 itself can activate NF-κB, suggests the potential involvement of the COX-2-NFκB-COX-2/PGE2 axis during KSHV infection. Treatment of infected endothelial cells with Indo or NS-398 reduced the nuclear translocation of p65 (an indication of NF-κB activity) by 46% and 58%, respectively (data not shown), which suggests the involvement of COX-2/NF-κB in regulation of secreted factors. COX-2 inhibition could not impair cytokine secretion by 100%, which suggests the importance of viral and other host factors in controlling these cytokines.

To establish a lifelong successful infection in an immunocompetent host, KSHV must be utilizing an impressive array of immune modulatory mechanisms, one of which appears to be the induction of COX-2/PGE2. For example, the ability of COX-2/PGE2 to mediate regulation of IFN and RANTES (Table S4), involved in the recruitment of inflammatory cells, represents one strategy which KSHV utilizes to evade the host immune system. KSHV induced COX-2/PGE2 also regulates VEGF-A and VEGF-C (Figure 6), the multifunctional potent immunosuppressive cytokines that profoundly regulate cell growth, adhesion, angiogenesis, proliferation and differentiation, as well as FGF-4, PDGF, TGF-β, IL-1β and IL-6 which are known to up-regulate VEGF expression. Interestingly, increased COX-2 mRNA expression and PGE2 secretion has been shown to enhance VEGF mRNA expression suggesting a direct role for PGE2 in stimulation of angiogenesis [59]. VEGF-C and –A are also known to induce lymphangiogenesis and play key roles in lymphatic reprogramming involving the conversion of blood endothelial cells (BEC) to lymphatic endothelial cells (LEC) [60]; an important event in KS pathogenesis. In addition, reduced levels of IL-3 (Table S4), a known inducer of lymphatic markers Prox-1 and podoplanin in HMVEC-d cells, by COX-2 inhibition delineates a very significant role of COX-2 in KSHV lymphangiogenesis [61]. VEGF-A was found to be tightly regulated by COX-2/PGE2 in de novo infected HMVEC-d and TIVE-LTC cells (Figure 6). Similar to the involvement of COX-2/PGE2 in cytokine secretion in many inflammation related diseases [62], KSHV induced COX-2 also plays important roles in the expression and secretion of various chemokines, growth and angiogenic factors and thereby controls the angiogenesis and tube formation (Figure 7, S7, and S8) events of KS pathogenesis.

COX-2 has been implicated in invasiveness, angiogenesis and distant metastases of many cancers [63]. MMP secretion has been associated with many viruses, including EBV [64],[65],[66], hepatitis B virus (HBV) [67] and HIV-1 [68]. However, little is known about the functional role of MMPs and TIMPs in KSHV infection and KS. Our studies report for the first time the role of KSHV induced MMP-2 and MMP-9 (Figure 8) secretion in HMVEC-d and latently infected TIVE-LTC cells. HMVEC-d cell MMP secretion kinetics was different from the published kinetics in HUVEC cells [12], which could be due to cell type specific patterns of MMP secretion. TIVE-LTC cells secreted diminished levels of MMP-2 when compared to de novo infected cells (Figure 9E and 9G), therefore COX-2 inhibition could not effectively down-regulate active MMP-2 secretion during de novo infection (Figure 9E and 9F) compared to TIVE-LTC cells. Higher active-MMP-2 levels even upon COX-2 inhibitor treatment suggest that either MMP-2 might be controlled by factors other than COX-2/PGE2 or the inhibitor dose was insufficient to regulate its secretion. COX-2 inhibition specifically abrogated the expression and secretion of MMP-9 (9A-9D) in de novo infected as well as latently infected endothelial cells, a protease responsible for metastatic potential and triggering the angiogenic switch [16]. COX-2/PGE2 probably regulates MMP-9 at the transcriptional level by activating transcription factors like AP-1, Ets2, NF-κB, and Sp1 [33]. MMP-9 has the potential to increase VEGF release, its bioavailability to bind to VEGF receptors on endothelial cells, and thus leading to an angiogenic loop that eventually will result in cell migration, cell proliferation and angiogenesis [69]. COX-2 might be a key player regulating many feedback loops in cytokine-MMP interactions, including chemokines such as RANTES, TNF-α, GM-CSF and SDF-1 which induce MMP-9 that can cleave a spectrum of pro-cytokines like TNF-α, pro-TGFβ and IL-1β. COX-2 levels could modulate multifunctional TIMP-1 and -2, which can inhibit MMP activities and can activate the FAK/PI3-K or Src/PI3-K pathways [70],[71]. Along with the other above mentioned functions, PGE2 released early (2 h) during infection also enhanced the kinetics of endothelial cell adhesion (Figure 10). Increased adhesion could be attributed to various crucial factors controlled by PGE2, such as the activation of Rac1-GTPases (Figure 10), release of SDF-1 [72], IL-1β, TNF-α, along with the cell surface expression of adhesion molecules and integrins like αVβ3 and β1 [53]. Collectively, our study underscores the importance of the COX-2-PGE2-MMP-9 axis in KS pathogenesis and suggests that COX-2 inhibitors have tremendous therapeutic potential in KSHV biology.

### Role of COX-2/PGE2 in establishment of viral latency

Many viruses, such as herpes simplex virus (HSV), human cytomegalovirus (HCMV), pseudorabies virus (PRV), human herpesvirus-6 (HHV-6), EBV, murine herpesvirus 68 (MHV-68), and human T-cell leukemia virus type 1 (HTLV-1), have been shown to induce COX-2 and release PGE2 that participate in viral lytic replication. In contrast, the role of COX-2/PGE2 in KSHV infection appears to be different as our previous [26] and current studies demonstrate that COX-2/PGE2 not only regulates inflammation associated events via modulating cytokine secretion but also controls viral latency (Figure 2E) which has been shown to be essential for viral genome maintenance and host cell survival [23],[73].

COX-2 has been shown to play direct roles in the enchancement of tumorigenic and angiogenic factors in KSHV independent cancers. However, COX-2 appears to play an additional unique role in the context of KS pathogenesis and in creating a KS lesion microenvironment rich in cytokines since it also participates in KSHV biology by virtue of its ability to aid in the establishment and maintenance of latent gene expression. Since KSHV latent genes themselves are shown to be powerful mediators of anti-apoptosis, cell survival, as well as gene regulation including the induction of COX-2 and other cytokines and angiogenic factors [37],[56], our study exposes an interesting regulatory loop between KSHV induction of COX-2 expression, COX-2's role in the establishment and maintenance of KSHV latency and the induction of cytokines and angiogenic factors that sustains the KSHV-permissive microenvironment. Hence the observed effect of COX-2 inhibition on cytokines, angiogenesis and cell survival in the context of KSHV infected cells is probably not just due to COX-2's role as the direct activator of these processes but probably due to the combinatorial effect on reduction in KSHV latent gene expression and its downstream consequences including reduction in COX-2 expression, and cell survival. The observed regulatory loop of COX-2 in KSHV biology opens up a new avenue that could be potentially exploited for an effective control of KSHV and KS lesions.

Besides overcoming host intrinsic, innate and adaptive immune responses, survival of latently infected cells requires the constant blockage of apoptosis. Intriguingly, we observed that COX-2/PGE2 is involved in regulating latently infected TIVE-LTC cell survival (Figure 11). COX-2 inhibition for longer duration could shorten S phase, arrest TIVE-LTC cells at G1/S phase accompanied by further lowered ORF73 gene expression (Figure 11G and S11). An important question to be answered is whether exogenous supplementation of PGE2 in cells treated with COX-2 inhibitors will rescue cells from undergoing death or cell cycle arrest. Nevertheless, our study reveals that KSHV infection induced COX-2/PGE2 is an important anchor linking viral gene expression, GFs and cell survival in latently infected cells. We have demonstrated a reduction in ORF73 gene expression at early as well as later time points of COX-2 inhibitor treatment and a reduction in the cells in S phase at later times of drug incubation (Figure 11G). One of the key properties of LANA is to stimulate cells in the S phase entry [73] by relocalizing GSK3-β and stabilizing β-catenin, thereby manipulating the GSK3β-β-catenin complex. Decreased ORF73 gene expression upon COX inhibitor treatment and the shortened S phase of latently infected cells raises the possibility that COX inhibition might be disturbing the ability of LANA to interact with GSK-3β and the Rb protein required for G_1_/S progression. In other words KSHV might be utilizing COX-2 and PGE2 to stabilize these complexes required for successful latency. ORF73 gene expression is also critical to overcome the host chromatin-binding protein BRD4-and BRD2/RING3-stimulated G_1_/S arrest [74], therefore reduced ORF73 gene expression upon COX-2 inhibition could be pushing the cells to G1/S arrest.

This study also raises important questions including the role of COX-2/PGE2 in viral episome maintenance and their effects on LANA protein levels. As PGE2 is known to stimulate several signaling events (JNK-1, ERK1/2, PKC, PI3K-AKT, HPK1, Src), second messengers including cAMP, calcium and reactive oxygen species (ROS), and modulate various transcription factors (Ets-1, Sp1, Oct-1, STAT-3, AP-1, ELK-1, hypoxia inducible factor-1α, and β-catenin) [75],[76],[77],[78],[79],[80],[81],[82],[83],[84],[85],[86],[87],[88],[89], COX-2/PGE2 could be mediating their effect on KSHV latency via one or more of these factors. It is interesting to note that some of the PGE2 activated transcription factors (Sp1, HIF-1α and AP-1) are well established for their role in modulation of viral latency (ORF73) and lytic (ORF50) promoters [90],[91],[92]. Studies to decipher the molecular pathway of PGE2 mediated ORF73 promoter regulation and viral latency is under investigation. The ability of KSHV to utilize pro-inflammatory molecules to maintain latent gene expression demonstrates the plasticity of the KSHV genome and its adaptability to host surveillance.

Currently, there are no methods available to eliminate the latent infection of herpesviruses. Slow proliferation of KSHV latently infected cells accompanied by reduced viral latent gene expression upon treatment with COX-2 inhibitors (Figure 11) strongly demonstrates that COX-2 is an excellent target for controlling KSHV latency.

At present, two classes of COX inhibitors are currently available for use in humans. NSAIDs inhibit both COX-1 and COX-2 while COXIBs are COX-2 selective with very little effect on COX-1 and consequently have been described as “healthier, dedicated, and more targeted” [93]. Despite a few COX-independent actions [43] of chemical inhibitors, these are still the most promising drugs for treating inflammation associated cancers and are recognized as potentially effective antiviral, anti-mitogenic and anti-angiogenic compounds. Observations such as reduced b-FGF and VEGF secretion and MMP-9 regulation reveals that COX-2 inhibitors possess the potential to be exploited in the *in vivo* model to better understand their benefits as an adjuvant to the currently available chemotherapy for KS. Interestingly, PGE2 has also been shown to modulate several functions associated with rapamycin, a drug shown to be efficacious against PEL cell lines [94]. COX-2 inhibitors show additive effects when used as part of a combination therapy since they potentiate the effect of IFN-α in HCV infection [95] and IFN-γ in several tumors [96],[97]. Hence, their inclusion in combination with KS chemotherapy, radiation, and biological therapies might prove to be beneficial in the KS scenario. Effective inhibition of COX-2 could lead to reduced KSHV infection of endothelial cells which may in turn reduce the accompanying inflammation and KS lesion progression.

## Supporting Information

Figure S1 Magnified views of COX-2 and ORF73 expression in sections shown in Figures 1A, 1C. Arrow head (black) indicates COX-2 staining. Arrow (black) in panels 2 and 4 indicates ORF73 staining. Magnifications: 20X.
(10.57 MB TIF)

Figure S2 COX-2 staining in various tissue sections on tissue microarray. Brown color indicates COX-2 staining. Panels 1-54 represent different tissue sections on KS-TMA as mentioned. 1 (skin), 2 (small bowel), 3 (skin), 4 (mouth), 5(mouth), 6 (lymph node), 7 (lymph node), 8 (lung), 9 (lymph node), 10 (lung), 11 (small bowel), 12 (lymph node), 13 (lymph node), 14 (skin), 15 (eye orbit), 16 (epiglottis), 17 (lymph node), 18 (tonsil), 19 (skin), 20 (lymph node), 21(anus), 22 (skin), 23(anus), 24 (lymph node), 25 (skin), 26 (mouth), 27 (skin), 28 (skin), 29 (skin), 30 (lymph node), 31 (nasopharynx), 32 (hypopharynx), 33 (soft tissue mass), 34 (skin), 35 (skin), 36 (skin), 37 (skin), 38 (skin), 39 (lymph node), 40 (skin), 41 (skin), 42 (lymph node), 43 (lymph node), 44 (skin), 45 (tonsil), 46 (lymph node), 47 (skin), 48 (skin), 49 (skin), 50 (spleen), 51 (skin), 52 (lymph node), 53 (lymph node), and 54 (tongue). Magnifications: 10X.
(8.13 MB TIF)

Figure S3 Spontaneous lytic reactivation of KSHV. HMVEC-d cells grown to 80-90% confluence were infected with 30 DNA copies/ cell of KSHV for 2h, 4days and 5 days, fixed and permeabilised. Uninfected and infected cells were incubated with ORF73 (green; Panels 1-6) and ORF59 (red; Panels 7-18) specific antibodies, washed, incubated with secondary antibodies, washed, counterstained with DAPI and examined under a fluorescence microscope.
(4.08 MB TIF)

Figure S4 Effect of inhibitors on PGE2 secretion. Cell free culture supernatants of TIVE-LTC untreated or treated with indicated doses of COX inhibitors for 1h, 8h, and 24h were used to measure the levels of PGE2 by ELISA. Each reaction was done in duplicate, and each point represents the average ± SD from three independent experiments. **, *** -statistically significant at p<0.005 and p<0.001 respectively.
(2.14 MB TIF)

Figure S5 (A) Representative Cytokine arrays showing the signals for various cytokines in the conditioned medium from serum starved HMVEC-d cells infected 30 DNA copies/ cell of KSHV for 96h and the conditioned medium from serum starved HMVEC-d cells infected with KSHV (30 DNA copies/ cell) for 96h that was pre-incubated with 100 µg/ml of heparin for 1h. (B) Representative Cytokine arrays showing the signals for cytokines in the conditioned medium from serum starved uninfected HMVEC-d cells or cells infected for 4h with 30 DNA copies/ cell of KSHV.
(0.88 MB TIF)

Figure S6 (A-D) Effect of NS-398 or COX-2 silencing on KSHV infection induced VEGF-C, GM-CSF, GRO, and RANTES gene expression. Each histogram depicts the fold induction in gene expression of KSHV infected, or NS-398 pretreated for 1h and then infected with KSHV, or si-COX-2-2-HMVEC-d/si-C-HMVEC-d cells infected with 30 DNA copies/ cell of KSHV for 4h, 8h, and 24h. The % inhibition was calculated by considering cytokine gene expression in the infected cells at the respective time of measurement as 100%. Each reaction was done in quadruplicate, and each bar represents the average ± SD of four independent experiments. *, **, ***-statistically significant at p<0.01, p<0.005 and p<0.001 respectively.
(2.69 MB TIF)

Figure S7 (A) Effect of COX-2 inhibition by Indo or NS-398 on KSHV-induced uninfected HMVEC-d capillary tube formation. Photomicrograph showing HMVEC-d cell tube formation with various supernatants in a matrigel coated 96-well plate. (a) endothelial basal medium; Supernatants were from: (b and c) cells treated with inhibitors alone; (d) cells infected with KSHV for 24h; (e) cells pretreated with Indo (500 µM,1h) and then infected with KSHV for 24h; (f) cells pretreated with the NS-398 (50 µM,1h) and then infected with KSHV for 24h; (g) cells pretreated with the Suramin; (h) cells cultured in the presence of EGM-2 complete growth medium (with serum); (i) cells pretreated with solvent (vehicle) alone and then infected with KSHV for 24h. After 16h incubation, plates were examined for capillary tube formation under an inverted microscope and photographed at 10X magnification. Circles represent branch points/field (connections among cells), and the line represents length of the capillary tubes. Each assay was done in triplicate and each experiment was repeated three times and the qualitative differences were further analyzed by using morphometric analysis in metamorph software to obtain quantitative information regarding tube length. (B) Effect of COX-2 inhibition by si-RNA on KSHV-induced uninfected HMVEC-d capillary tube formation. Photomicrograph showing HMVEC-d cell tube formation with various supernatants in a matrigel coated 96-well plate. Supernatants were from: (a) cells silenced for lamin (control) and then left uninfected for 24h; (b) cells silenced for COX-2 using COX-2-1 construct and then left uninfected for 24h; (c): cells silenced for COX-2 using COX-2-2 construct and then left uninfected for 24h; (d) cells silenced for lamin construct and then infected with KSHV for 24h; (e) cells silenced for COX-2 using COX-2-1 construct and then infected with KSHV for 24h; (f) cells silenced for COX-2 using COX-2-2 construct and then infected with KSHV for 24h. (g, h and i) Additional set of observations for treatments similar to d, e and f, respectively.
(8.38 MB TIF)

Figure S8 Effect of COX-2 inhibition on the ability of supernatants obtained from latently infected TIVE-LTC cells to form tubular network in uninfected HMVEC-d cells. Photomicrographs showing HMVEC-d cell tube formation with various supernatants in a matrigel coated 24-well plate. Supernatants were from: (1-4) 24h serum starved TIVE cells; (5-8) 24h serum starved TIVE-LTC cells; (9-12) TIVE-LTC cells treated with solvent control (24h); (13-16) TIVE-LTC cells treated with 500 µM Indo (24h); (17-20) TIVE-LTC cells treated with 75 µM NS-398 (24h). Experiment was performed and analyzed as described in Figure S6. Panels 4, 8, 12, 16 and 20 are the same view as 3, 7, 11, 15, and 19, respectively (viewed under UV light). In these experiments, Calcein-AM loaded HMVEC-d cells were used for tube formation.
(2.99 MB TIF)

Figure S9 COX-2 regulates KSHV infected HMVEC-d cell invasion via autocrine and paracrine mechanisms. (A) Schematic for invasion assays used; 1) innocyte cell invasion assay and 2) Chemicon cell invasion assay. (B-C) Effect of KSHV infection and COX-2 regulation upon the invasive potential of endothelial cells was measured by Chemicon cell invasion assay as described in Materials and Methods. Representative pictures of uninfected, KSHV infected, solvent control treated and KSHV infected, NS-398 pretreated and then infected HMVEC-d cells migrated through the ECMatrix layer are shown. All invasion assays were carried out over 24h as described in Materials and Methods. Each assay was done in duplicate and each experiment was repeated three times and analyzed by considering five fields. Representative pictures of HMVEC-d cells invaded in the presence of conditioned media obtained from uninfected, KSHV infected, uninfected NS-398 treated, NS-398 pretreated and then KSHV infected for 4h, 8h and 24h.
(6.78 MB TIF)

Figure S10 Effect of COX-2 inhibition on invasion of latently infected TIVE-LTC cells. Representative pictures of TIVE, TIVE-LTC untreated, solvent control treated, 500 µM Indo, or 75 µM NS-398 treated TIVE-LTC cells migrated through the ECMatrix layer are shown. All the invasion assays were carried out for 24h as described in Materials and Methods. 1 and 2, 3 and 4, 5 and 6, 7 and 8, 9 and 10 are pictures from duplicate wells. All assays were done by two methods using three independent experiments and analyzing five fields.
(7.48 MB TIF)

Figure S11 Effect of COX inhibitors on the cell cycle profile of latently infected endothelial cells. Representative pictures for cell cycle analysis after propidium iodide staining of untreated, 500 µM Indo or 75 µM NS-398 treated TIVE-LTC cells for 24h-96h. The percentages of cells at specific cell-cycle phases are indicated and the numbers represent mean values of six independent experiments.
(4.10 MB TIF)

Table S1 COX-2 staining in Kaposi Sarcoma tumor sections (ACSR KS Screening TMA 09-1).
(0.19 MB TIF)

Table S2 Sequences of real time-primers used for cytokine gene expression.
(1.98 MB TIF)

Table S3 Cytokine Profiling was done using Ray Biotech human cytokine antibody 3.1 according to manufacturer's instructions. Arrays were incubated with supernatants o/n at 4°C, and then developed. The densitometry values were substituted in analysis tool and entire array values were normalized to the same background level. Fold induction in cytokine levels was calculated considering levels in uninfected supernatant as 1 -fold. Data is the average of 3 arrays per treatment.
(1.97 MB TIF)

Table S4 Fold reductions upon NS-398 pretreatment before KSHV infection or COX-2 silencing upon KSHV infection are presented and fold reduction was calculated with respect to the levels upon infection at the respective time point of infection. Values in red show the fold induction in levels with respect to their corresponding levels upon infection. Data is divided into groups 1, 2, and 3, which are represented in blue, black and red colors, respectively. Group 1 includes the cytokines inhibited by both kind of COX-2 inhibition (chemical as well as silencing), group 2 includes the cytokines inhibited by chemical inhibitor treatment alone, not reduced by COX-2 knockdown, and group 3 includes the cytokines up-regulated upon COX-2 inhibition.
(1.08 MB TIF)
