# Supplementary figures and images for: Kaposi's Sarcoma Associated Herpes Virus (KSHV) Induced COX-2: A Key Factor in Latency, Inflammation, Angiogenesis, Cell Survival and Invasion
Source: PLoS Pathog. 2010 Feb 12;6(2):e1000777. doi: 10.1371/journal.ppat.1000777 (PMC2820536; doi:10.1371/journal.ppat.1000777)

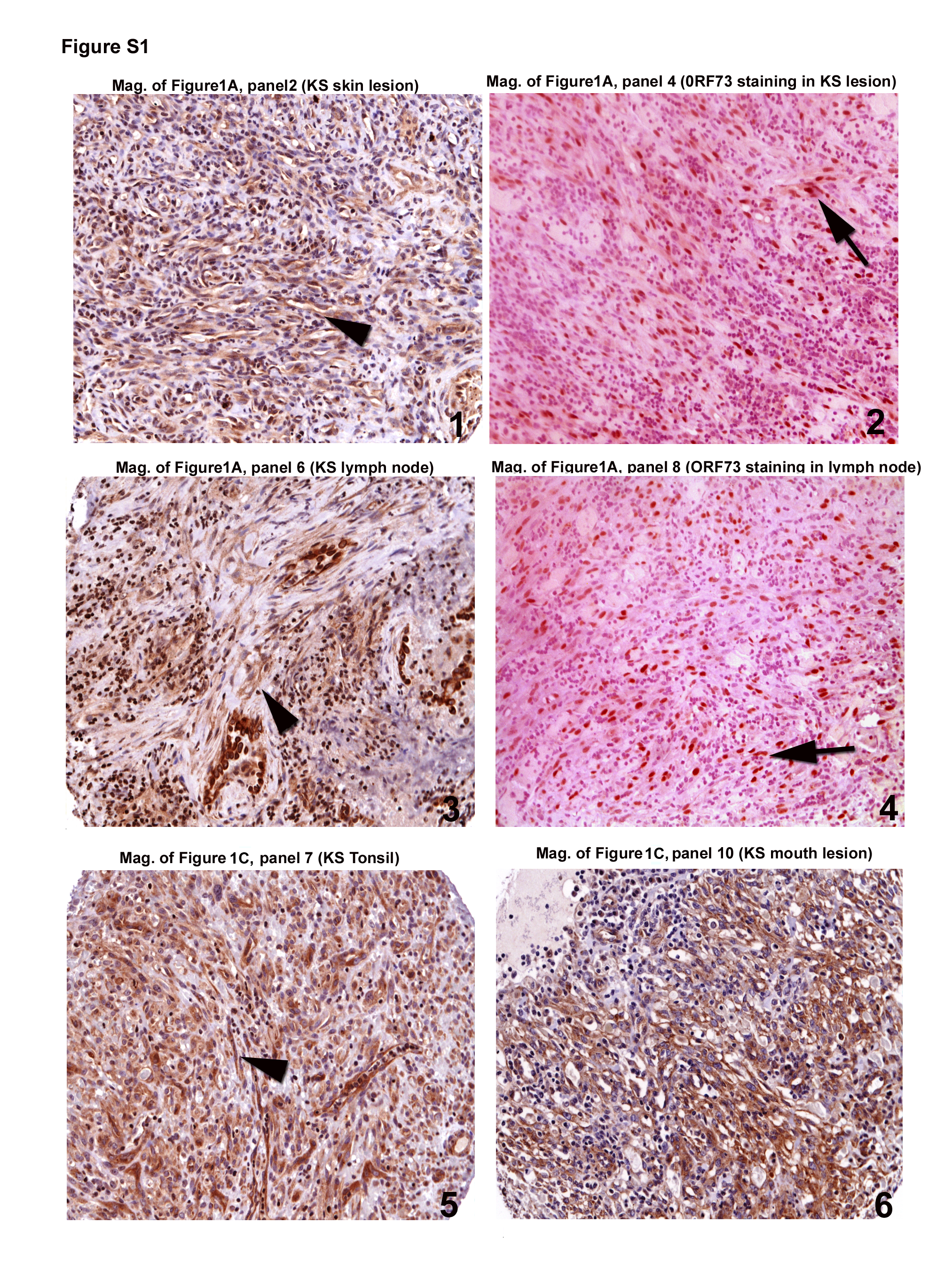

Supplement: Figure S1 — Magnified views of COX-2 and ORF73 expression in sections shown in Figures 1A, 1C. Arrow head (black) indicates COX-2 staining. Arrow (black) in panels 2 and 4 indicates ORF73 staining. Magnifications: 20X. (10.57 MB TIF) [file ppat.1000777.s001.tif]

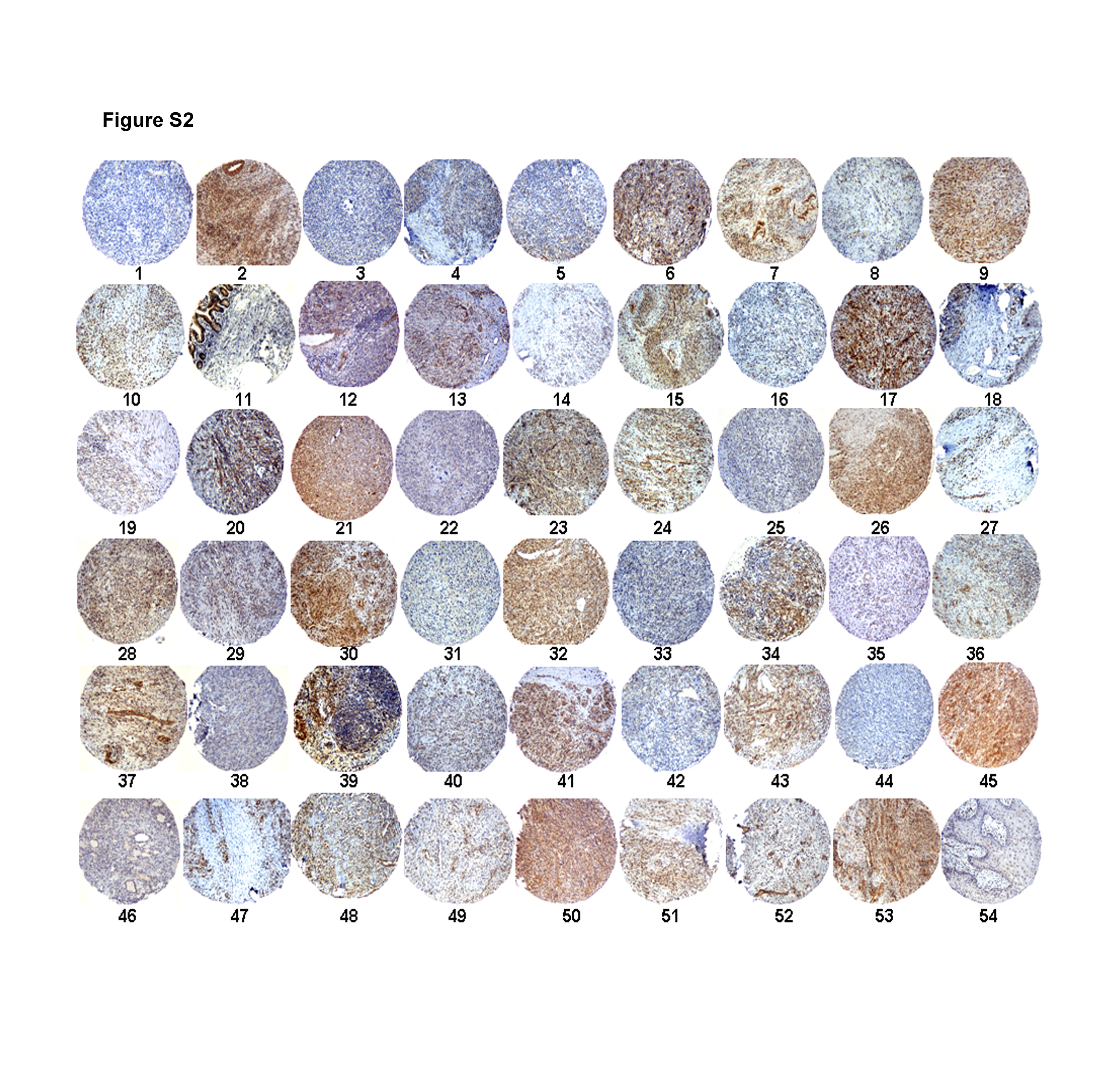

Supplement: Figure S2 — COX-2 staining in various tissue sections on tissue microarray. Brown color indicates COX-2 staining. Panels 1-54 represent different tissue sections on KS-TMA as mentioned. 1 (skin), 2 (small bowel), 3 (skin), 4 (mouth), 5(mouth), 6 (lymph node), 7 (lymph node), 8 (lung), 9 (lymph node), 10 (lung), 11 (small bowel), 12 (lymph node), 13 (lymph node), 14 (skin), 15 (eye orbit), 16 (epiglottis), 17 (lymph node), 18 (tonsil), 19 (skin), 20 (lymph node), 21(anus), 22 (skin), 23(anus), 24 (lymph node), 25 (skin), 26 (mouth), 27 (skin), 28 (skin), 29 (skin), 30 (lymph node), 31 (nasopharynx), 32 (hypopharynx), 33 (soft tissue mass), 34 (skin), 35 (skin), 36 (skin), 37 (skin), 38 (skin), 39 (lymph node), 40 (skin), 41 (skin), 42 (lymph node), 43 (lymph node), 44 (skin), 45 (tonsil), 46 (lymph node), 47 (skin), 48 (skin), 49 (skin), 50 (spleen), 51 (skin), 52 (lymph node), 53 (lymph node), and 54 (tongue). Magnifications: 10X. (8.13 MB TIF) [file ppat.1000777.s002.tif]

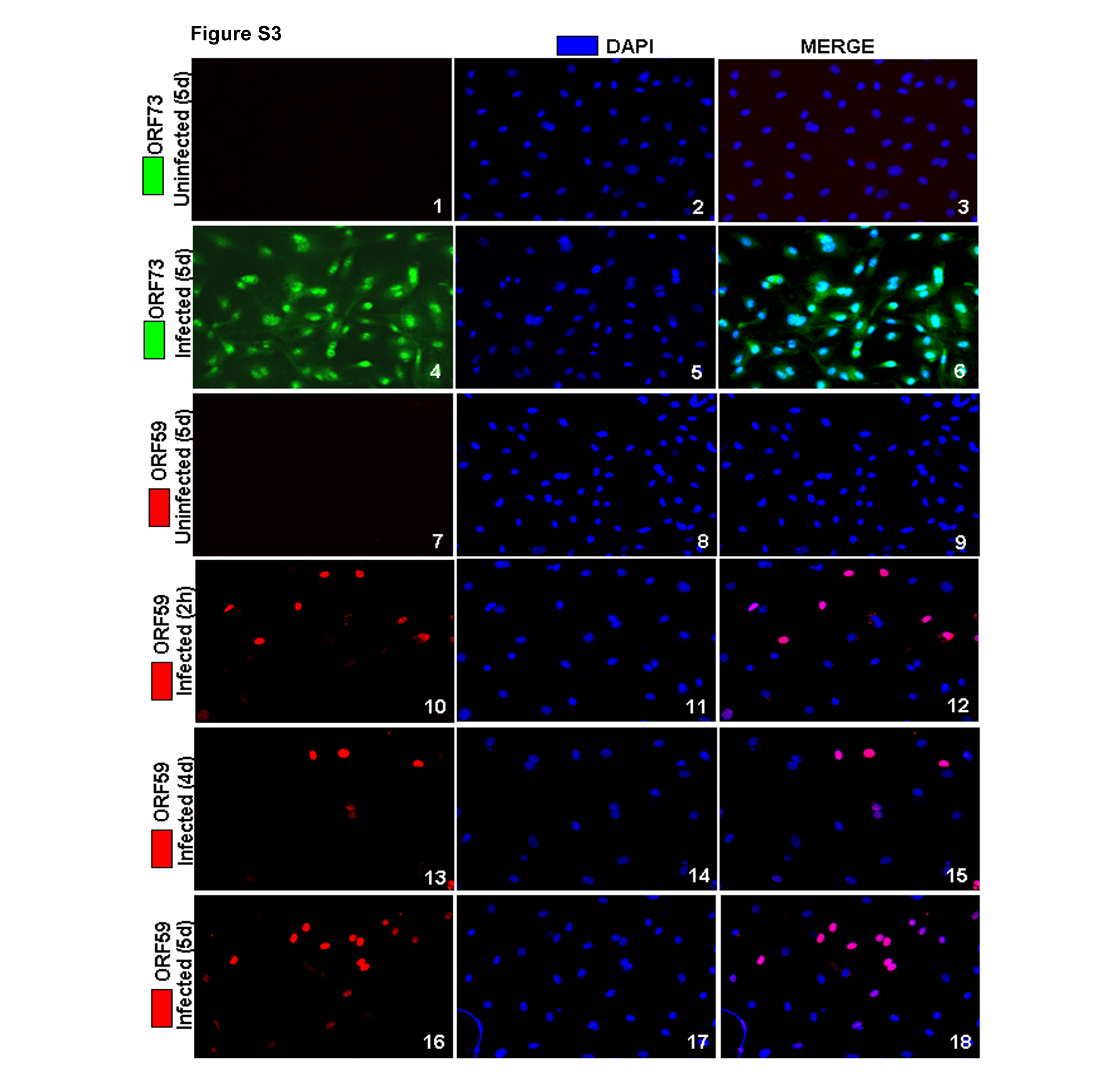

Supplement: Figure S3 — Spontaneous lytic reactivation of KSHV. HMVEC-d cells grown to 80-90% confluence were infected with 30 DNA copies/ cell of KSHV for 2h, 4days and 5 days, fixed and permeabilised. Uninfected and infected cells were incubated with ORF73 (green; Panels 1-6) and ORF59 (red; Panels 7-18) specific antibodies, washed, incubated with secondary antibodies, washed, counterstained with DAPI and examined under a fluorescence microscope. (4.08 MB TIF) [file ppat.1000777.s003.tif]

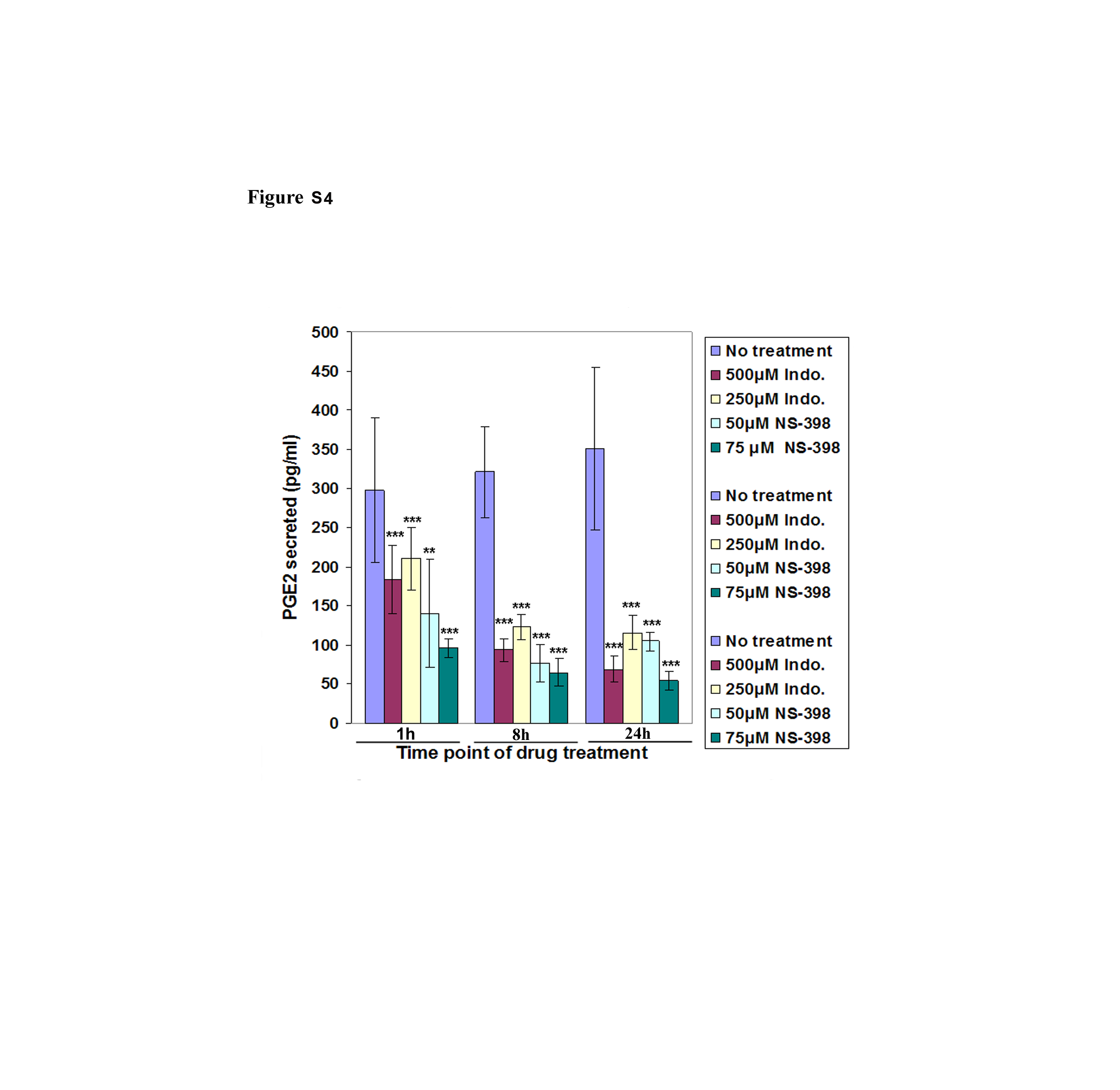

Supplement: Figure S4 — Effect of inhibitors on PGE2 secretion. Cell free culture supernatants of TIVE-LTC untreated or treated with indicated doses of COX inhibitors for 1h, 8h, and 24h were used to measure the levels of PGE2 by ELISA. Each reaction was done in duplicate, and each point represents the average ± SD from three independent experiments. **, *** -statistically significant at p<0.005 and p<0.001 respectively. (2.14 MB TIF) [file ppat.1000777.s004.tif]

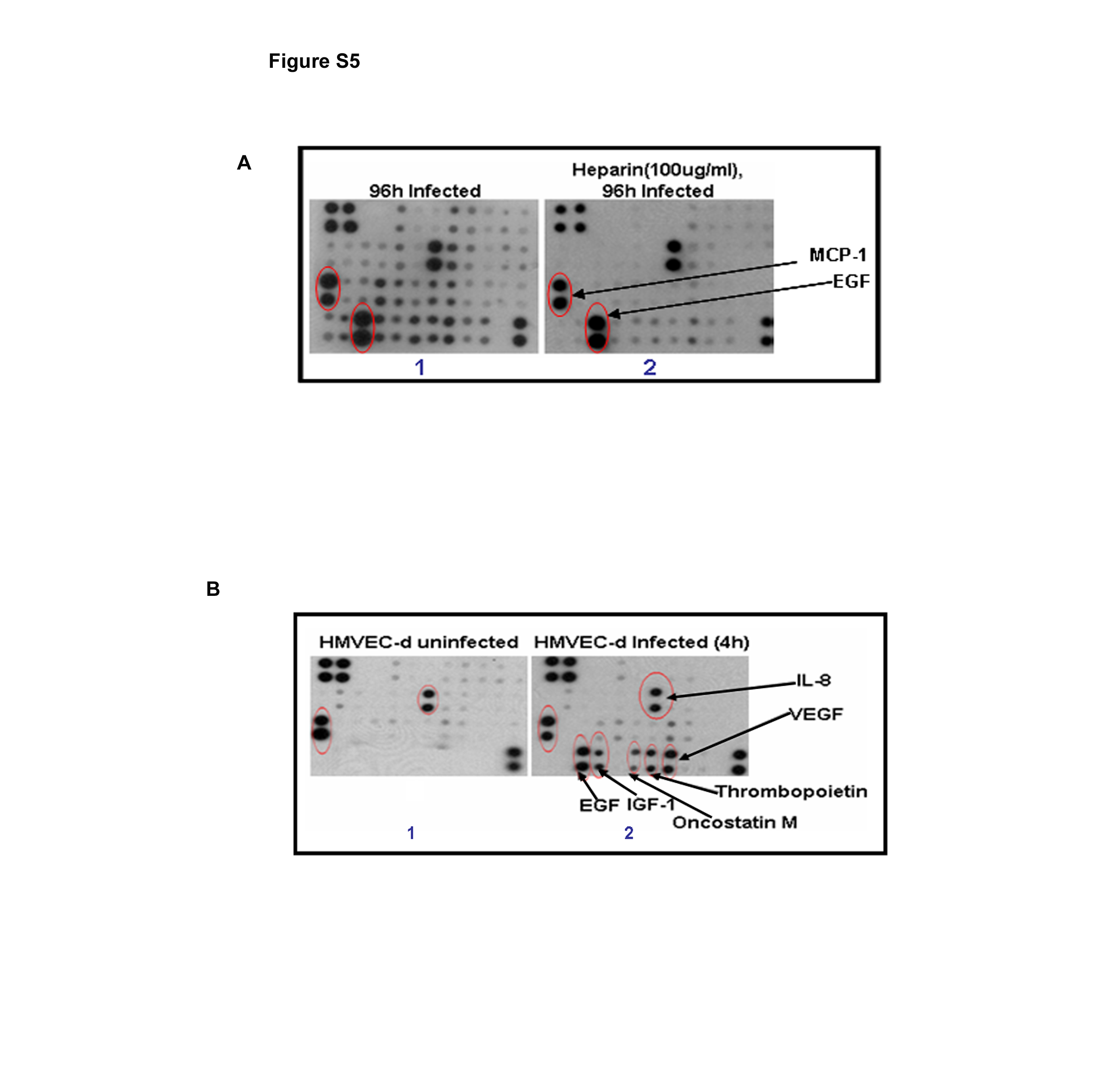

Supplement: Figure S5 — (A) Representative Cytokine arrays showing the signals for various cytokines in the conditioned medium from serum starved HMVEC-d cells infected 30 DNA copies/ cell of KSHV for 96h and the conditioned medium from serum starved HMVEC-d cells infected with KSHV (30 DNA copies/ cell) for 96h that was pre-incubated with 100 µg/ml of heparin for 1h. (B) Representative Cytokine arrays showing the signals for cytokines in the conditioned medium from serum starved uninfected HMVEC-d cells or cells infected for 4h with 30 DNA copies/ cell of KSHV. (0.88 MB TIF) [file ppat.1000777.s005.tif]

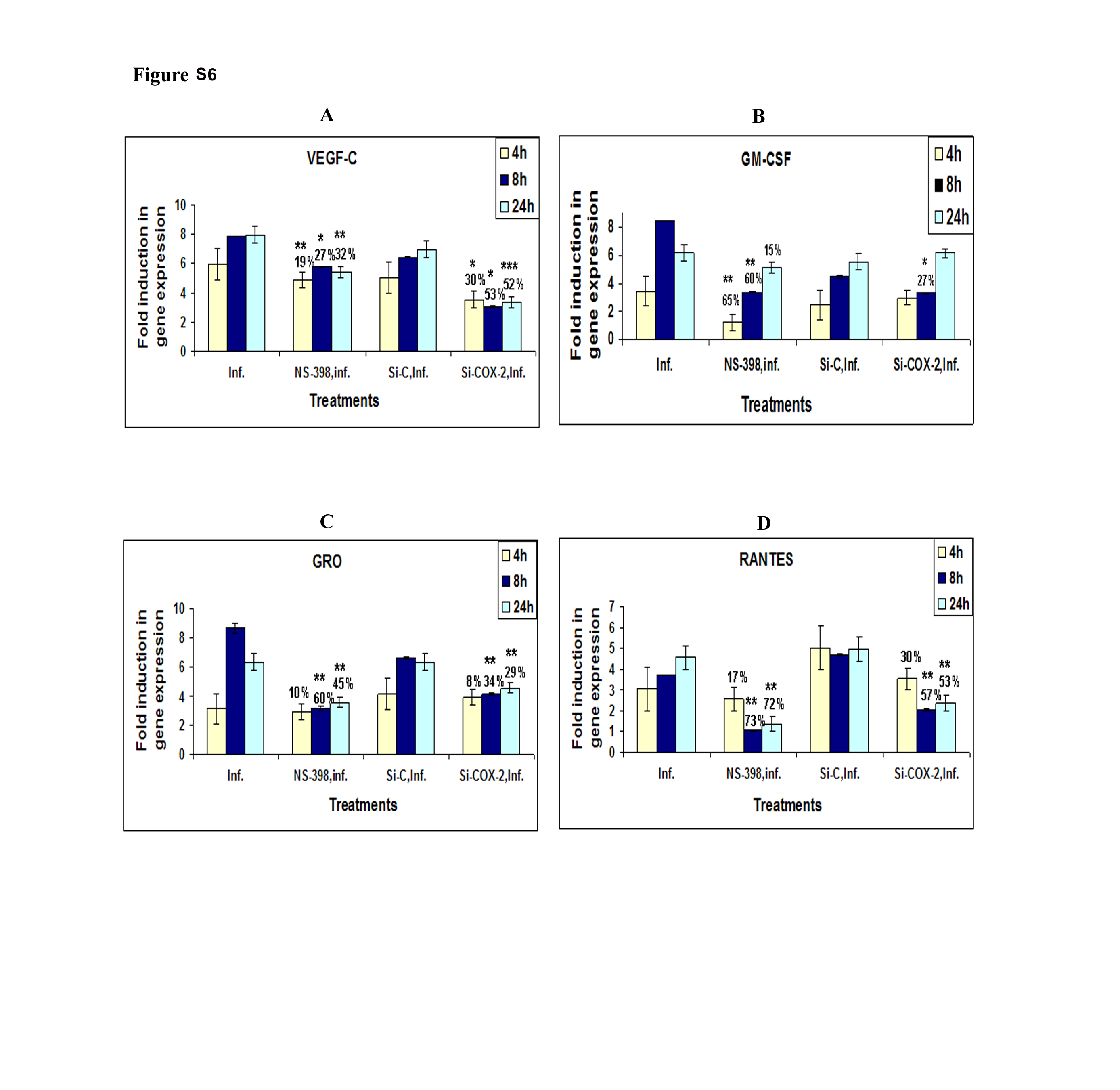

Supplement: Figure S6 — (A-D) Effect of NS-398 or COX-2 silencing on KSHV infection induced VEGF-C, GM-CSF, GRO, and RANTES gene expression. Each histogram depicts the fold induction in gene expression of KSHV infected, or NS-398 pretreated for 1h and then infected with KSHV, or si-COX-2-2-HMVEC-d/si-C-HMVEC-d cells infected with 30 DNA copies/ cell of KSHV for 4h, 8h, and 24h. The % inhibition was calculated by considering cytokine gene expression in the infected cells at the respective time of measurement as 100%. Each reaction was done in quadruplicate, and each bar represents the average ± SD of four independent experiments. *, **, ***-statistically significant at p<0.01, p<0.005 and p<0.001 respectively. (2.69 MB TIF) [file ppat.1000777.s006.tif]

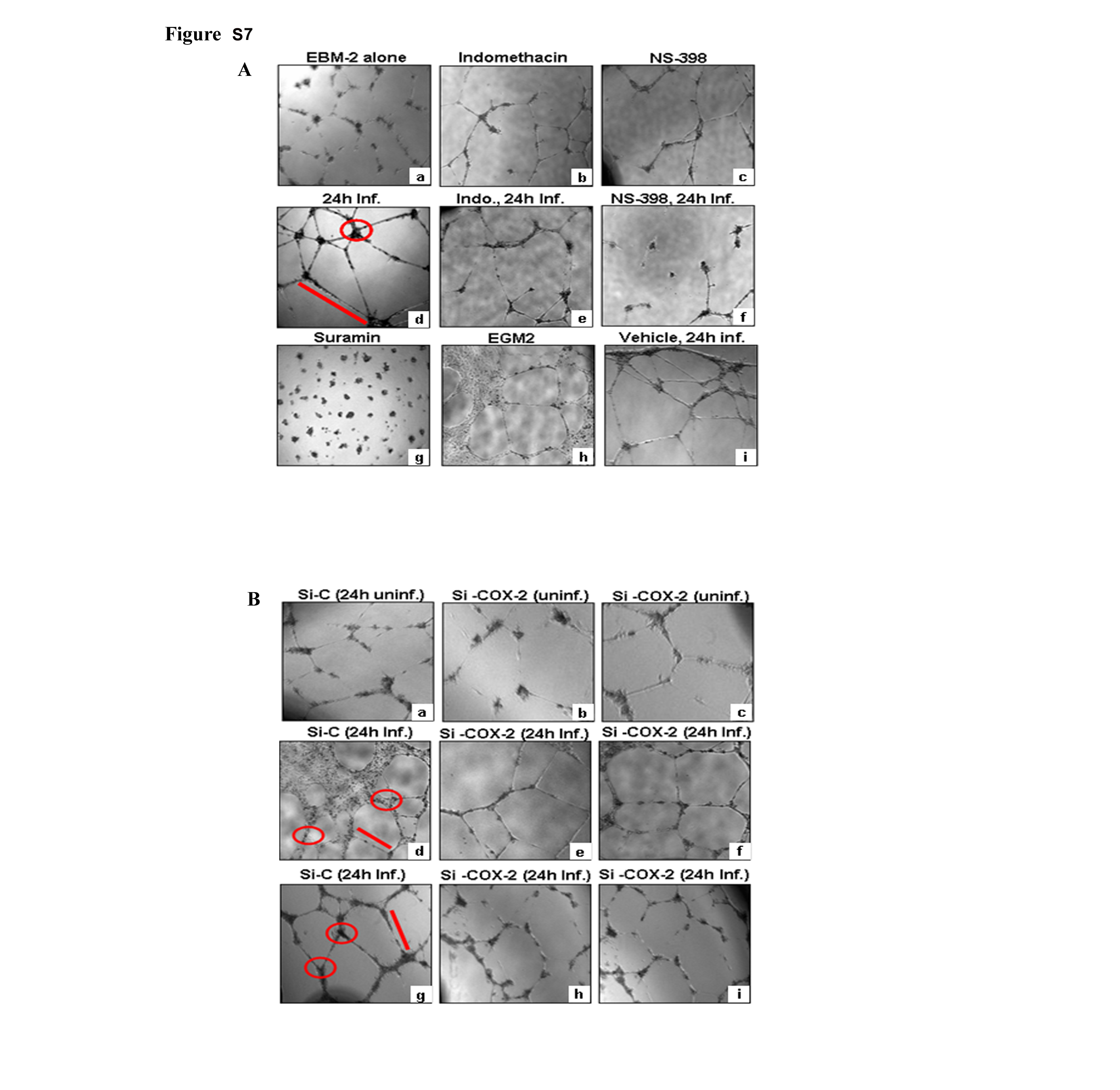

Supplement: Figure S7 — (A) Effect of COX-2 inhibition by Indo or NS-398 on KSHV-induced uninfected HMVEC-d capillary tube formation. Photomicrograph showing HMVEC-d cell tube formation with various supernatants in a matrigel coated 96-well plate. (a) endothelial basal medium; Supernatants were from: (b and c) cells treated with inhibitors alone; (d) cells infected with KSHV for 24h; (e) cells pretreated with Indo (500 µM,1h) and then infected with KSHV for 24h; (f) cells pretreated with the NS-398 (50 µM,1h) and then infected with KSHV for 24h; (g) cells pretreated with the Suramin; (h) cells cultured in the presence of EGM-2 complete growth medium (with serum); (i) cells pretreated with solvent (vehicle) alone and then infected with KSHV for 24h. After 16h incubation, plates were examined for capillary tube formation under an inverted microscope and photographed at 10X magnification. Circles represent branch points/field (connections among cells), and the line represents length of the capillary tubes. Each assay was done in triplicate and each experiment was repeated three times and the qualitative differences were further analyzed by using morphometric analysis in metamorph software to obtain quantitative information regarding tube length. (B) Effect of COX-2 inhibition by si-RNA on KSHV-induced uninfected HMVEC-d capillary tube formation. Photomicrograph showing HMVEC-d cell tube formation with various supernatants in a matrigel coated 96-well plate. Supernatants were from: (a) cells silenced for lamin (control) and then left uninfected for 24h; (b) cells silenced for COX-2 using COX-2-1 construct and then left uninfected for 24h; (c): cells silenced for COX-2 using COX-2-2 construct and then left uninfected for 24h; (d) cells silenced for lamin construct and then infected with KSHV for 24h; (e) cells silenced for COX-2 using COX-2-1 construct and then infected with KSHV for 24h; (f) cells silenced for COX-2 using COX-2-2 construct and then infected with KSHV for 24h. (g, h and i) Addi [file ppat.1000777.s007.tif]

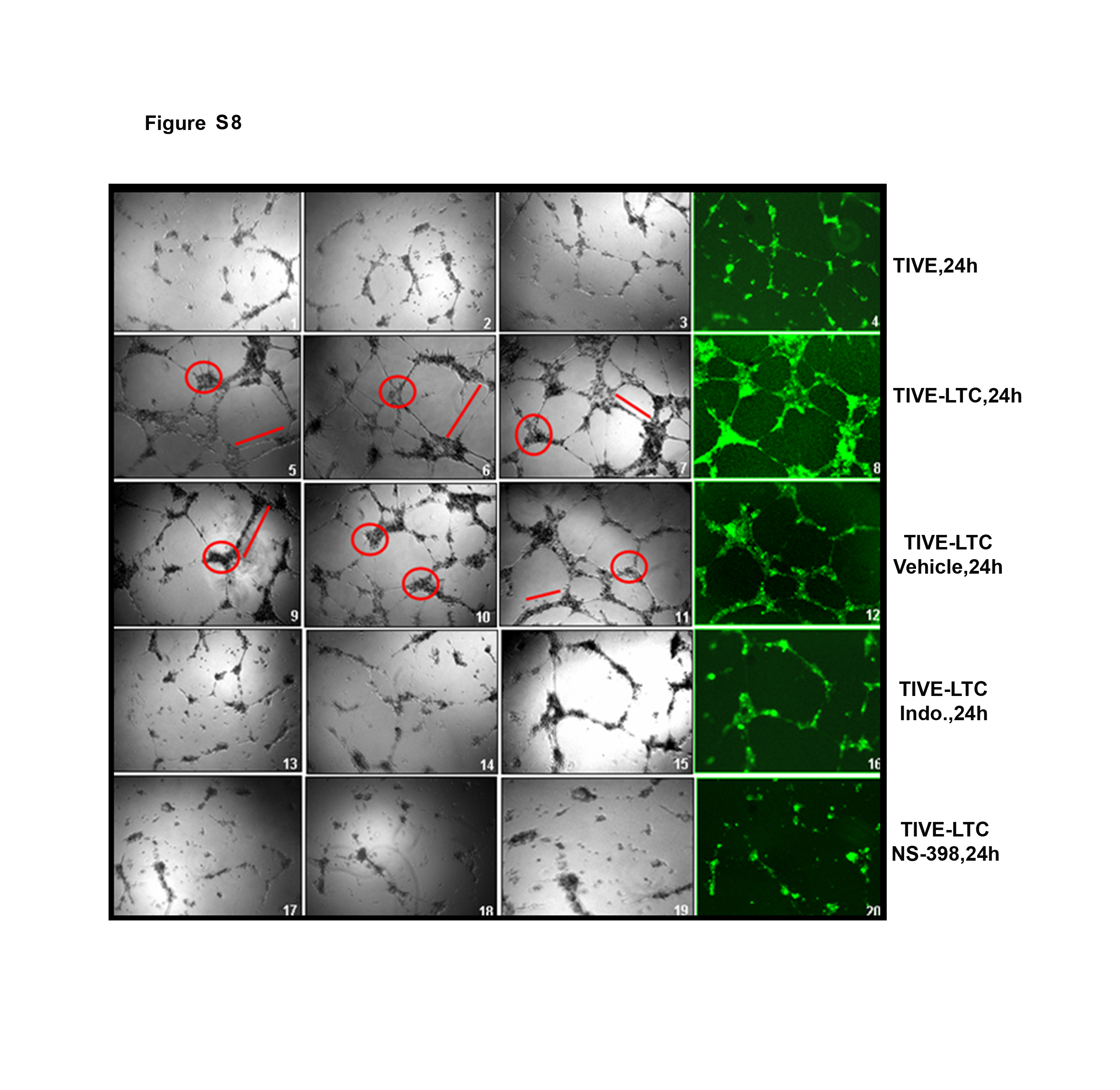

Supplement: Figure S8 — Effect of COX-2 inhibition on the ability of supernatants obtained from latently infected TIVE-LTC cells to form tubular network in uninfected HMVEC-d cells. Photomicrographs showing HMVEC-d cell tube formation with various supernatants in a matrigel coated 24-well plate. Supernatants were from: (1-4) 24h serum starved TIVE cells; (5-8) 24h serum starved TIVE-LTC cells; (9-12) TIVE-LTC cells treated with solvent control (24h); (13-16) TIVE-LTC cells treated with 500 µM Indo (24h); (17-20) TIVE-LTC cells treated with 75 µM NS-398 (24h). Experiment was performed and analyzed as described in Figure S6. Panels 4, 8, 12, 16 and 20 are the same view as 3, 7, 11, 15, and 19, respectively (viewed under UV light). In these experiments, Calcein-AM loaded HMVEC-d cells were used for tube formation. (2.99 MB TIF) [file ppat.1000777.s008.tif]

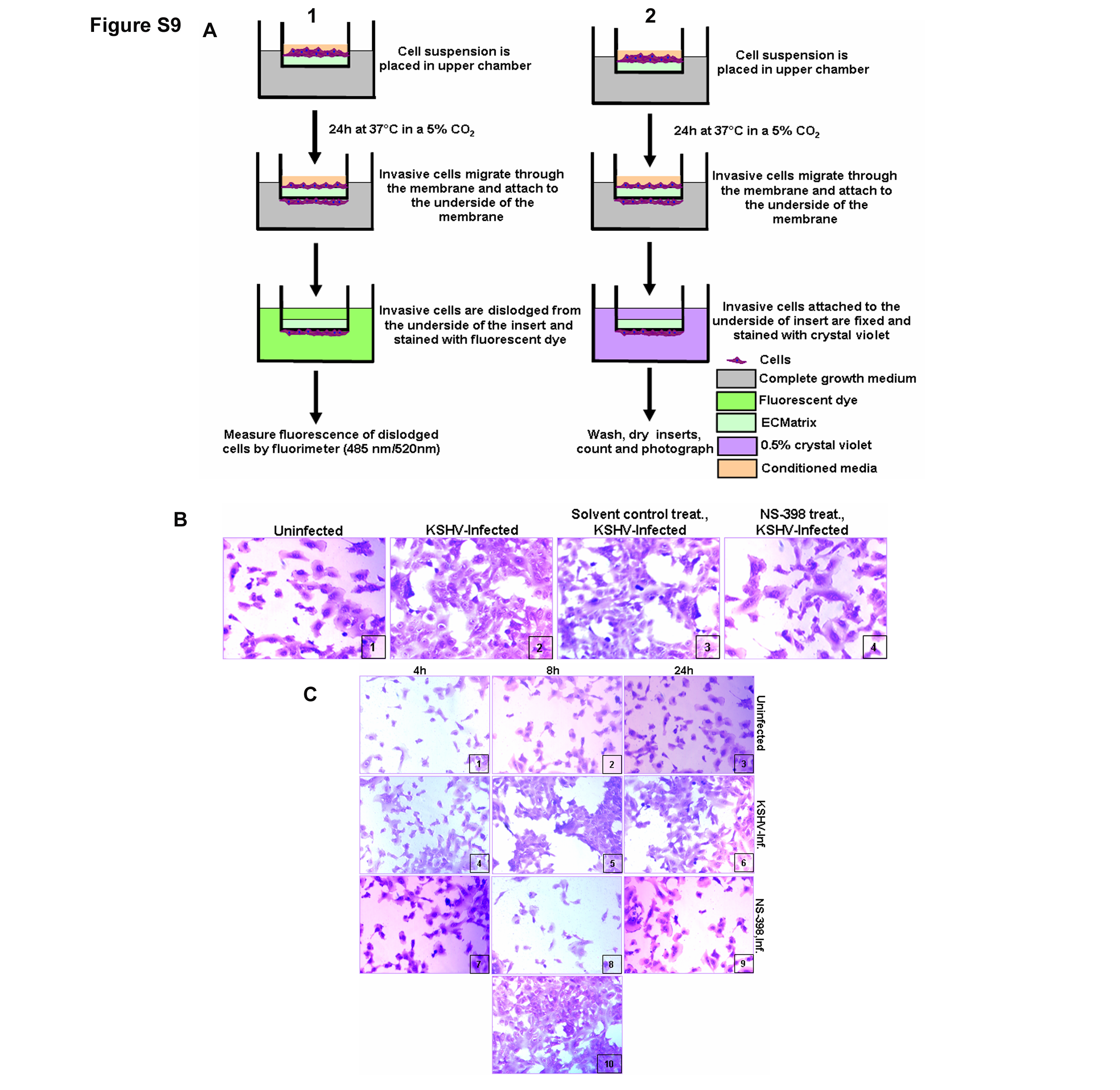

Supplement: Figure S9 — COX-2 regulates KSHV infected HMVEC-d cell invasion via autocrine and paracrine mechanisms. (A) Schematic for invasion assays used; 1) innocyte cell invasion assay and 2) Chemicon cell invasion assay. (B-C) Effect of KSHV infection and COX-2 regulation upon the invasive potential of endothelial cells was measured by Chemicon cell invasion assay as described in Materials and Methods. Representative pictures of uninfected, KSHV infected, solvent control treated and KSHV infected, NS-398 pretreated and then infected HMVEC-d cells migrated through the ECMatrix layer are shown. All invasion assays were carried out over 24h as described in Materials and Methods. Each assay was done in duplicate and each experiment was repeated three times and analyzed by considering five fields. Representative pictures of HMVEC-d cells invaded in the presence of conditioned media obtained from uninfected, KSHV infected, uninfected NS-398 treated, NS-398 pretreated and then KSHV infected for 4h, 8h and 24h. (6.78 MB TIF) [file ppat.1000777.s009.tif]

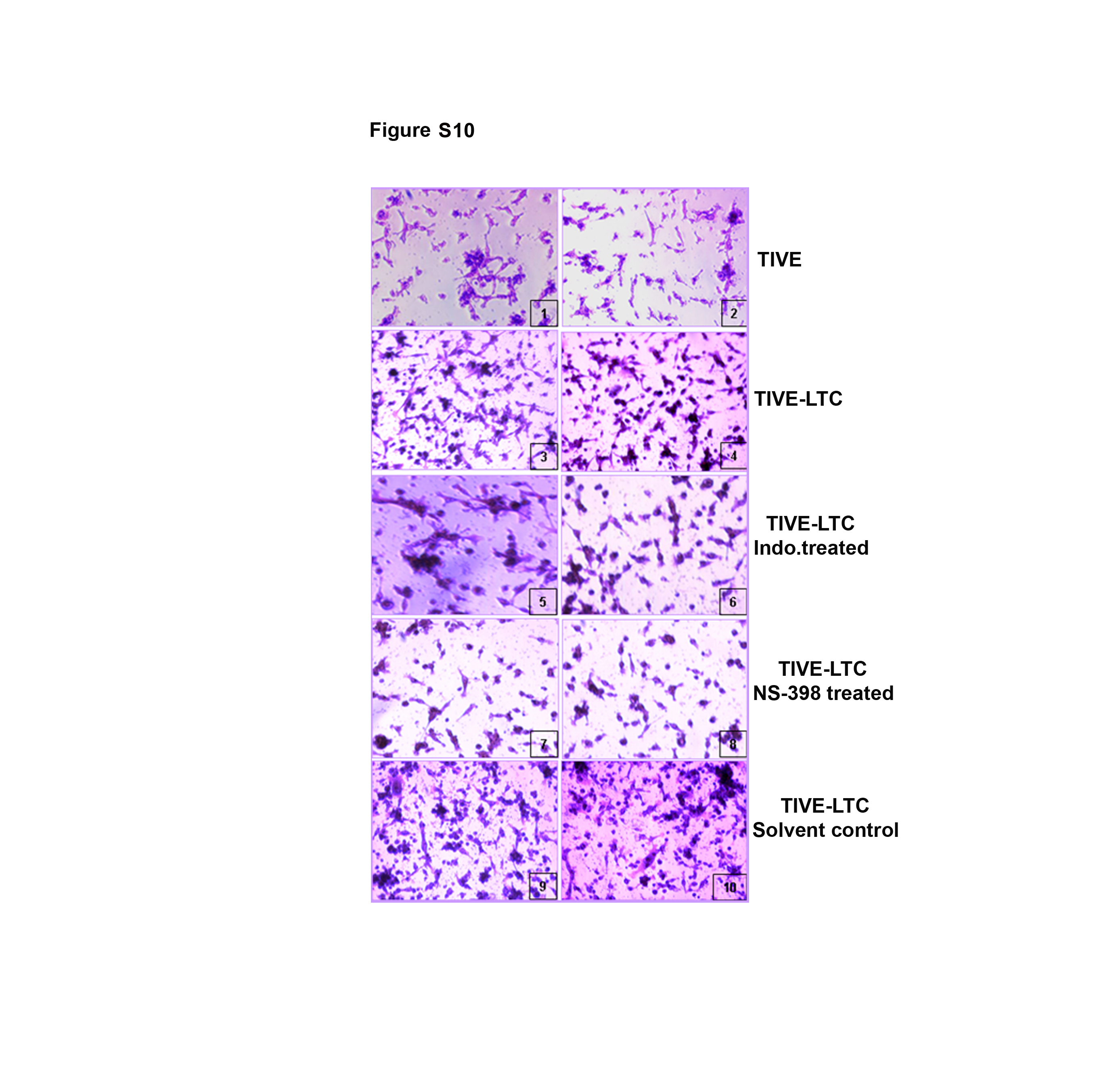

Supplement: Figure S10 — Effect of COX-2 inhibition on invasion of latently infected TIVE-LTC cells. Representative pictures of TIVE, TIVE-LTC untreated, solvent control treated, 500 µM Indo, or 75 µM NS-398 treated TIVE-LTC cells migrated through the ECMatrix layer are shown. All the invasion assays were carried out for 24h as described in Materials and Methods. 1 and 2, 3 and 4, 5 and 6, 7 and 8, 9 and 10 are pictures from duplicate wells. All assays were done by two methods using three independent experiments and analyzing five fields. (7.48 MB TIF) [file ppat.1000777.s010.tif]

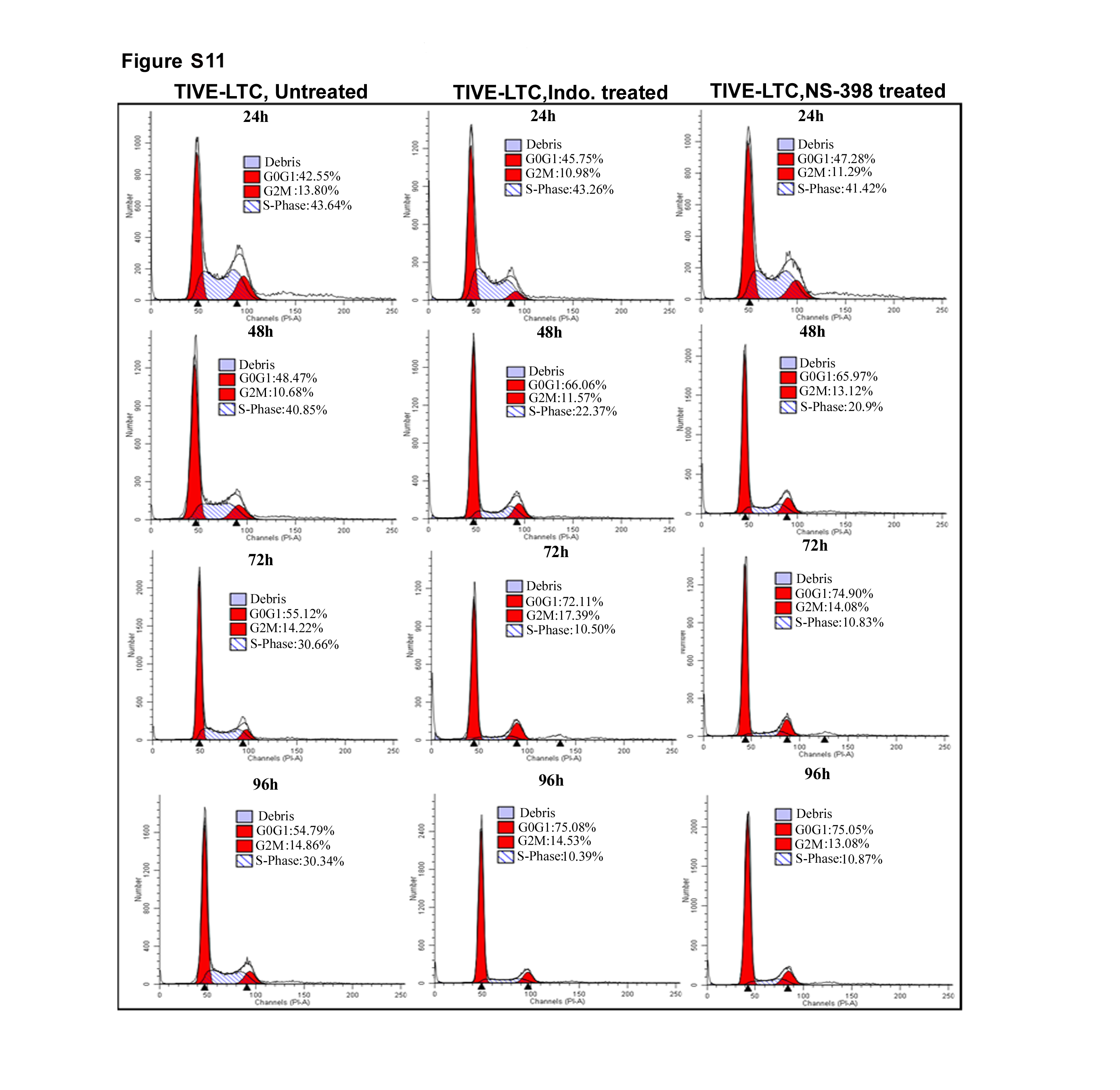

Supplement: Figure S11 — Effect of COX inhibitors on the cell cycle profile of latently infected endothelial cells. Representative pictures for cell cycle analysis after propidium iodide staining of untreated, 500 µM Indo or 75 µM NS-398 treated TIVE-LTC cells for 24h-96h. The percentages of cells at specific cell-cycle phases are indicated and the numbers represent mean values of six independent experiments. (4.10 MB TIF) [file ppat.1000777.s011.tif]

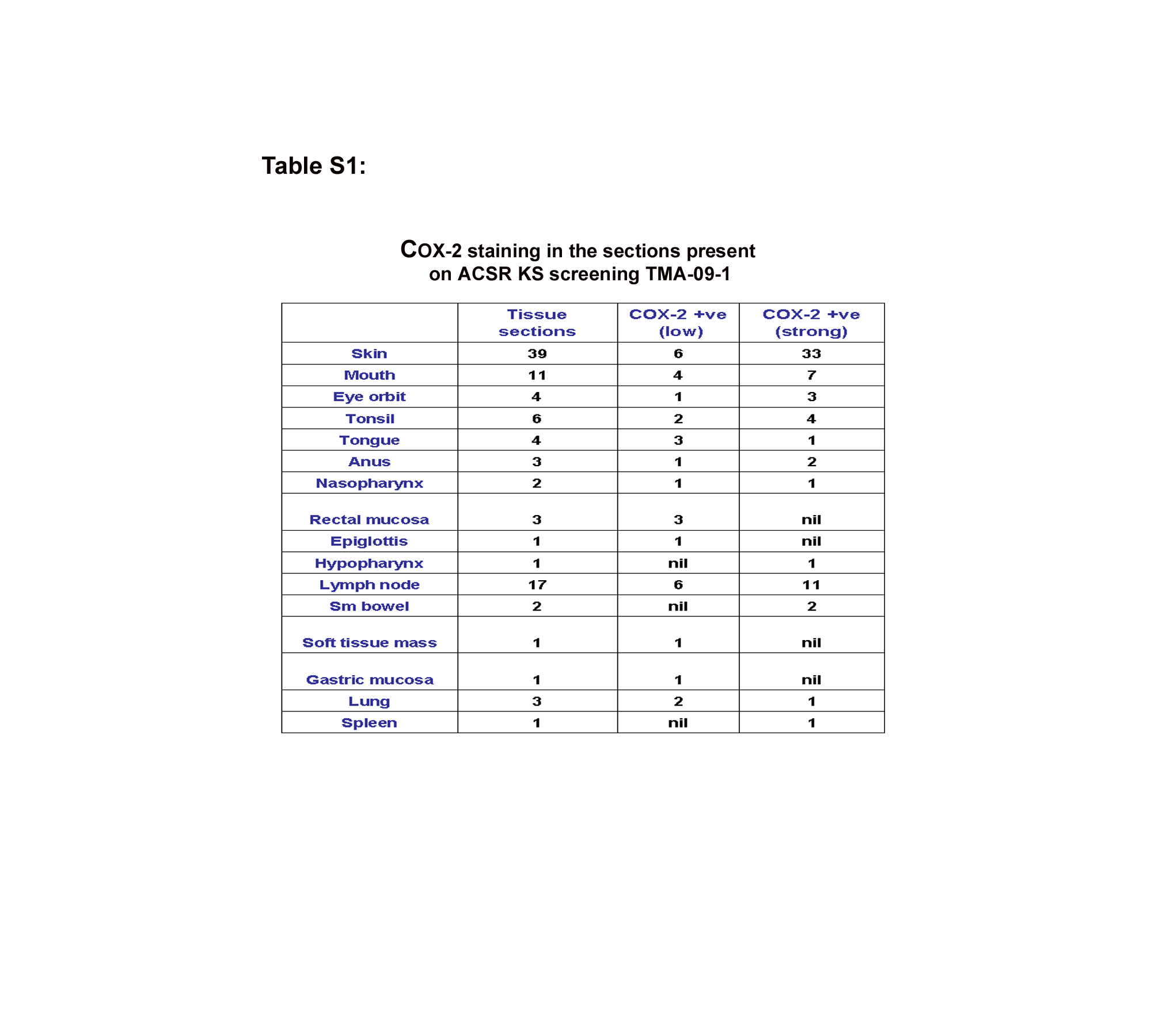

Supplement: Table S1 — COX-2 staining in Kaposi Sarcoma tumor sections (ACSR KS Screening TMA 09-1). (0.19 MB TIF) [file ppat.1000777.s012.tif]

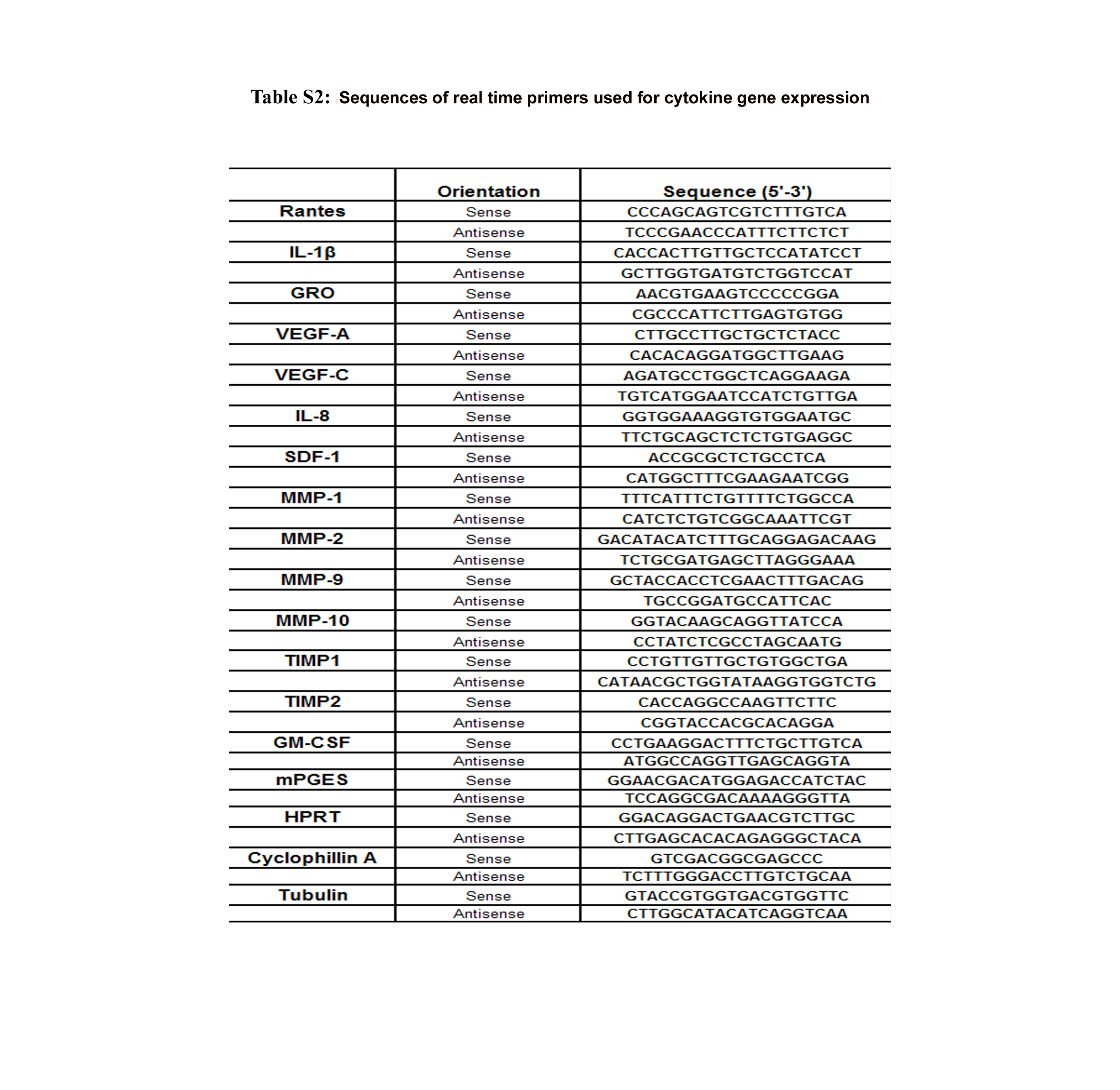

Supplement: Table S2 — Sequences of real time-primers used for cytokine gene expression. (1.98 MB TIF) [file ppat.1000777.s013.tif]

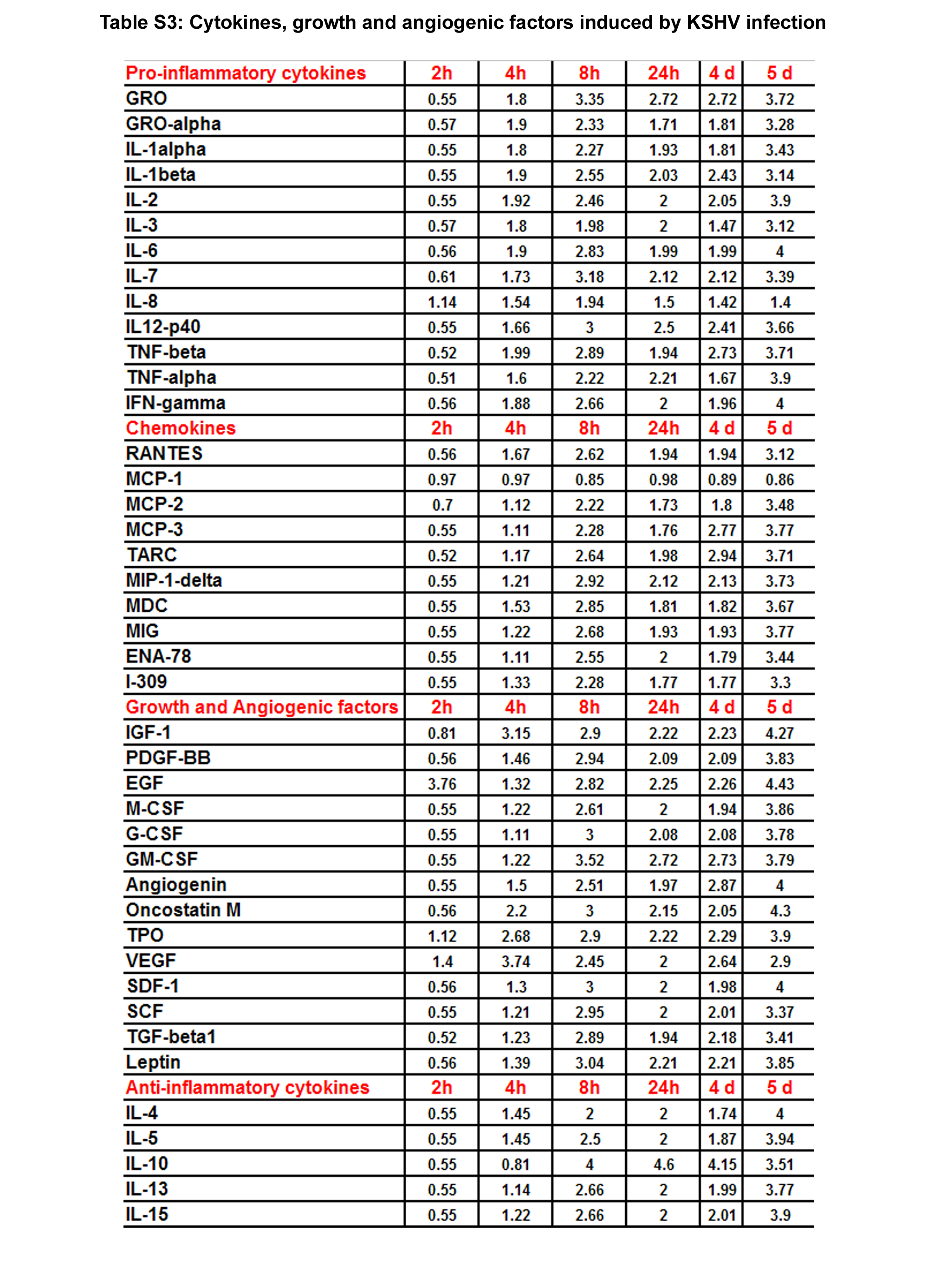

Supplement: Table S3 — Cytokine Profiling was done using Ray Biotech human cytokine antibody 3.1 according to manufacturer's instructions. Arrays were incubated with supernatants o/n at 4°C, and then developed. The densitometry values were substituted in analysis tool and entire array values were normalized to the same background level. Fold induction in cytokine levels was calculated considering levels in uninfected supernatant as 1 -fold. Data is the average of 3 arrays per treatment. (1.97 MB TIF) [file ppat.1000777.s014.tif]

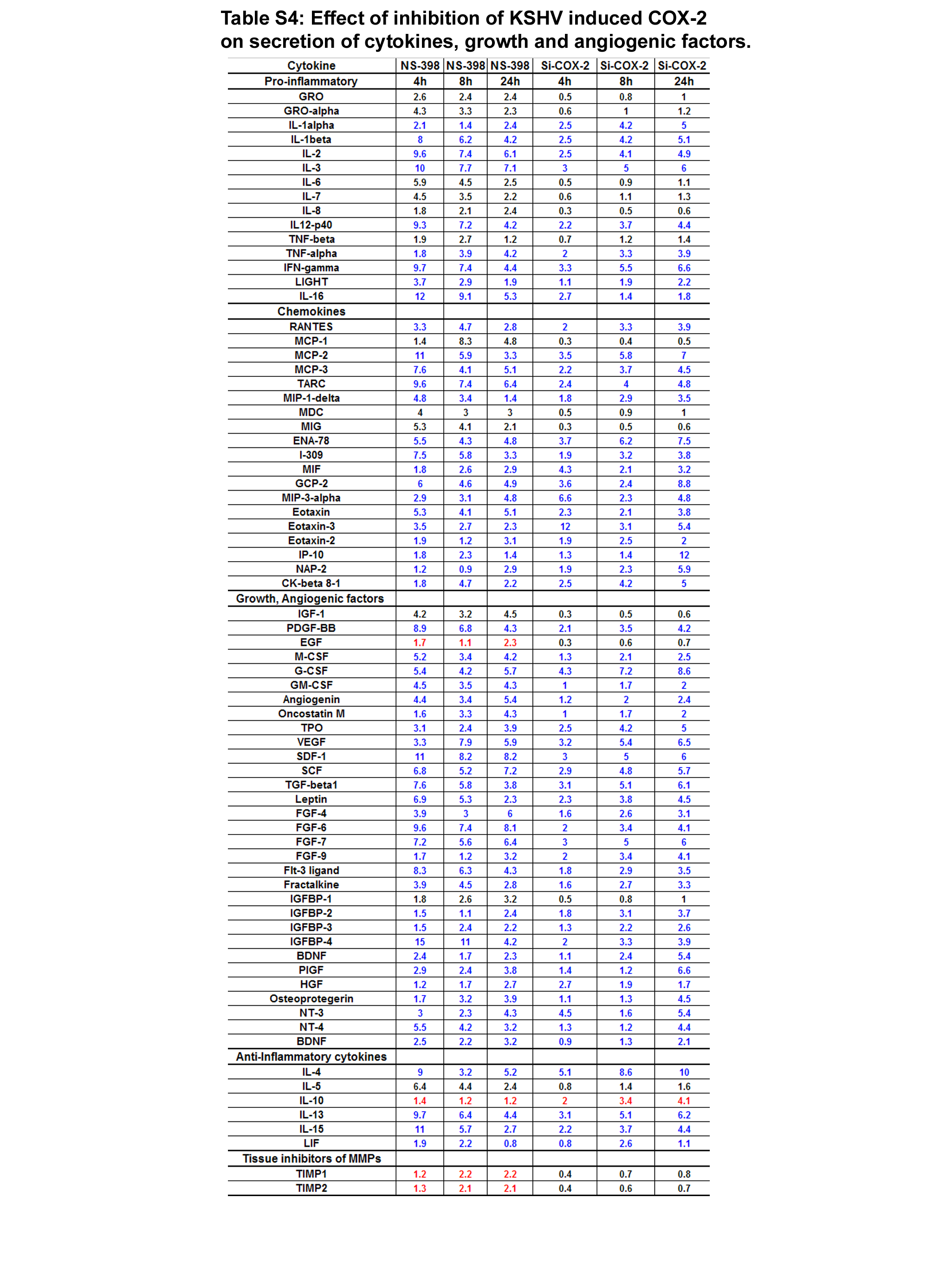

Supplement: Table S4 — Fold reductions upon NS-398 pretreatment before KSHV infection or COX-2 silencing upon KSHV infection are presented and fold reduction was calculated with respect to the levels upon infection at the respective time point of infection. Values in red show the fold induction in levels with respect to their corresponding levels upon infection. Data is divided into groups 1, 2, and 3, which are represented in blue, black and red colors, respectively. Group 1 includes the cytokines inhibited by both kind of COX-2 inhibition (chemical as well as silencing), group 2 includes the cytokines inhibited by chemical inhibitor treatment alone, not reduced by COX-2 knockdown, and group 3 includes the cytokines up-regulated upon COX-2 inhibition. (1.08 MB TIF) [file ppat.1000777.s015.tif]
